# Synergistic interplay between photoisomerization and photoluminescence in a light-driven rotary molecular motor

**DOI:** 10.1038/s41467-022-33177-0

**Published:** 2022-09-30

**Authors:** Ryojun Toyoda, Nong V. Hoang, Kiana Gholamjani Moghaddam, Stefano Crespi, Daisy R. S. Pooler, Shirin Faraji, Maxim S. Pshenichnikov, Ben L. Feringa

**Affiliations:** 1 Stratingh Institute for Chemistry, University of Groningen, Nijenborgh 4, 9747 AG Groningen, the Netherlands; 2 Zernike Institute for Advanced Materials, University of Groningen, Nijenborgh 4, 9747 AG Groningen, the Netherlands; 3 Present Address: Department of Chemistry, Graduate School of Science, Tohoku University, 6-3 Aramaki-Aza-Aoba, Aoba-ku, Sendai, 980-8578 Japan; 4 Present Address: Department of Chemistry -Ångström Laboratory, Uppsala University, Box 523, 751 20 Uppsala, Sweden

**Keywords:** Photochemistry, Organic chemistry, Molecular machines and motors

## Abstract

Photoactuators and photoluminescent dyes utilize light to perform mechanical motion and undergo spontaneous radiation emission, respectively. Combining these two functionalities in a single molecule would benefit the construction of advanced molecular machines. Due to the possible detrimental interaction between the two light-dependent functional parts, the design of hybrid systems featuring both functions in parallel remains highly challenging. Here, we develop a light-driven rotary molecular motor with an efficient photoluminescent dye chemically attached to the motor, not compromising its motor function. This molecular system shows efficient rotary motion and bright photoluminescence, and these functions can be addressed by a proper choice of excitation wavelengths and solvents. The moderate interaction between the two parts generates synergistic effects, which are beneficial for lower-energy excitation and chirality transfer from the motor to the photoluminescent dye. Our results provide prospects towards photoactive multifunctional systems capable of carrying out molecular rotary motion and tracking its location in a complex environment.

## Abstract (web summary)

Combining photofunctionalities in a single molecule is challenging due to inherent detrimental interactions. Here, the authors construct a molecular motor that exhibits photoinduced rotary motion together with bright photoluminescence.

## Introduction

The introduction of light-controlled motion based on molecules, capable of switching between at least two stable forms, offers tremendous opportunities towards the design of photofunctional molecular systems and responsive materials^1^. Inspired by examples from Nature such as chlorophyll present in chloroplasts and retinal in mammalian eyes^2,3^, numerous artificial photoactive molecules have been developed to enable distinct functions such as photoactuators^4–8^, photoredox catalysis^9,10^, photopharmacology^11,12^, and photoluminescence (PL)^13–16^. Combination of multifunctional light-responsive molecules with a sophisticated molecular design like orthogonality is highly desired to boost applications that require multiple functions operating in synergy.

Light-driven artificial rotary molecular motors (MMs) are among the most advanced molecular machines that perform continuous unidirectional 360-degree rotation through sequential photoisomerization and thermal helix inversion (THI) processes with inversions of molecular chirality^17–20^. Powered by light, these MMs work in various environments such as organic solvents^20^, in aqueous media^21^, in liquid crystals^22^, on surfaces^23^, as artificial muscles^24,25^ and in solid materials^26^. Due to this unique feature, they are considered promising candidates to perform future nanoscale mechanical manipulation, for instance in molecular surgery, biological cells, and nanomaterials^27^. To fully exploit their mechanical work, it is also essential to track the location and to monitor the rotation of the MM present in a complex environment, as in this way, precise control over the MM movement can be achieved.

PL is an excellent technique to probing, visualizing and localizing the motor position without physically interacting with the molecules, altering the surrounding environment or generating byproducts. For instance, the visualization of the controlled motion of single biological MMs has been widely studied with well-defined and universally used tools such as PL microscopy^28–34^. However, despite these opportunities, the currently designed artificial MMs usually present weak PL with an associated quantum yield (QY) not exceeding 10^−4^, a value not compatible with tracking or visualization purposes^35^.

To overcome this issue, functionalizing MMs by combining it with a chromophore exhibiting efficient PL is highly challenging because of interference and compatibility but would offer an attractive strategy due to its conceptual simplicity and ease of material’s selection. Despite the possible detrimental interactions between the individual functioning parts^36–38^, a number of studies have reported successful combinations of multiple photofunctional molecules.^39–48^ For example, Kawai and coworkers achieved light-controlled intensity switching of circularly polarized luminescence by hybridizing pyrene fluorophores and a tetrathiazole photoswitch^42^. Chen et al. used a long linker between a dye and a motor to avoid their unwanted interactions^49^; however, a synergistic effect, resulting from the combination of molecules rather than a sum of properties of the individual constituents, would also be beneficial for expanding the system practicability.

**Fig. 1:**
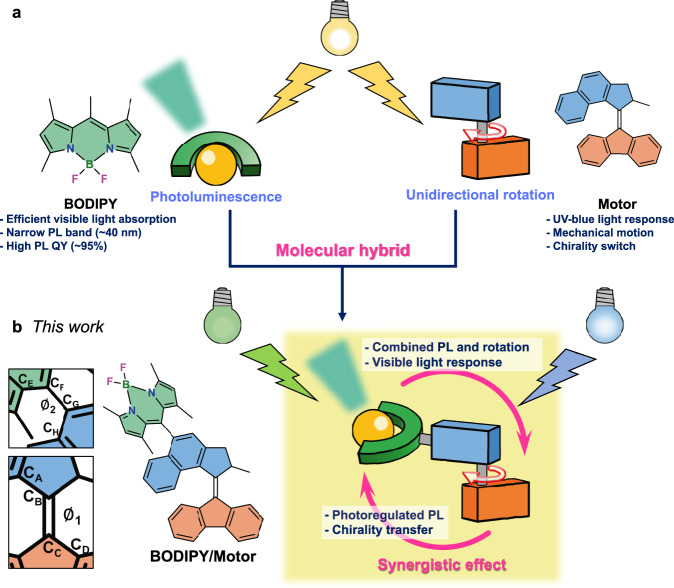
Schematic illustration of combination of a photoluminescent (PL) dye and a molecular rotary motor. **a** Chemical structures of conventional photofunctional molecules, a bright PL dye (**BODIPY**) and a light-driven MM (**Motor**). **b** Chemical structure of molecular hybrid **BODIPY/Motor** studied in this work. The dihedral angles around the rotational axle of the motor core (∅_1_) and between the BODIPY and the upper half of the MM (∅_2_) are represented in the left panel.

Here, we present a molecular design, successfully combining light-powered rotary motion and efficient PL within a single molecular structure, where a PL dye is directly attached to an operating rotary MM (Fig. 1). Contrary to the previous design which used a long linker between a dye and a motor^49^, the current design features a more compact structure but still a moderate interaction between the two functional parts due to their (almost) orthogonal alignment. By means of UV/vis absorption, PL, transient absorption and nuclear magnetic resonance (NMR) spectroscopies, and spin-flip time-dependent density functional (SF-TDDFT) calculations, we demonstrated that the MM with the PL dye attached maintains both functions, motor-rotation and PL. Furthermore, the degree of these functions can be tuned and interchanged by varying the solvent polarity^50,51^, which enables the hybrid of a MM and a PL dye for applications that require a preferential function of motor-rotation or PL. Interestingly, the synergistic effects derived from the two photofunctional moieties provided remarkable benefits such as visible-light-driven motor-rotation and induction of chirality with light-controlled helicity at the otherwise achiral PL dye.

## Results and discussions

### Design and synthesis of BODIPY/Motor

**Fig. 2:**
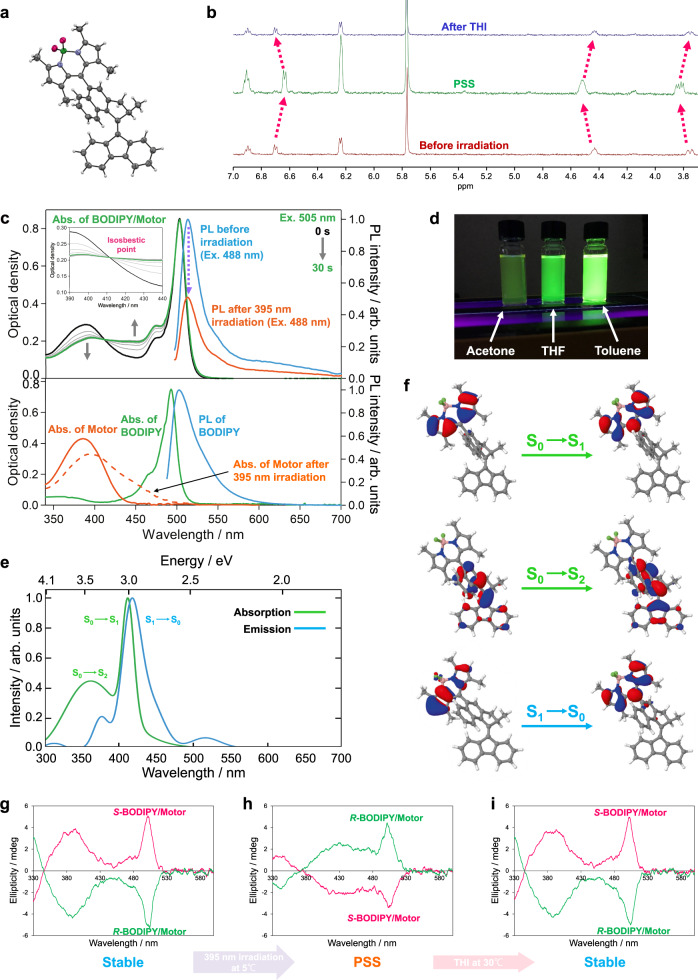
Light-response behavior of BODIPY/Motor. **a** Crystal structure of **BODIPY/Motor** with a thermal ellipsoid set at the 50% probability level. Black: carbon; white: hydrogen; blue: nitrogen; green: boron, magenta: fluorine. **b** Stack of ^1^H NMR spectra of **BODIPY/Motor** before irradiation with a 395 nm LED, at photostationary state (PSS) and after completed thermal helix inversion (THI) measured in acetone-*d*_6_ at –40 °C. The dashed arrows indicate proton peak shifts upon isomerization of the motor core. **c** Upper panel: UV/vis absorption spectral change of **BODIPY/Motor** with 505-nm irradiation and PL spectra of **BODIPY/Motor** before and right after (10 s) 395-nm irradiation for 1 min in acetone. The excitation wavelength of the PL spectra was 488 nm. The 505-nm and 395-nm irradiations were provided by light-emitting diodes (LEDs) with the output wavelength centered at 505 nm and 395 nm, respectively. Lower panel: Reference spectra of **Motor** and **BODIPY** in acetone. **d** Photographs of samples of **BODIPY/Motor** in various solvents under 365 nm UV light. **e** Calculated absorption and emission spectra of **BODIPY/Motor** in acetone. **f** Natural transition orbitals (NTOs) for the S_0_ → S_1_, S_0_ → S_2_ and S_1_ → S_0_ transitions of the **BODIPY/Motor** stable isomer in acetone. Circular dichroism (CD) spectra of ***R*-** and ***S*-BODIPY/Motors** in acetone before irradiation (**g**), 10 sec (**h**) and 10 min (**i**) after reaching the PSS by irradiation with a 395-nm LED. The samples before irradiation and 10 sec after reaching the PSS was kept at 5 °C and afterward kept to 30 °C for 10 min.

As a prototypical model, we selected an overcrowded alkene 2nd generation MM^52,53^ to be combined with a derivative of the well-known boron-dipyrromethene (BODIPY) dyes characterized by high PLQY (~95%)^54^, narrow PL band (~40 nm)^55^, high extinction coefficient ($> 1\cdot {10}^{5}\,{{{{{{\rm{M}}}}}}}^{-1}{{{{{{\rm{cm}}}}}}}^{-1}$)^56^ and excellent photostability^56,57^ (Fig. 1a). With these unique PL properties, BODIPY and its derivatives have been widely used in imaging and sensing applications^58,59^. Furthermore, BODIPY emits in the green region of the spectrum, which is beyond the absorption of the motor; therefore, energy transfer from the BODIPY to the motor is likely ruled out. To obtain the light-driven MM capable of bright PL, the *meso*-position carbon of a BODIPY is chemically attached to the upper half of the motor (**BODIPY/Motor**, Fig. 1b). Similar to Hecht’s molecular design^60^, the almost perpendicular connection between the BODIPY plane and the naphthalene upper half of the MM suppresses the direct π-system involving the conjugation of the two functional moieties, still maintaining “moderate” interactions between the two moieties to obtain the synergistic effect (vide infra). The synthesis of the target hybrid **BODIPY/Motor** is outlined in Supplementary Fig. 1 and the molecular structure was fully characterized by NMR spectroscopy, high-resolution mass spectrometry and single-crystal X-ray analysis (Fig. 2a).

### Light-driven motor-rotation

First, we characterized the photochemical and thermal isomerization processes of **BODIPY/Motor** by ^1^H NMR studies (Fig. 2b and Supplementary Figs. 3–6). Figure 2b shows the spectral change observed in acetone upon 395 nm irradiation. The peak intensity of the protons corresponding to the stable state of the motor decreased after irradiation and new absorptions ascribed to the metastable isomer of the motor emerged. A photostationary state (PSS) was reached after 20 min and an almost complete isomerization was achieved with the stable:metastable isomer ratio of 4:96.

After confirming the rotary function of **BODIPY/Motor**, its optical properties were investigated by steady-state UV/vis absorption spectroscopy. In addition to **BODIPY/Motor**, a bare BODIPY (**BODIPY**) and a 2nd generation MM (**Motor**) were examined as reference samples (Fig. 1a). Figure 2c shows absorption spectra of **BODIPY/Motor** as well as the reference samples in acetone. The absorption spectrum for **BODIPY/Motor** at the stable state (Fig. 2c, black line) consist of two prominent absorption bands with maxima at ~500 nm and ~390 nm (Table 1), which show clear contributions of the BODIPY and the motor, respectively. Furthermore, no substantial changes in the shape of the absorption spectra of **BODIPY/Motor** in different solvents were observed (Supplementary Fig. 7). It is also noteworthy that compared to **BODIPY**, the absorption band for **BODIPY/Motor** shows a slight bathochromic shift. These results indicate a weak intramolecular interaction between the two units but also imply that these moieties remain functioning as individual entities.

**Table 1:** Summary of absorption and PL peaks, Stokes shifts, PL and photoisomerization quantum yields (QYs)

Compound	Solvent	Abs. peak (nm)	PL peak (nm)	Stokes shift (eV)	PLQY (%)	Photo-isomerization QY (%)
**Motor**	Toluene	389	–	–	${ {\sim} 10}^{-4}$^a^	18^b^
	Acetone	386	–	–		12^b^
**BODIPY**	Toluene	500	510	0.050	95^c^	–
	Acetone	493	502	0.053	–	–
**BODIPY/Motor**	Toluene	508	519	0.055	54	12 (3)^d^
	Acetone	503	513	0.051	2.6	22 (4)^d^

The excitation wavelengths for the measurements of the PL and photoisomerization QYs were 488 nm and 395 nm, respectively.

^a^Adapted from ref. 35.

^b^Measured with a 365 nm excitation wavelength.

^c^Adapted from ref. 48.

^d^QYs of photoisomerization for the stable → metastable transformation. The numbers in parentheses are the photoisomerization QYs measured with a LED light source with the output wavelength at 535 nm and a full width at half maximum (FWHM) of ~24 nm.

The calculated UV/vis absorption spectra of **BODIPY/Motor** stable isomer in acetone and toluene feature two main absorption bands (Fig. 2e and Supplementary Fig. 7c, d). The S_0_ → S_1_ and S_0_ → S_2_ transitions are blue-shifted by ~0.5 eV (~90 nm) and 0.2 eV (26 nm) in both solvents, respectively, compared to the corresponding experimental absorption bands, which are in line with the error range of the TDDFT methods. The nature of these two transitions were identifed by the natural transition orbitals (NTOs) (Fig. 2f and Supplementary Fig. 8). The results indicate that the hole/electron NTOs of the S_1_ transition of the stable isomer in both solvents are mainly localized on the BODIPY, while for the S_2_ transition, they are mainly located on the motor.

In order to demonstrate the photochemically driven rotational motion of **BODIPY/Motor**, a solution sample of **BODIPY/Motor** in acetone was subjected to irradiation with 395 nm light provided by a light-emitting diode (LED). The absorption spectra were measured at various intervals from 0.5 s up to 30 s. (Fig. 2c, gray and green lines). Upon irradiation, photoisomerization of **BODIPY/Motor** leading to the formation of the metastable isomer was observed^61^, accompanied by a decrease in the absorption region of 340–410 nm and an increase in the 410–480 nm region. Similar changes were observed in the absorption spectra of **BODIPY/Motor** in toluene and THF (Supplementary Figs. 10–13). After removal of the light source and keeping the sample in dark, the metastable isomer underwent THI to proceed to the second, chemically identical, stable isomer and thereby completed a unidirectional 180-degree rotation, i.e., the first half of a full rotary cycle^20^. These photochemical and thermal isomerization steps can be repeated multiple times without noticeable degradation, indicating excellent photostability of **BODIPY/Motor**. Kinetic studies determined that the thermodynamic parameters of THI (Supplementary Table 5) are consistent with our previous studies on 2nd generation MMs^62^, further supporting the uncompromised rotation function of **BODIPY/Motor**.

### Solvent-dependent photoisomerization and PL

The photochemical reaction to form the metastable isomer becomes less favorable in apolar toluene, which gave a 60:40 ratio of isomers. This result indicates that the photoisomerization process is strongly dependent on solvent properties, such as polarity or viscosity^50,61^. To further quantify the photoisomerization reaction, we measured the QYs of **BODIPY/Motor** isomerization in acetone and toluene with 390 nm excitation (Supplementary Section 7). **BODIPY/Motor** in toluene shows a photoisomerization QY of 12%, which is similar to bare **Motor** (Table 1). However, a substantial increase in the photoisomerization QY to 22% was obtained for **BODIPY/Motor** in acetone. It shows that **BODIPY/Motor** performs rotation more efficiently in a polar solvent (e.g., acetone), which opens the prospect to control the rotation functionality via selection of the solvent.

Next, we studied the PL properties using steady-state PL spectroscopy. Figure 2c (blue and orange lines) shows PL spectra of **BODIPY/Motor** before and immediately after 10 sec of irradiation with 395 nm light. Both PL spectra of **BODIPY/Motor** exhibit intense green PL in the 500‒600 nm region originated merely from the BODIPY. This result is confirmed by similar Stokes shifts of ~50 meV for **BODIPY/Motor** and bare **BODIPY** (Table 1) and no excitation-wavelength dependence on the PL spectra of **BODIPY/Motor** (Supplementary Section 5). Here, the PL spectra of **BODIPY/Motor** in acetone are broader compared with that of bare **BODIPY** probably because of the structural flexibility at the excited state. SF-TDDFT calculations were performed to determine the origin of PL of **BODIPY/Motor** in toluene and acetone. As is clear from Fig. 2e (and Supplementary Fig. 7c), the shape of the calculated emission spectrum of **BODIPY/Motor** in toluene is quite similar to that in acetone, which exhibits a strong band with a maximum at ~417 nm blue-shifted by ~103 nm compared to the experimental data. Additionally, Fig. 2f (and Supplementary Fig. 8) shows that NTOs of the S_1_ → S_0_ transition of **BODIPY/Motor** stable isomer in both solvents are mainly localized on the BODIPY.

To quantify PL of **BODIPY** upon chemically attaching to the motor, we set out to determine PLQYs of **BODIPY/Motor** in different solvents. **BODIPY/Motor** in toluene displays a PLQY as high as 54%. However, the PLQY of **BODIPY/Motor** is much lower with acetone used as the polar solvent (2.6%, Table 1). This substantial decrease in the PLQY is clearly opposite to what was observed for the photoisomerization QYs and suggests that a large portion of the excitation energy contributes to the photoisomerization process in polar solvent. Furthermore, **BODIPY/Motor** at the PSS has even lower PLQYs (~2 fold) compared to that at the stable state (see data in Supplementary Table 4). This result indicates that the PL intensity can be controlled from bright to gloomy by switching the photoisomerization state of the MM.

### Computed potential energy surfaces and excited-state dynamics

**Fig. 3:**
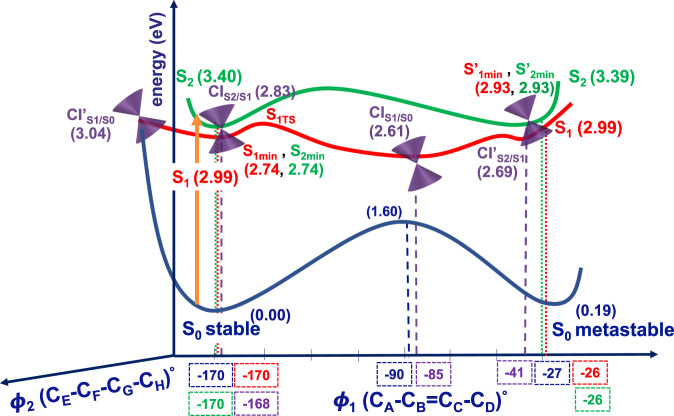
Theoretical mechanism for photoisomerization and PL of BODIPY/Motor. Schematic representation of the potential energy surfaces (PESs) of **BODIPY/Motor** along the ∅_1_ and ∅_2_ dihedral angles in acetone. The ground state (S_0_), first (S_1_) and second (S_2_) excited states are depicted in dark blue, red and green, respectively. The energies are relative to **BODIPY/Motor** stable S_0_. The purple cones represent the conical intersections (CIs).

In order to understand the photochemical reactions of **BODIPY/Motor** and the role of solvents, we analyzed the ground (S_0_) and first two excited (S_1_ and S_2_) states potential energy surfaces (PESs) of **BODIPY/Motor** in gas phase, acetone, and toluene along two reaction coordinates (dihedral ∅_1_ and ∅_2_, Fig. 1)^63,64^. For the sake of clarity, we present the calculated PESs for **BODIPY/Motor** in acetone (Fig. 3); similar PESs can be found in Supplementary Fig. 14.

The photoisomerization dynamics are essentially controlled by the excited-state minima and various conical intersections (CIs). It is evident from Fig. 3 that the S_0_ PES has two minima connected through the transition state with an energy barrier of 1.6 eV (36.9 kcal mol^−1^) at ∅_1_ = −90°. This significant barrier excludes the thermal interconversion mechanism.

Upon 395 nm irradiation, **BODIPY/Motor** stable isomer is excited to the S_2_ with an energy of 3.40 eV. Then, the molecule relaxes to the S_1_ passing the CI_S2/S1_, with an energy of 2.83 eV at ∅_1_ = −168°. This, the so-called internal conversion, is common when the CI is within the Franck–Condon (FC) region and no significant energy barrier is observed. From this crucial CI, the molecule goes through vibrational relaxation towards S_1min_ (at ∅_1_ = −170° and energy of 2.74 eV). Similarly, upon 395 nm irradiation, **BODIPY/Motor** metastable isomer is excited to the S_2_ with an energy of 3.39 eV from where it undergoes vibrational relaxation towards S_1min_ and subsequently hits the CI’_S2/S1_. From the S_1min_ of **BODIPY/Motor** stable isomer_,_ the system evolves directly towards CI_S1/S0_ (at ∅_1_ = −85°) along the photoisomerization coordinate, which provides a funnel of ultra-fast access to the S_0_, from where the system can evolve to either **BODIPY/Motor** stable or metastable isomer.

Upon 505 nm irradiation, **BODIPY/Motor** stable isomer is excited to the S_1_ with an energy of 2.99 eV. From the FC region, it evolves directly towards CI’_S1/S0_ along ∅_2_ reaction coordinate by passing a tiny energy barrier of 0.05 eV in acetone. Interestingly, we observed a butterfly conformation of the BODIPY in this critical CI’_S1/S0_, which is a signature of **BODIPY** itself^65^. We found that the energetic accessibility of the CI’_S1/S0_ along **BODIPY** butterfly motion strongly depends on the solvent. In the gas phase, the CI’_S1/S0_ is at 4.44 eV which is 1.44 eV higher than the FC region. However, in the presence of the solvent the CI’_S1/S0_ is significantly lowered in energy by 1.40 eV and 1.36 eV in acetone and toluene, respectively, which is only ~0.05 eV higher than the FC region. It should be noted that the CI’_S1/S0_ being energetically accessible plays an essential role in the PL quantum yields of **BODIPY/Motor** in solvents, since it opens a pathway for the radiationless decay to **BODIPY/Motor** stable that potentially competes with the PL. Namely, either the excess of vibrational energy after photoexcitation enables the molecule to access the CI’_S1/S0_ with subsequent transition to S_0_ of **BODIPY/Motor** stable isomer resulting in low PLQY, or the molecule can relax towards the S_1min_ from where the emission takes place, indicating a large PLQY. Although **BODIPY/Motor** displayed higher PLQY in toluene (54%) compared to acetone (2.6 %, polar solvent) and thus the CI’_S1/S0_ is expected to be energetically less accessible in toluene, our calculations did not locate the CI’_S1/S0_ to be energetically higher in toluene than acetone. This can be attributed to the explicit solvent-solute interactions, which are missing in the C-PCM implicit solvent model, and their effects on the CI’_S1/S0_ energetics. Moreover, there exist an energy barrier on S_1_ along the photoisomerization coordinate (∅_1_) toward the CI_S1/S0_ that determines the relative rates of photoisomerization and PL in acetone and toluene. Relaxed PES scan of the S_1_ along ∅_1_ dihedral angle (see Supplementary Fig. 15) revealed a lower barrier in acetone (0.09 eV) compared to toluene (0.23 eV), that speaks in favor of a more efficient and higher photoisomerization QY in acetone, thus suppressing PL. This is in line with the experimental findings, i.e., QYs of photoisomerization being 22% and 12% in acetone and toluene, respectively. Additionally, there exists a slight partial charge separation (see Supplementary Table 10) in both excited-state pathways going from S_1TS_ towards CI_S1/S0_ as well as from the S_1min_ towards CI’_S1/S0_, which can further support why the S_1TS_ and CI’_S1/S0_ structures are more energetically stabilized in the polar solvent.

**Fig. 4:**
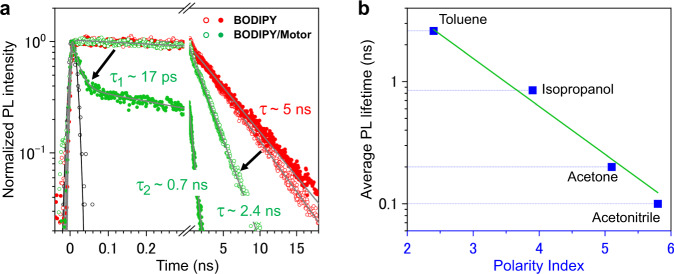
Depopulation of the S_1_ excited state of BODIPY/Motor in different solvents. **a** Time-resolved PL transients of **BODIPY** (red) and **BODIPY/Motor** (green) in toluene (open dots) and in acetone (filled dots) under 510 nm excitation. The PL transients were obtained by integrating the PL maps (Supplementary Fig. 16) in the $520-600{{{{{\rm{nm}}}}}}$ spectral region, where PL of **BODIPY** occurs. The gray curves show the fits to exponential functions convoluted to the Gaussian apparatus function. The fitting parameters of the exponential functions are summarized in Supplementary Table 11. The black arrows depict the PL quenching upon attaching the motor core to **BODIPY**. The black open dots and curve depict the apparatus function at 510 nm and the fit to the Gaussian distribution, $y=\frac{1}{\sqrt{2{{{{{\rm{\pi }}}}}}{\sigma }^{2}}}{e}^{-\frac{{t}^{2}}{2{\sigma }^{2}}}$ with $\sigma=10{{{{{\rm{ps}}}}}}$. **b** Average PL lifetime of **BODIPY/Motor** as a function of the solvent polarity index. The polarity indexes and viscosity values of the solvents are summarized in Supplementary Table 12. The green line shows the best fit to a single-exponential function, ${\tau }_{{ave}}={{\exp }}(-{pt})$, with $p=0.9$, to the average PL lifetime ${\tau }_{{ave}}$ as a function of the solvent polarity index $p$.

To prove that the nature of the solvent can result in adjustment of the barrier height toward the CI_S1/S0_, we conducted time-resolved PL spectroscopy of **BODIPY/Motor** in different solvents to obtain excited-state dynamics (Fig. 4a). PLs of **BODIPY** in toluene and acetone decay with a similar lifetime of $\sim 4.8-5.3{{{{{\rm{ns}}}}}}$, which is in line with a previous study^54,66^. No effect of the solvent properties on PL of **BODIPY** alone was observed (Supplementary Section 9), ruling out additional pathways such as population of intramolecular charge-transfer states in **BODIPY**. When the motor is chemically attached to **BODIPY**, PL is shortened to $2.4{{{{{\rm{ns}}}}}}$ in toluene (Fig. 4a; open green dots). The shortening in PL time demonstrates the depopulation of the excited state in **BODIPY/Motor**, which is in agreement with the decrease in the PLQY of **BODIPY/Motor** (~54%) as compared to **BODIPY** (95%, Table 1). Remarkably, when acetone is used as the solvent, the PL of **BODIPY/Motor** exhibits distinct bi-exponentiality, with the fastest decaying component having a lifetime of $\sim 17{{{{{\rm{ps}}}}}}$ (Fig. 4a; filled green dots), indicating a much faster excited state depopulation. This result is also confirmed by femtosecond transient absorption spectroscopy (Supplementary Section 10). Therefore, the shortening in PL is clearly associated with the increase in the photoisomerization QY, which is the result of the lower energy barrier on S_1_ toward the CI_S1/S0_ along the photoisomerization coordinate.

Figure 4b shows correlation of the average PL lifetime of **BODIPY/Motor** and solvent polarity. With the increase in the solvent polarity index (from toluene to acetonitrile), the average PL lifetime decreases exponentially from 2.6 ns to ~0.1 ns. No correlation between the solvent viscosity and the PL lifetime of **BODIPY/Motor** was observed (Supplementary Section 9.3). Therefore, we conclude that solvent polarity is the leading factor to determine the yield of both rotation and PL functions.

### Synergistic effects on the excitation wavelength and polarization

Considering that the calculated PESs depict that the photoisomerization of **BODIPY/Motor** occurs via the S_1_ after relaxation from the S_2_, it is implied that there is no need to directly excite the motor and there must be an option to deliver the wavepacket to the CI_S1/S0_ with a lower energy, i.e., exciting the molecular system directly to the S_1_. This implication motivated us to examine the rotation function of **BODIPY/Motor** under green light (~505 nm), which is beneficial due to its deeper penetration depth in biological tissue and less photochemical side effects in biological systems and soft materials. Remarkably, we noticed a similar spectral shape change in the absorption of **BODIPY/Motor** with 505 nm light to that with 395 nm irradiation (Supplementary Section 6). This result implies that the rotation of **BODIPY/Motor** occurs under irradiation with green light, which is beyond the absorption band of bare **Motor**, thereby verifying the availability of the S_1_ for the motor-rotation predicted by theory. Note that mechanisms to trigger the motor-rotation via low-lying triplet excited states reported previously on similar motors^43,67^ and through-space interaction between the two separate molecules can be safely ruled out (Supplementary Section 11). Therefore, the rotation of **BODIPY/Motor** is attributed to synergistic effects in which the moderate π-π interaction between the BODIPY and motor facilitates the absorption in the BODIPY to trigger the rotation in the motor while photoluminescence is also available from the BODIPY.

We also performed circular dichroism (CD) spectroscopy to study chiroptical properties of **BODIPY/Motor** (Fig. 2g–i and Supplementary Section 12). Due to the synergistic effects, the CD spectra of **BODIPY/Motor** at both stable and metastable states clearly show a consecutive chirality transfer from the chiral motor to the achiral BODIPY, i.e., induced CD in the BODIPY. Also, one of the unique features of the molecular motor is that it can serve as a photoresponsive chiral switch, thereby it can invert the sign of the induced CD dynamically upon irradiation (from positive to negative in the case of ***S*-BODIPY/Motor**). This observation opens the prospect to obtain advanced control of the motor-rotation and molecular imaging using circularly polarized light^68,69^.

In summary, we successfully constructed a molecular hybrid of a light-driven MM and a privileged photoluminescent dye. Our design realized both motor-rotation and bright photoluminescence in a single molecule. The magnitude of these very distinct functions can be modulated either using different excitation wavelengths or by change in solvent polarity. Also, the molecular hybrid exhibits beneficial synergistic effects regarding their functionalities, such as green-light-driven rotation and induction of chiroptical properties in the BODIPY. Theoretical calculations revealed a competition between relaxation via PL and internal conversion via a CI to form the metastable isomer of the motor. An excited-state energy barrier height is the key factor determining the competition between these two functionalities, which is itself susceptible to the solvent polarity. Time-resolved PL and TA spectroscopy confirmed the excited-state depopulation of BODIPY when it is attached to the motor, which becomes more efficient in a polar solvent. The present study provides not only unique insight into the fundamental photophysics governing the interplay between the two photoinduced processes but also reveals a strategic molecular design for the development of a new class of light-driven multifunctional hybrid molecules.

## Methods

### Synthesis of BODIPY/Motor

The synthesis of **BODIPY/Motor** was started from a previously reported brominated MM^70^ **M1** as shown in Supplementary Fig. 1. Lithium exchange reaction followed by formylation with dimethylformamide was performed to obtain aldehyde-motor **M2**. Condensation with 2,4-dimethylpyrrole in the presence of acid catalyst afforded dipyrrin-motor **M3**, which acted as a ligand to form a BF_2_ complex, **BODIPY/Motor**. Recrystallization from ether gave orange crystals suitable for single-crystal X-ray diffraction analysis (Fig. 2a, Supplementary Fig. 2 and Supplementary Table 1). The methyl groups at the pyrrole rings provide the perpendicular connection between the BODIPY plane and the naphthalene upper half of the MM with the dihedral angle of 87.6°. The ensuing spatial arrangement suppresses the direct π-system conjugation of the two functional moieties, still maintaining “moderate” interactions between the two moieties to obtain the synergistic effect.

### Time-resolved PL spectroscopy

Time-resolved PL spectroscopy measurements were carried out using a Hamamatsu C5680 streak camera equipped with a Ti:sapphire laser (Mira 900, Coherent) with the central wavelength at 800 nm and the repetition rate of 76 MHz. The excitation wavelength at 400 nm was obtained using a second harmonic generator with the input of the Ti:sapphire laser. Whereas the excitation wavelength at 510 nm was obtained by using a SCG-800 Photonic Crystal Fiber (Newport Corp.) to generate a white light continuum (WLC) fed by the Ti:sapphire laser. A band-pass filter with the central wavelength at 508.5 nm and a full width at half maximum (FWHM) of 10 nm was placed in the WLC beam. For measurements with a time window above 2 ns, the repetition rate was lowered to 2 MHz by a pulse picker. The excitation beam was focused, by a 7.6-cm focal length lens, into a 1-mm quartz cuvette, containing the studied compounds dissolved in acetone or toluene. The polarization of the excitation and PL beams was set at the magic angle ($\sim {54.7}^{{{{{{\rm{o}}}}}}}$). Finally, the PL intensity of the samples was recorded as a function of the wavelength and delay time, producing a PL map.

### Computational details

The Ground-state geometries (S_0min_) of **BODIPY/Motor** stable and metastable isomers were optimized in the gas phase and in a continuum of toluene (ε = 2.38) and acetone (ε = 20.7) according to the Conductor-like Polarizable Continuum Model (C-PCM) scheme^71,72^ at ωB97X-D/cc-pVDZ level of theory^73,74^, including Grimme’s dispersion correction^75^. The vertical excitation energies of the first (S_1_) and second (S_2_) excited states and the corresponding optimized geometries were calculated using SF-TDDFT^76^, employing the B5050LYP functional combined with the cc-pVDZ basis set. All SF-TDDFT calculations were performed in the gas phase, acetone and toluene. Natural transition orbitals (NTOs) of **BODIPY/Motor** stable isomer were calculated in both toluene and acetone using TDDFT(B5050LYP)/cc-pVDZ. Furthermore, the resulting excitation energies were convoluted with Gaussian of suitable full width at half maximum (FWHM) of the corresponding experimental spectrum. Relaxed potential energy surface scans were performed along the ∅_1_ dihedral angle for S_0_ (–170° to –27°) and S_1_ (–170° to –90°) at the SF-TDDFT(B5050LYP)/cc-pVDZ level of theory. Finally, minimum-energy crossing points (MECPs) between S_1_/S_0_ and S_2_/S_1_ were located using the penalty function algorithm^77^ at the SF-TDDFT(B5050LYP)/cc-pVDZ level of theory. All quantum mechanical calculations have been performed using the Q-Chem 5.3 electronic structure program^78^. The geometry parameters, including C_A_-C_B_-C_C_-C_D_ dihedral (∅_1_) and C_E_-C_F_-C_G_-C_H_ dihedral (∅_2_) angles are summarized in Supplementary Table 9.

## Supplementary information

Supplementary Information

## Data Availability

Data that support the findings of this study are available within the paper and its Supplementary Information. The Crystallographic data generated in this study have been deposited in the CCDC database under accession code CCDC 2158217. These data can be obtained free of charge from The Cambridge Crystallographic Data Center via www.ccdc.cam.ac.uk/data_request/cif.
