# Supplementary material for: Synergistic interplay between photoisomerization and photoluminescence in a light-driven rotary molecular motor
Source: Nat Commun. 2022 Sep 30;13:5765. doi: 10.1038/s41467-022-33177-0 (PMC9525625; doi:10.1038/s41467-022-33177-0)
Supplement: Supplementary file 1 — Supplementary Information [file 41467_2022_33177_MOESM1_ESM.pdf]

Supplementary Information for

Synergistic Interplay between Photoisomerization and Photoluminescence in a Light-Driven Rotary Molecular Motor

Ryojun Toyoda[1,2†], Nong V. Hoang[3†], Kiana Gholamjani Moghaddam[3†], Stefano Crespi[1,4], Daisy R. S. Pooler[1], Shirin Faraji[3*], Maxim S. Pshenichnikov[3*], Ben L. Feringa[1,3*]

[1]Stratingh Institute for Chemistry, University of Groningen, Nijenborgh 4, 9747 AG Groningen, the Netherlands

[2] (Present address) Department of Chemistry, Graduate School of Science, Tohoku University, 6-3 Aramaki-Aza-Aoba, Aoba-ku, Sendai, 980-8578, Japan

[3]Zernike Institute for Advanced Materials, University of Groningen, Nijenborgh 4, 9747 AG Groningen, the Netherlands

[4] (Present address) Department of Chemistry -Ångström Laboratory, Uppsala University, Box 523, 751 20 Uppsala, Sweden

†These authors contributed equally to this work

*Correspondence authors. Emails: s.s.faraji@rug.nl (S.F.); m.s.pchenitchnikov@rug.nl (M.S.P.); b.l.feringa@rug.nl (B.L.F.)

Table of Contents

Supplementary Methods

1. Experimental methods and samples 3
   - *1.1. Materials and characterization methods* *3*
   - *1.2. Difference absorption spectroscopy* *3*
   - *1.3. Time-resolved PL spectroscopy* *4*
   - *1.4. Transient absorption spectroscopy* *4*
2. Synthesis and characterization of compounds 6

Supplementary Discussion

3. Single crystal X-ray diffraction 11
4. NMR studies 14
5. Steady-state absorption and PL spectra 17
   - *5.1. Absorption and PL spectra* *17*
   - *5.2. PL quantum yields of BODIPY/Motor* *20*
6. UV/Vis absorption study on photoisomerization and thermal recovery 22
7. Quantum yields of photoisomerization 27
8. Potential energy surface 29
9. Time-resolved PL 34
   - *9.1. PL maps and mean energies* *34*
   - *9.2. PL transients* *37*
   - *9.3. Solvent-polarity dependence on PL transients* *40*
10. Transient absorption (TA) 40
    - *10.1. TA maps and transient spectra* *40*
    - *10.2. TA kinetic traces* *42*
11. Difference absorption (ΔOD) spectra 47
    - *11.1. ΔOD spectra of BODIPY/Motor* *47*
    - *11.2. Is the BODIPY/Motor driven via a triplet pathway under 505-nm excitation?* *48*
    - *11.3. ΔOD spectra of a BODIPY+Motor mixture* *55*
12. Circular dichroism spectroscopy 59
13. NMR spectra for characterization 61

Supplementary references 64

Supplementary Methods

1. Experimental methods and samples

1.1. Materials and characterization methods

All chemicals were purchased from Tokyo Chemical Industry Co. Ltd., Sigma-Aldrich Co. LLC, Thermo Fisher Scientific Inc., Fluorochem Ltd., unless otherwise stated, and they were used without further purification. **Motor**, **BODIPY**, **M1** were prepared according to literature procedures[1–3]. ^{1}H and ^{13}C nuclear magnetic resonance (NMR) data were collected in $CDCl_3$ and recorded on a Varian Mercury-Plus 400 or a Bruker Avance 600 NMR spectrometer at 298 K unless otherwise indicated. Photostationary state (PSS) studies were performed on a Varian Unity Plus 500 NMR spectrometer. Chemical shifts are given in parts per million (ppm) relative to the residual solvent signal. Multiplets in ^{1}H NMR spectra are designated as follows: s (singlet), d (doublet), t (triplet), q (quartet), m (multiplet), br (broad). High resolution mass spectrometry was performed on an LTQ Orbitrap XL spectrometer. Steady-state UV/vis absorption spectra were recorded on an Agilent 8453 UV-vis Diode Array System, equipped with a Quantum Northwest Peltier controller, in 10 mm quartz cuvettes. Irradiation experiments were performed using fiber-coupled light-emitting diodes (LEDs) obtained from Thorlabs Inc. Fluorescence spectra were collected with a JASCO FP-6200 spectrometer. Separation of the enantiomers was performed with Thar SFC system with PDA detector using Chiralpak IA (5 μm, 4.6x250mm, 10% methanol in supercritical CO_2, 4 ml/min). Circular dichroism (CD) spectra were recorded on a JASCO 810 spectropolarimeter.

1.2. Difference absorption spectroscopy

Difference absorption spectroscopy measurements were performed using an UV/Vis/NIR spectrometer (Lambda 900) and two different types of the light source for irradiation in the green or red wavelength region. The green irradiation at 505 nm was provided by a fiber coupled LED (M505F3, Thorlabs) with the maximum power of ~26 mW. The red irradiation at 635 nm was implemented using a 635-nm diode laser (Lasermate Group, Inc.) with a maximum power of 550 mW. The output light source irradiated a 1-cm quartz cuvette, containing a 2-mL solution of the studied compounds dissolved in toluene. For experiments of **BODIPY/Motor** mixed with **PtTPBP** or **PdPc(OBu)$_8$**, all samples were degassed in an argon environment for at least 10 min before the measurements. The irradiation time was fixed for 1 min or 5 min, which depends on specific measurements, and the absorption of the samples was recorded before and 10 secs right after irradiation (from 10 secs up to ~30 min, for time-evolution experiments); all in the dark. Finally, difference absorption (ΔOD) spectra were obtained by subtracting the absorption spectrum before irradiation from the ones after irradiation. As the ΔOD spectra are

extremely sensitive to temperature, the room temperature was maintained at $\sim 23 \pm 1$ °C for all measurements.

1.3. Time-resolved PL spectroscopy

Time-resolved PL spectroscopy measurements were carried out using a Hamamatsu C5680 streak camera equipped with a Ti:sapphire laser (Mira 900, Coherent) with a central wavelength at 800 nm and repetition rate of 76 MHz. The excitation wavelength at 400 nm was obtained using a second harmonic generator with the input of the Ti:sapphire laser. In contrast, the excitation wavelength at 510 nm was obtained by using a SCG-800 Photonic Crystal Fiber (Newport Corp.) to generate a white light continuum (WLC) fed by the Ti:sapphire laser. A band-pass filter with the central wavelength at 508.5 nm and a full width at half maximum (FWHM) of 10 nm was placed in the WLC beam. For measurements with a time window above 2 ns, the repetition rate was lowered to 2 MHz by a pulse picker. The excitation beam was focused, by a 7.6-cm focal length lens, into a 1-mm quartz cuvette, containing the studied compounds dissolved in either isopropanol, acetonitrile, acetone or toluene. The apparatus functions of the setup were $\sim$5 ps and $\sim$10 ps (standard deviation of a Gaussian function) for the excitation wavelengths of 400 nm and 510 nm, respectively. The PL signal was collected at a $\sim 90^{o}$ angle with respect to the excitation laser beam. The polarization of the excitation and PL beams was set at the magic angle ($\sim 54.7^{o}$). Long-pass filters (Thorlabs) with the cut-off wavelength of 420 nm and 515 nm were used to remove the stray light of the excitation beam for 400 nm and 510 nm excitations, respectively, to the polychromator entrance slit. Finally, the PL intensity of the samples was recorded as a function of the wavelength and delay time, producing a PL map. In all measurements, the room temperature was kept at 20 ^{o}C.

1.4. Transient absorption spectroscopy

The setup of transient absorption (TA) spectroscopy was based on a pump–probe configuration described previously[4]. In brief, an amplified mode-locked Ti:sapphire laser (Legend Elite Duo, Coherent) centered at 800 nm (1 kHz repetition rate) was used. The laser output was split into pump (~90%) and probe (~10%) beams. To obtain the excitation wavelength of 400 nm, the pump beam was frequency-doubled by using a β-barium borate (BBO) crystal. A mechanical translation stage (LS-180, Physik Instrument) with 508 mm excursion was used to delay the probe pulse with respect to the pump pulse. The probe beam was focused into a 2-mm sapphire crystal to generate a white-light (400–850 nm) continuum (WLC). A short-pass filter with a cut-off wavelength of 750 nm placed after the sapphire crystal was used to remove residual fundamental frequency radiation from the WLC. The polarization of the pump and probe beams

was linear and set at the magic angle (54.7^{o}). Both the pump and the probe beams were focused and spatially overlapped in a 0.2-mm flow cell (Starna Scientific Ltd.), connected to a peristaltic pump (Masterflex, Cole-Parmer) to refresh the sample in the excitation spot. Then, TA of the probe beam in the flow cell was recorded using two different types of detectors, a 350–700 nm compact spectrometer (CCS100/M, Thorlabs) and a silicon photodiode (DET36A, Thorlabs) amplified by a lock-in amplifier (SR830 DSP, Stanford Research Systems). The spectrometer detected the TA spectra in the range of 460–700 nm; however, it had a lower signal-to-noise ratio as compared to the lock-in referenced photodiode. The photodiode detector detected TA signals at a particular probe wavelength using a band-pass filter (e.g., 510 nm or 550 nm, FWHM = 10 nm for both) placed in front of the photodiode. This arrangement improved the sensitivity down to $\mathit{\Delta OD} \cong 10^{-5}$. Further details of data acquisition and corrections are described in Reference 4.

2. Synthesis and characterization of compounds

9-(2-methyl-2,3-dihydro-1*H*-cyclopenta[*a*]naphthalen-1-ylidene)-9*H*-fluorene: **Motor**

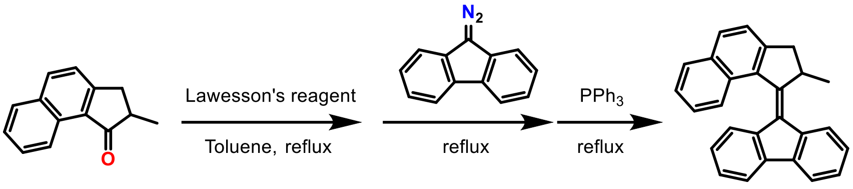

The synthesis of **Motor** was conducted following a previously report procedure[1]. To a toluene solution of 2-methyl-2,3-dihydro-1*H*-cyclopenta[*a*]naphthalen-1-one (668.1 mg, 3.51 mmol, 50 mL), Lawesson's reagent (4.17 g, 10.3 mmol) was added and the mixture was heated at reflux for 3 h. The mixture was cooled down and quickly purified by column chromatography (Silica, pentane : ethyl acetate = 10 : 1). The blue band was collected, concentrated in vacuo, and dissolved in toluene (30 mL). It was mixed with 9-diazo-9*H*-fluorene (1.75 g, 9.1 mmol) and refluxed overnight. Then triphenylphosphine (1.83 g, 7.0 mmol) was added and stirred overnight. The resultant mixture was purified by column chromatography (Silica, pentane) to afford **Motor** as a yellow powder.

9-(5-bromo-2-methyl-: **BODIPY**

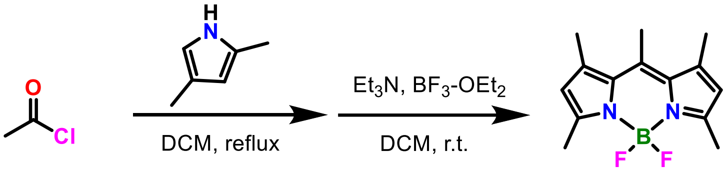

The synthesis of **BODIPY** was conducted following a previously report procedure[2]. To a dichloromethane solution of 2,4-dimethylpyrrole (1.08 mL, 10 mmol), acyl chloride (1.8 mL, 2.5 mmol) was added dropwise over 20 min. The mixture was heated to reflux for 1 h. After cooling to room temperature, the volatiles were removed in vacuo and the resulting crude was dissolved in dichloromethane (100 mL). To the solution, triethylamine (7.5 mL) and then BF_3-OEt_2 (5.7 mL) were added, and the solution was stirred overnight at room temperature. The solvent was removed under reduced pressure and the product was purified by column chromatography (Silica, hexane to hexane : DCM = 1 : 1) and reprecipitation from DCM/hexane to afford **BODIPY** as an orange powder.

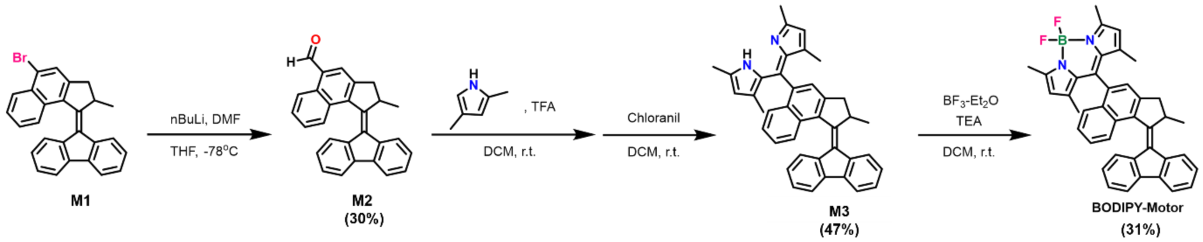

Supplementary Fig. 1. Synthetic scheme of **BODIPY/Motor**.

9-(5-bromo-2-methyl-2,3-dihydro-1*H*-cyclopenta[*a*]naphthalen-1-ylidene)-9*H*-fluorene: **M1**

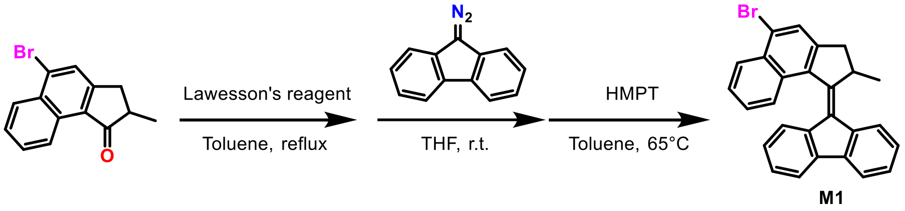

The synthesis of **M1** was conducted following a previously report procedure[3]. To a toluene solution of 5-bromo-2-methyl-2,3-dihydro-1*H*-cyclopenta[*a*]naphthalen-1-one (1.48 g, 5.38 mmol, 60 mL), Lawesson's reagent (4.67 g, 11.5 mmol) was added and the mixture was heated at reflux for 3 h. The mixture was cooled down and quickly purified by column chromatography (Silica, pentane : ethyl acetate = 10 : 1). The blue band was collected, concentrated in vacuo, and dissolved in THF (60 mL). It was mixed with 9-diazo-9*H*-fluorene (2.03 g, 9.1 mmol) in THF (90 mL) and stirred at room temperature overnight. Then the solvent was removed under reduced pressure and the resulting solid was dissolved in toluene (60 mL). HMPT (2.3 mL, 12.6 mmol) was added to the solution and the mixture was heated at 65 °C overnight. The resultant mixture was purified by column chromatography (Silica, pentane) to afford **M1** as a yellow powder.

1-(9*H*-fluoren-9-ylidene)-2-methyl-2,3-dihydro-1*H*-cyclopenta[*a*]naphthalene-5-carbaldehyde: **M2**

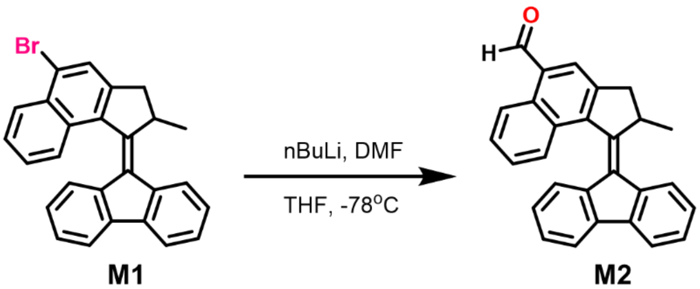

To a tetrahydrofuran (THF) solution of **M1** (568.8 mg, 1.34 mmol, 1 eq in 30 mL), n-BuLi solution (1.6 M in hexane, 1.0 mL, 1.6 mmol, 1.2 eq) was added dropwise at -78C under N_2 atmosphere. After stirring for 30 min, dimethylformaldehyde (200 μL, 2.58 mmol, 1.9 eq) was added. The mixture was allowed to warm to room temperature overnight, quenched with 1 M HCl aqueous solution and extracted with ether. The organic layer was dried over Na_2SO_4 and the solvent was removed under reduced pressure. Column chromatography (Silica, DCM: pentane = 1:2 to 1:1) gave **M2** as a yellow powder (152.6 mg, 30%).

^{1}H NMR (600 MHz, Chloroform-*d*) δ 10.54 (s, 1H), 9.31 (d, *J* = 8.6 Hz, 1H), 8.12 (s, 1H), 8.01 – 7.96 (m, 2H), 7.87 – 7.83 (m, 1H), 7.75 (d, *J* = 7.5 Hz, 1H), 7.68 (ddd, *J* = 8.4, 6.8, 1.2 Hz, 1H), 7.47 – 7.40 (m, 3H), 7.26 – 7.22 (m, 1H), 6.81 – 6.76 (m, 1H), 6.66 (d, *J* = 7.9 Hz, 1H), 4.44 – 4.38 (m, 1H), 3.67 (dd, *J* = 15.0, 5.7 Hz, 1H), 2.88 (d, *J* = 15.0 Hz, 1H), 1.43 (d, *J* = 6.8 Hz, 3H); ^{13}C NMR (151 MHz, $CDCl_3$) δ 193.04, 149.06, 145.31, 143.61, 140.75, 140.23, 139.59, 136.89, 133.96, 133.33, 132.57, 130.34, 130.26, 128.44, 128.07, 128.03, 127.95, 127.44, 127.39, 126.34, 126.11, 125.45, 124.64, 120.00, 119.33, 45.56, 41.64, 19.25; HRMS (APCI pos) *m/z* calcd for $C_{28}H_{21}O$ $[M+H]^+$ 373.15869, found 373.15897.

2-((1-(9*H*-fluoren-9-ylidene)-2-methyl-2,3-dihydro-1*H*-cyclopenta[*a*]naphthalen-5-yl)(3,5-dimethyl-2*H*-pyrrol-2-ylidene)methyl)-3,5-dimethyl-1H-pyrrole: **M3**

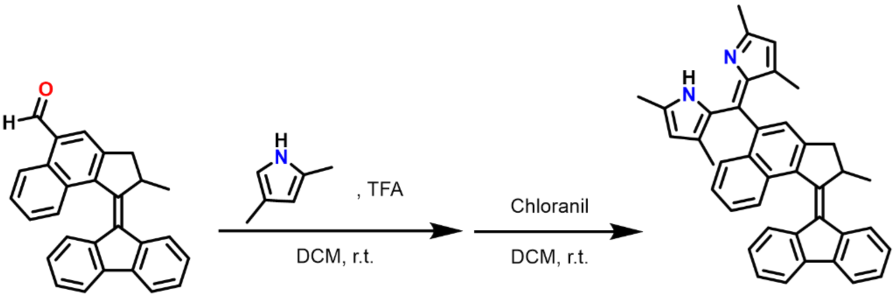

Under N_2 atmosphere, 2,4-dimethylpyrrole (100 μL, 971 μmol, 3.8 eq) and trifluoroacetic acid (5 μL) was added to a DCM solution of **M2** (95.7 mg, 254 μmol, 1eq in 10 mL). The mixture was stirred overnight at room temperature in dark. After completion of the starting material, chloranil (69.9 mg, 284 μmol, 1.1 eq) was added and the mixture was stirred for 30 min. The solvent was removed in vacuo and the crude product was purified by column chromatography (Alumina, DCM: pentane = 1:2 to 1:0) to give **M3** as an orange powder (64.7 mg, 47%).

^1H NMR (600 MHz, Methylene Chloride-d_2) δ 8.04 – 7.99 (m, 1H), 7.90 (d, J = 8.3 Hz, 1H), 7.88 – 7.84 (m, 1H), 7.79 (dd, J = 8.5 Hz, 2H), 7.54 (s, 1H), 7.44 – 7.39 (m, 2H), 7.38 (ddd, J = 8.3, 6.8, 1.2 Hz, 1H), 7.31 (ddd, J = 8.2, 6.7, 1.2 Hz, 1H), 7.22 (ddd, J = 7.4, 0.9 Hz, 1H), 6.82 – 6.76 (m, 1H), 6.70 (d, J = 7.9 Hz, 1H), 5.89 (s, 1H), 5.88 (s, 1H), 4.38 (m, 1H), 3.62 (dd, J = 15.1, 5.6 Hz, 1H), 2.79 (d, J = 15.2 Hz, 1H), 2.39 (s, 3H), 2.38 (s, 3H), 1.41 (d, J = 6.7 Hz, 3H), 1.14 (s, 3H), 1.07 (s, 3H); ^{13}C NMR (151 MHz, CD_2Cl_2) δ 152.60, 152.05, 151.55, 147.67, 140.58, 140.51, 140.26, 140.25, 140.10, 138.83, 137.71, 137.60, 137.43, 137.18, 136.98, 132.61, 131.20, 130.61, 127.89, 127.62, 127.60, 127.49, 127.28, 126.60, 126.56, 126.49, 126.17, 125.76, 124.73, 120.31, 120.19, 119.89, 119.59, 46.18, 42.45, 19.67, 16.41, 16.32, 14.61, 14.48; HRMS (APCI pos) m/z calcd for $C_{40}H_{35}N_2$ $[M+H]^+$ 543.27948, found 543.27969.

(*Z*)-2-((1-(9*H*-fluoren-9-ylidene)-2-methyl-2,3-dihydro-1*H*-cyclopenta[*a*]naphthalen-5-yl)(3,5-dimethyl-2*H*-pyrrol-2-ylidene)methyl)-1-(difluoro(methyl)-λ^4-boraneyl)-3,5-dimethyl-1*H*-pyrrole: **BODIPY/Motor**

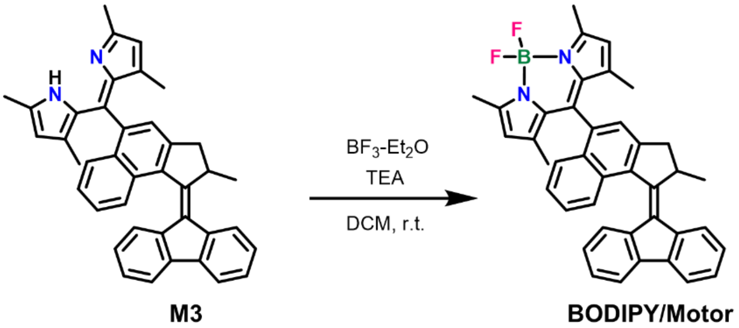

M3 (37.2 mg, 68 μmol, 1 eq) was dissolved in DCM (7 mL) under N_2 atmosphere. To this solution, triethylamine (100 μL) was added and the mixture was stirred at room temperature for 30 min. Then BF_3-Et_2O (90 μL) was added followed by stirring overnight. After removal of the solvent, the residue was purified by column chromatography (Silica, DCM: pentane = 1:1). A green-emissive band was collected, the solvent was evaporated, and the residue was recrystallized from ether to give **BODIPY/Motor** as an orange crystal (12.5 mg, 31%).

^{1}H NMR (600 MHz, Methylene Chloride-*d*$_2$) δ 8.04 – 7.99 (m, 1H), 7.90 – 7.85 (m, 2H), 7.83 (d, *J* = 8.4 Hz, 1H), 7.78 (d, *J* = 7.5 Hz, 1H), 7.57 (s, 1H), 7.45 – 7.40 (m, 3H), 7.35 (dd, *J* = 7.1 Hz, 1H), 7.23 (dd, *J* = 7.3 Hz, 1H), 6.79 (dd, *J* = 8.0 Hz, 1H), 6.66 (d, *J* = 7.9 Hz, 1H), 6.04 (s, 1H), 6.03 (s, 1H), 4.46 – 4.36 (m, 1H), 3.64 (dd, *J* = 15.3, 5.6 Hz, 1H), 2.81 (d, *J* = 15.2 Hz, 1H), 2.58 (s, 4H), 2.57 (s, 7H), 1.41 (d, *J* = 6.7 Hz, 3H), 1.27 (s, 3H), 1.20 (s, 3H); ^{13}C NMR (151 MHz, CD_2Cl_2) δ 156.38, 156.31, 150.90, 147.65, 143.65, 143.49, 140.91, 140.61, 140.40, 140.17, 138.38, 137.61, 135.28, 132.45, 132.41, 131.81, 131.48, 130.66, 128.11, 127.82, 127.72, 127.71, 127.15, 126.53, 126.10, 126.08, 124.85, 124.82, 121.86, 121.71, 120.25, 119.68, 46.23, 42.44, 19.57, 14.98, 14.46, 14.28; HRMS (APCI pos) *m/z* calcd for $C_{40}H_{34}BF_2N_2$ $[M+H]^+$ 591.27776, found 591.27619.

Supplementary Discussion

3. Single crystal X-ray diffraction

A single crystal of **BODIPY/Motor** was grown in diethylether and mounted on a cryoloop and placed in the nitrogen stream (Temperature, $T \sim 150\,\mathrm{K}$) of a Bruker-AXS D8 Venture diffractometer. Data collection and processing was carried out using the Bruker APEX3 software[5]. A multi-scan absorption correction was applied, based on the intensities of symmetry-related reflections measured at different angular settings (SADABS)[6]. The structure was solved using SHELXT[7] and refinement was performed using SHELXL[8]. The hydrogen atoms were generated by geometrical considerations, constrained by idealized geometries and allowed to ride on their carrier atoms with an isotropic displacement parameter related to the equivalent displacement parameter of their carrier atoms. No A- or B-level alerts were raised by CheckCIF for the fully refined structure.

Supplementary Table 1. Crystal data and structure refinement for **BODIPY/Motor** (CCDC 2158217).

Identification code	cu_RTG34again_0m_a
Empirical formula	$C_{40}H_{33}BF_2N_2$
Formula weight	590.49
Temperature/K	150.0
Crystal system	monoclinic
Space group	$P2_1/n$
a/Å	10.8025(11)
b/Å	17.729(2)
c/Å	16.8747(18)
α/°	90
β/°	107.631(6)
γ/°	90
Volume/Å^3	3080.0(6)
Z	4
ρ_{calc}/g/cm^3	1.273
μ/mm^{-1}	0.649
F(000)	1240.0
Crystal size/mm^3	0.81 × 0.207 × 0.194
Radiation	CuKα (λ = 1.54184)
2Θ range for data collection/°	7.422 to 137.152
Index ranges	$-13 \le h \le 12, -21 \le k \le 21, -20 \le l \le 20$
Reflections collected	100144
Independent reflections	5664 [R_{int} = 0.1684, R_{sigma} = 0.0521]
Data/restraints/parameters	5664/0/412
Goodness-of-fit on F^2	1.023
Final R indexes [I>=2σ (I)]	R_1 = 0.0651, wR_2 = 0.1696
Final R indexes [all data]	R_1 = 0.0896, wR_2 = 0.1943
Largest diff. peak/hole / e Å^{-3}	0.43/-0.44

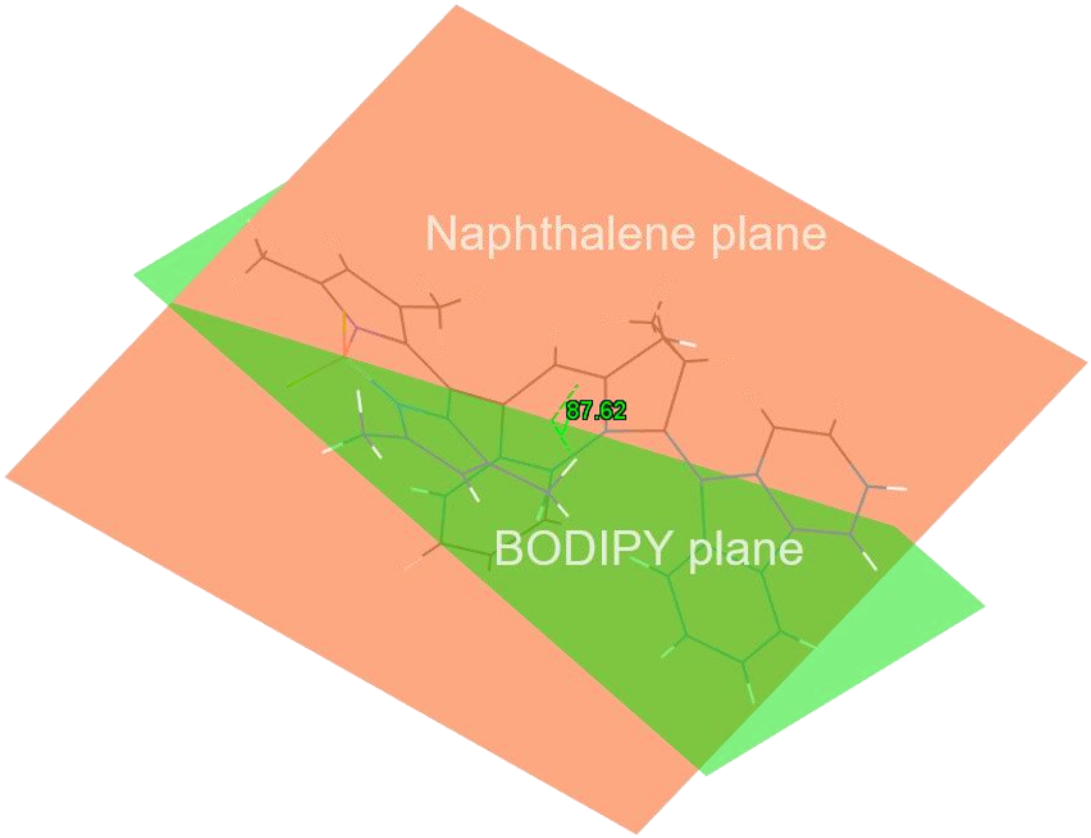

Supplementary Fig. 2. Dihedral angle between the BODIPY plane and the upper half of the motor part in the crystal structure of **BODIPY/Motor**.

4. NMR studies

A solution of **BODIPY/Motor** was prepared and transferred into an NMR tube which was subsequently fitted with a glass fibre or in situ irradiation. The sample was placed in a Varian Unity Plus 500 NMR spectrometer and cooled to –40 °C. ^{1}H NMR spectra were recorded before irradiation and after irradiation to PSS using an appropriate LED. Ratios of stable:metastable isomers at PSS were determined by comparing the integrals of the two signals from the related proton. The sample was then warmed to room temperature to allow for complete thermal helix inversion (THI) before recording another ^{1}H NMR spectrum at –40 °C.

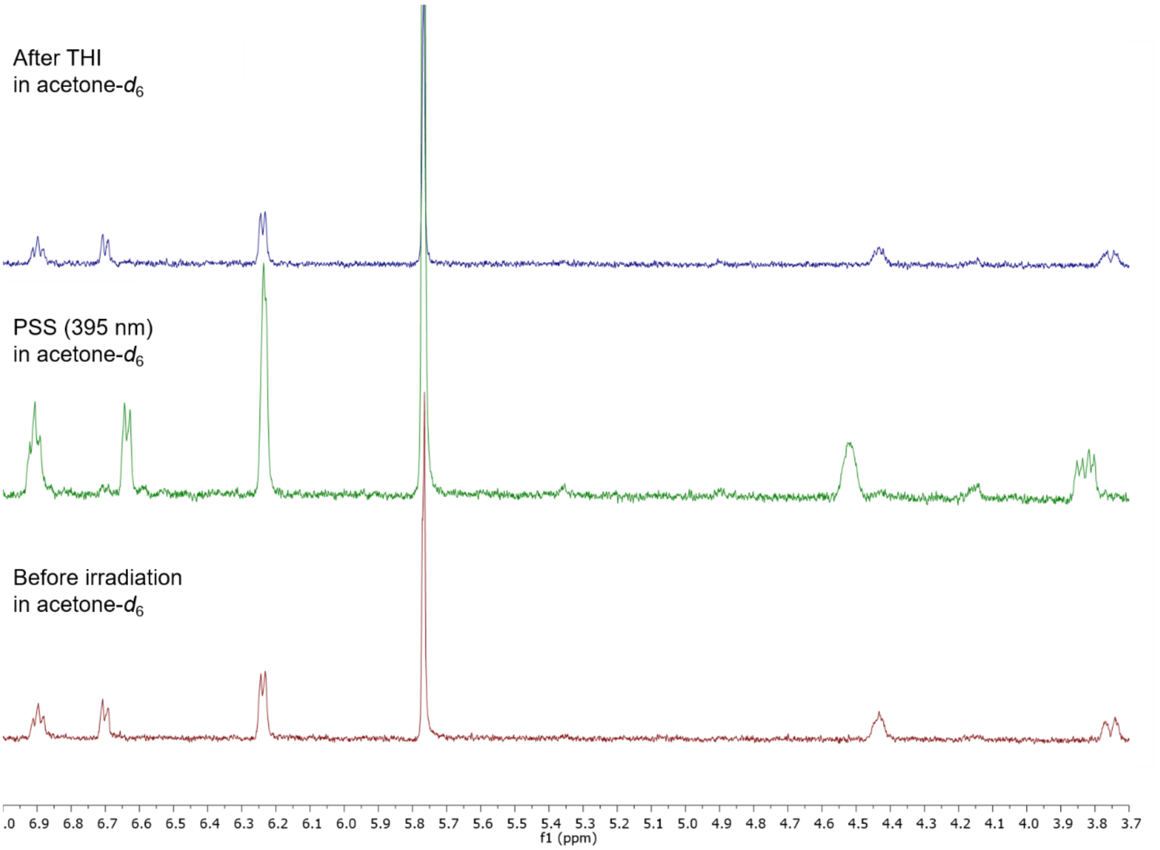

Supplementary Fig. 3. Stack of ^{1}H NMR spectra of **BODIPY/Motor** before irradiation with a 395 nm LED, at PSS and after completed THI measured in acetone-d_6 at –40 °C.

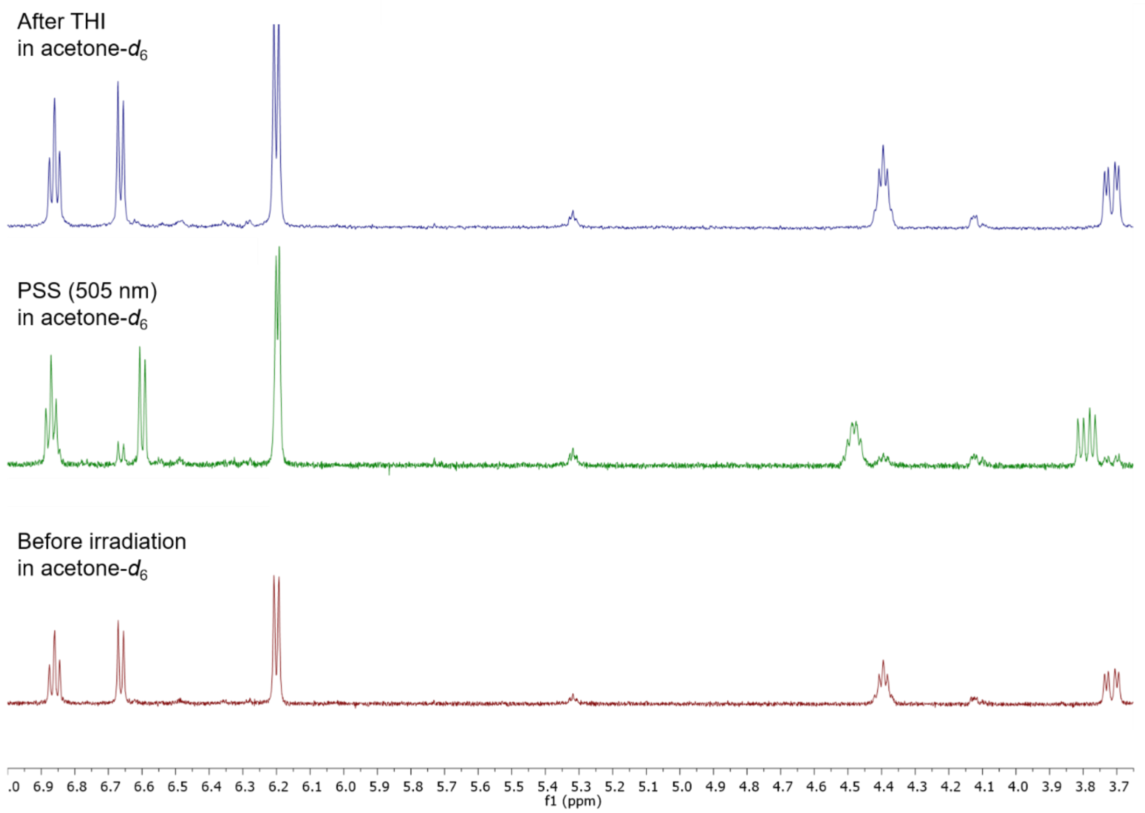

Supplementary Fig. 4. Stack of ^{1}H NMR spectra of **BODIPY/Motor** before irradiation with a 505 nm LED, at PSS and after completed THI measured in acetone-d_6 at –40 °C.

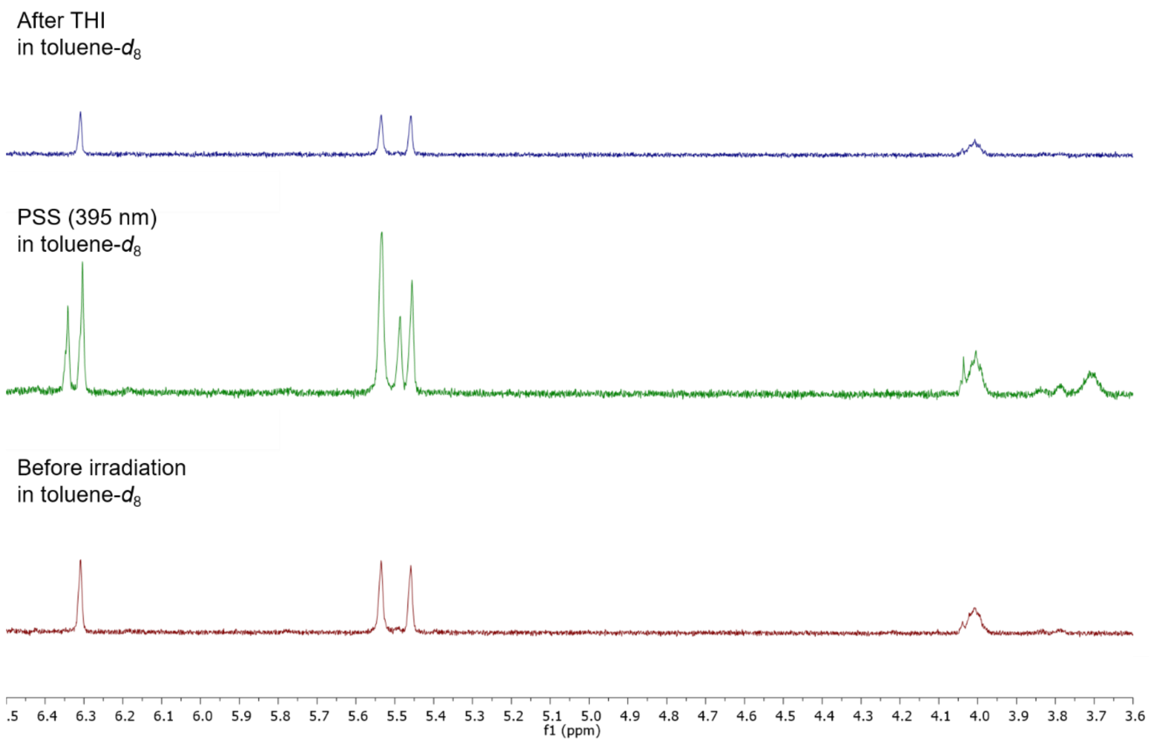

Supplementary Fig. 5. Stack of ^{1}H NMR spectra of **BODIPY/Motor** before irradiation with a 395 nm LED, at PSS and after completed THI measured in toluene-d_8 at –40 °C.

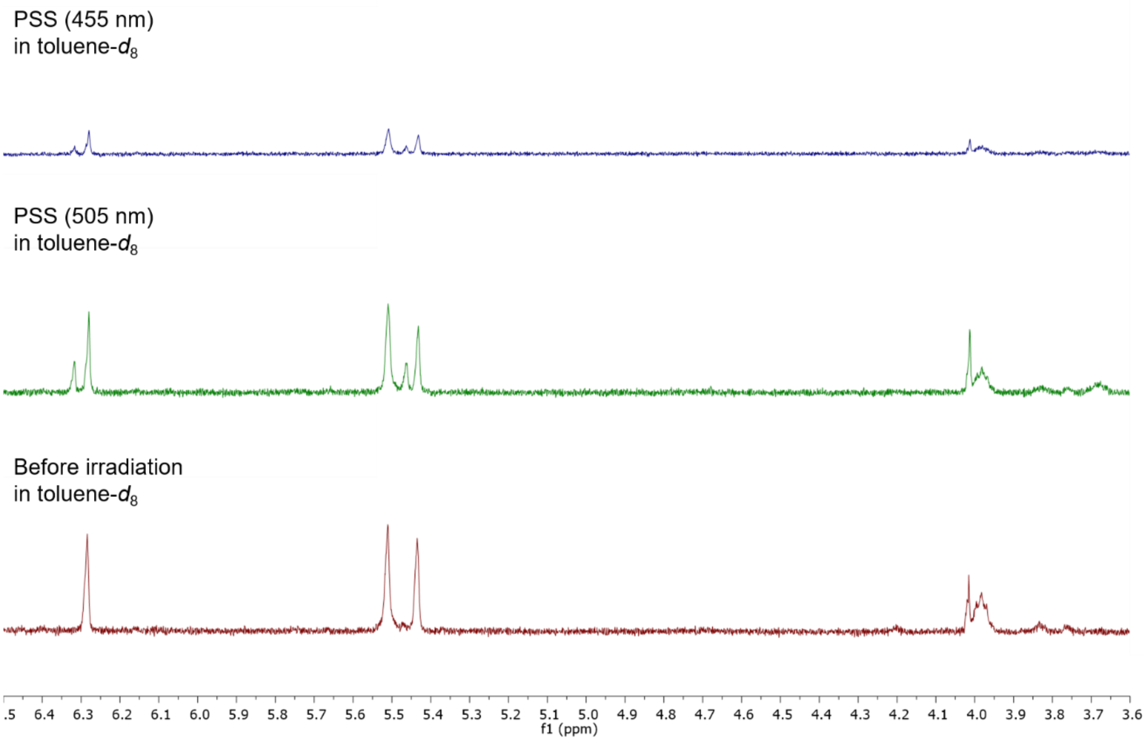

Supplementary Fig. 6. Stack of ^{1}H NMR spectra of **BODIPY/Motor** before irradiation with a 505 nm LED, at PSS with 505 nm LED and 455 nm LED measured in toluene-d_8 at –40 °C.

Supplementary Table 2. Summary of ratios of stable and metastable isomers of **BODIPY/Motor** at PSS in toluene and acetone at –40 °C.

Wavelength	Solvent	Stable:Metastable
395 nm	Toluene	60:40
505 nm	Toluene	70:30
395 nm	Acetone	4:96
505 nm	Acetone	15:85

5. Steady-state absorption and PL spectra

5.1. Absorption and PL spectra

Supplementary Fig.7a,b shows absorption and PL spectra of bare **BODIPY**, BODIPY attached to motor (**BODIPY/Motor**) and a mixture of bare **BODIPY** and bare **Motor** (herein, denoted as **BODIPY+Motor**) in toluene and acetone.

For bare **BODIPY**, the shapes of the absorption and PL spectra of bare **BODIPY** in toluene are quite similar to those in acetone, which exhibit a strong absorption band with a maximum at ~500 nm and a PL band between 500 nm and 600 nm. The spectra reported herein are in line with the previous studies[9]. There is a small blueshift in the absorption and PL spectra, amount to ~7 nm, when changing the solvent from toluene to acetone. This shift is attributed to solvent polarity effects, at which the absorption and PL spectra of **BODIPY** are blue-shifted with increasing the solvent polarity[9–11]. The PL spectra of **BODIPY+Motor** in toluene and acetone are similar to those of bare **BODIPY**, indicating that PL of **BODIPY+Motor** originates only from BODIPY.

For **BODIPY/Motor**, the absorption spectra of **BODIPY/Motor** in toluene and acetone (Supplementary Fig. 7a,b; *green solid lines*) consist of two main absorption bands which have the contributions of the BODIPY moiety (peak at ~500 nm) and the motor core moiety (peak at ~390 nm). However, there is a redshift, amounting to ~10 nm, in the absorption spectrum of **BODIPY/Motor** as compared to that of bare **BODIPY**. This small redshift indicates a weak electronic coupling between the BODIPY moiety and the motor core moiety. The PL spectra of **BODIPY/Motor** in toluene and acetone at 400 nm excitation (Supplementary Fig. 7a,b; *green dashed lines*) are similar in shape to those at 480 nm (Supplementary Fig. 7a,b; *dot curves*), indicating that PL originates from the same excited state. Therefore, we conclude that there is no excitation-wavelength dependence on the PL spectral shape of **BODIPY/Motor**. It is also noticeable that the PL spectra of **BODIPY/Motor** in acetone show a long tail up to 700 nm. However, time-resolved PL data (Supplementary Fig. 19) shows that PL in the tail (600–700 nm) decays similarly to that in the strong PL band (500–600 nm) and therefore PL in both regions originates from the same excited state.

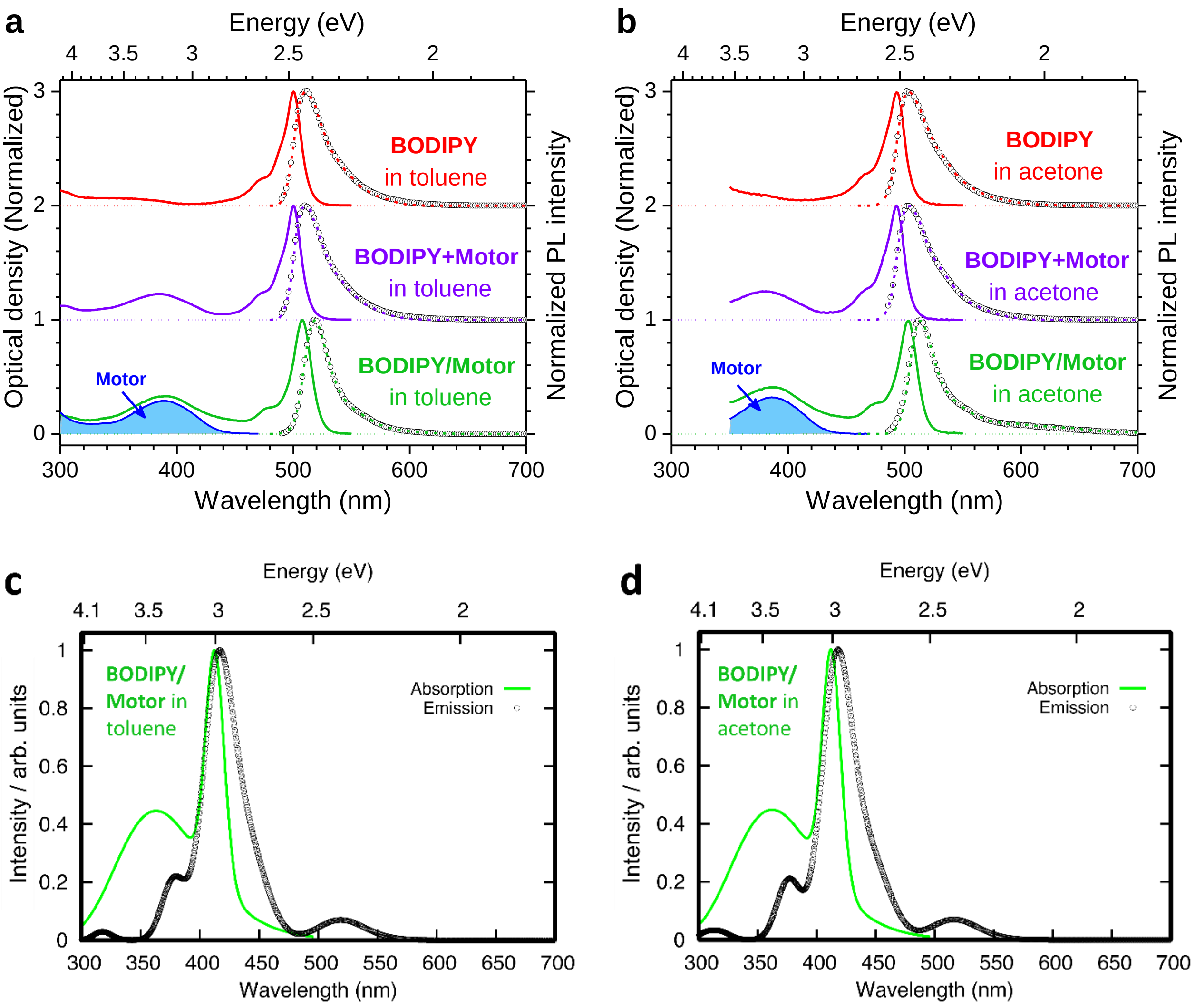

Supplementary Fig. 7. (**a**–**b**) Experimental absorption (solid lines) and PL (dashed lines and dots) spectra of bare **BODIPY** (red), **BODIPY+Motor** (violet) and **BODIPY/Motor** (green) in toluene (**a**) and acetone (**b**). The excitation wavelengths of PL spectra were 400 nm and 480 nm (for dashed lines and dots, respectively). The blue shaded areas show absorption spectra of the bare **Motor** in toluene or acetone as reference spectra. For the sake of clarity, the spectra for **BODIPY** and **BODIPY+Motor** are offset by 2 and 1, respectively. (**c**–**d**) Calculated absorption (solid lines) and emission (dot curves) spectra of **BODIPY/Motor** in toluene (**c**) and acetone (**d**).

Supplementary Table 3. Vertical excitation energies of S_1 and S_2 states for **BODIPY/Motor** stable and metastable isomers using SF-TDDFT(B5050LYP)/cc-pVDZ.

Isomer	Excitation energy, eV /nm (osc. strength)							
	Gas phase			Toluene			Acetone	
	S_1	S_2		S_1	S_2		S_1	S_2
Stable	3.00/413 (0.664)	3.40/365 (0.389)		2.99/415 (0.651)	3.40/365 (0.400)		2.99/415 (0.631)	3.40/365 (0.416)
Metastable	3.00/413 (0.658)	3.39/366 (0.335)		2.99/415 (0.643)	3.40/365 (0.368)		2.99/415 (0.621)	3.39/366 (0.383)

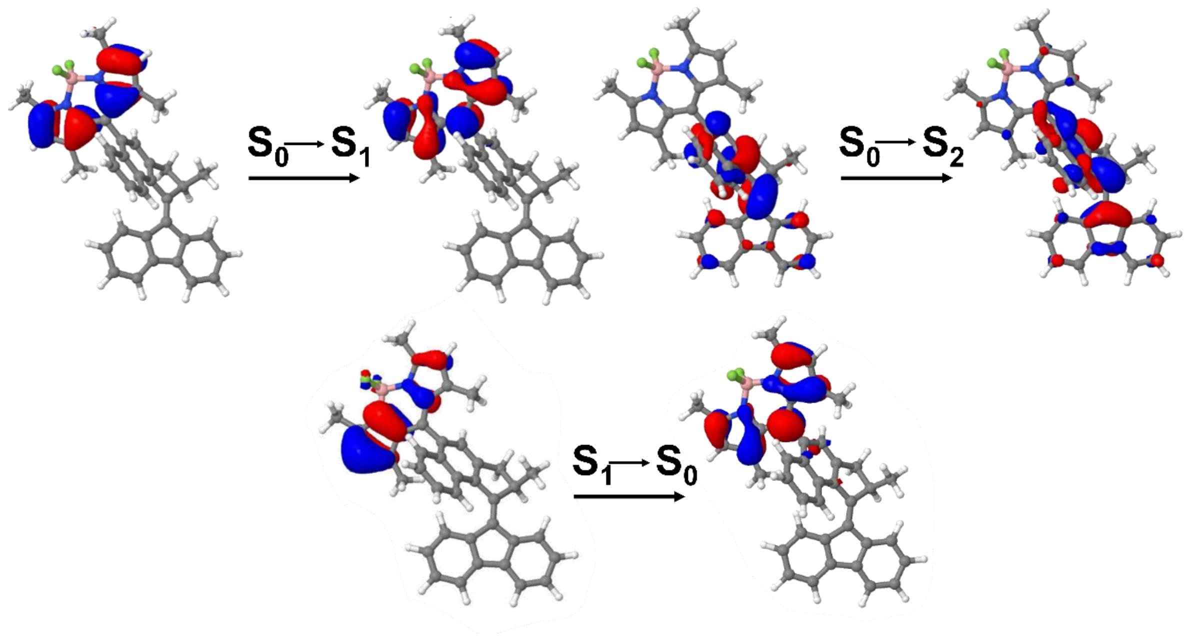

Supplementary Fig. 8. Natural transition orbitals (NTOs) of the **BODIPY/Motor** stable isomer for absorption and emission in toluene.

5.2. PL quantum yields of BODIPY/Motor

Steady-state PL spectra of **BODIPY/Motor** were recorded in toluene, THF and acetone (Supplementary Fig. 9). The absorbance of all samples did not exceed 0.1 so that PL reabsorption effect can be minimized. After keeping the samples in the dark for at least 30 min to ensure all metastable isomers proceeded to stable isomers, the PL spectra for **BODIPY/Motor** in different solvents was measured (Supplementary Fig. 9; blue lines). Then, the samples were irradiated with a 395 nm LED for ~1 min. After the PSS was reached, the samples were transferred to the PL spectrometer within 10 secs and the PL spectra were recorded (Supplementary Fig. 9; orange lines). The PL spectra of **BODIPY/Motor** after reaching the PSS show the lower PL intensity (at least two folds) than those before irradiation. Relative photoluminescence quantum yields (PLQYs) were determined following the procedure described in the previous literature[12]. More than five samples with different absorbance were prepared and their PL spectra were obtained before and 10 second right after irradiation with LED light in each solvent. The peak area for each spectrum was calculated and plotted against absorbance. The slope of the linear fit of the plots was compared with that of a standard solution (fluorescein, 0.1 M in NaOH, a PLQY of φ_{st}~92%)[13]. The relative PLQYs of a sample was calculated by using the following equation:

$$\varphi_x = \frac{m}{m_{st}} \times \frac{n^2}{n_{st}^2} \times \varphi_{st} \qquad (1)$$

where the subscripts *x* and *st* represent sample and standard. φ, *m*, and *n* denote relative PLQY, slope from the plot, and refractive index of the solvent, respectively. Supplementary Table 4 summarized PLQYs of **BODIPY/Motor** in toluene, THF and acetone before and 10 s right after irradiation.

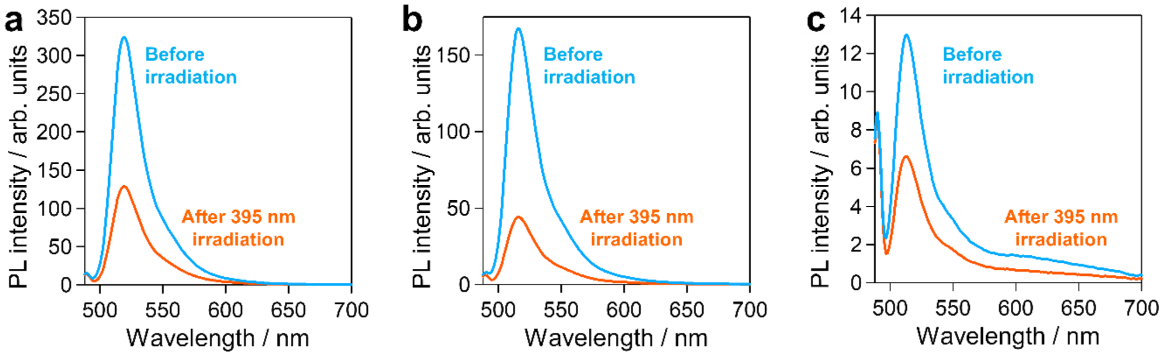

Supplementary Fig. 9. Steady-state PL spectra of **BODIPY/Motor** in toluene (**a**), THF (**b**) and acetone (**c**) before and 10 s right after irradiation with a 395 nm LED at 5 °C for 1 min. The excitation wavelengths of all samples were 488 nm.

Supplementary Table 4. Summary of photoluminescence quantum yields (PLQYs; using Equation (1)) of **BODIPY/Motor** in toluene, THF and acetone before and 10 s right after 395 nm irradiation at 5 °C. All samples were excited at a 488 nm wavelength. Fluorescein (PLQY ~ 92%) was used for a reference sample.

	Toluene	THF	Acetone
Before irradiation	54%	23%	2.6%
10 s right after irradiation	19%	5.3%	1.0%

6. UV/Vis absorption studies on photoisomerization and thermal recovery

The motor rotation of **BODIPY/Motor** under irradiation condition was studied using UV/Vis absorption spectroscopy and LED light sources. Solution samples were prepared by dissolving **BODIPY/Motor** in different solvents of either toluene, THF or acetone. To ensure all the metastable isomers proceeded to the stable isomers, all samples were kept in the dark for at least 30 min before the measurement. Absorption spectra of the samples were measured during and after irradiation with a 395 nm LED. Upon 395 nm irradiation, the absorption spectra of **BODIPY/Motor** in different solvents (Supplementary Fig. 10a, 11a and 12a) shows a similar behaviour which is a clean isosbestic point at ~415 nm and the decrease (increase) in absorption band centred at ~390 nm (~450 nm). This result indicates that the photoisomerization process is selective, leading to the formation of the metastable isomer. After 30 secs of irradiation, the spectral change was no longer observed, indicating that a PSS was obtained. After removal of the LED light source and keeping the samples in the dark, the original spectra of the **BODIPY/Motor** stable isomer in different solvents (Supplementary Fig. 10c, 11c and 12c) were completely recovered within one hour. This recovery in the absorption spectra indicates that the metastable isomers proceeded to the second, identical stable isomers via undergoing THI. These photoisomerization and THI processes were repeated at least four times without significant signs of fatigue (Supplementary Fig. 10d, 11d and 12d), demonstrating the high photostability of **BODIPY/Motor** under ultraviolet (UV) light and its robust switching behaviours.

In parallel to the 395 nm irradiation, **BODIPY/Motor** in different solvents exhibits a similar response when using the irradiation light at 505 nm, which lies in the absorption band of the BODIPY moiety (Supplementary Fig. 7a,b). Further investigations on **BODIPY/Motor** under 505 nm irradiation are provided in Supplementary Section 11.

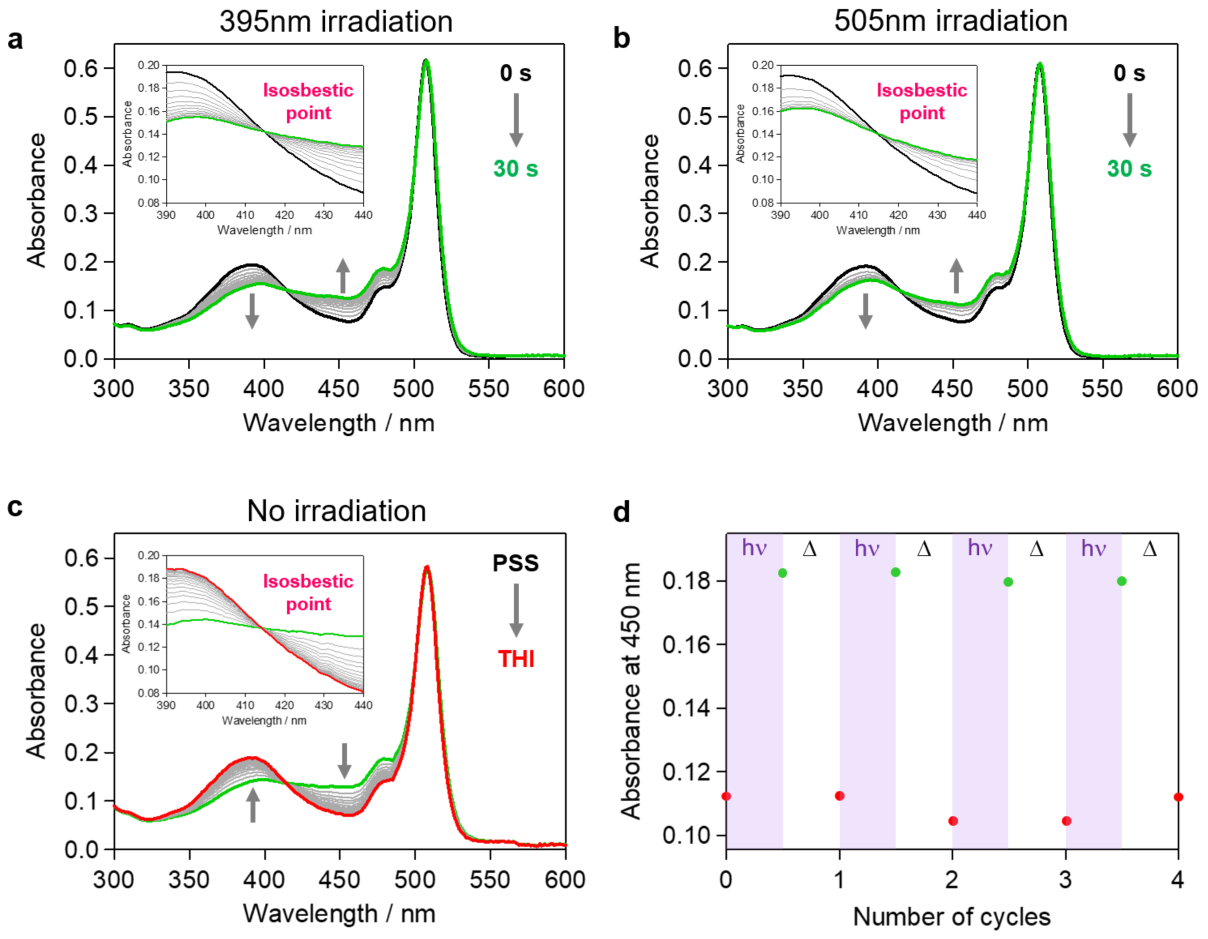

Supplementary Fig. 10. (**a**–**b**) UV/Vis spectral change of **BODIPY/Motor** in toluene at 5 °C upon irradiation at 395 nm (**a**) and 505 nm (**b**). (**c**) Thermal recovery at 25 °C after irradiation at 395 nm. (**d**) Fatigue study of **BODIPY/Motor** in toluene. Repetitive photoisomerization with 395 nm excitation (*hν*) and thermal recovery (Δ) at 25 °C.

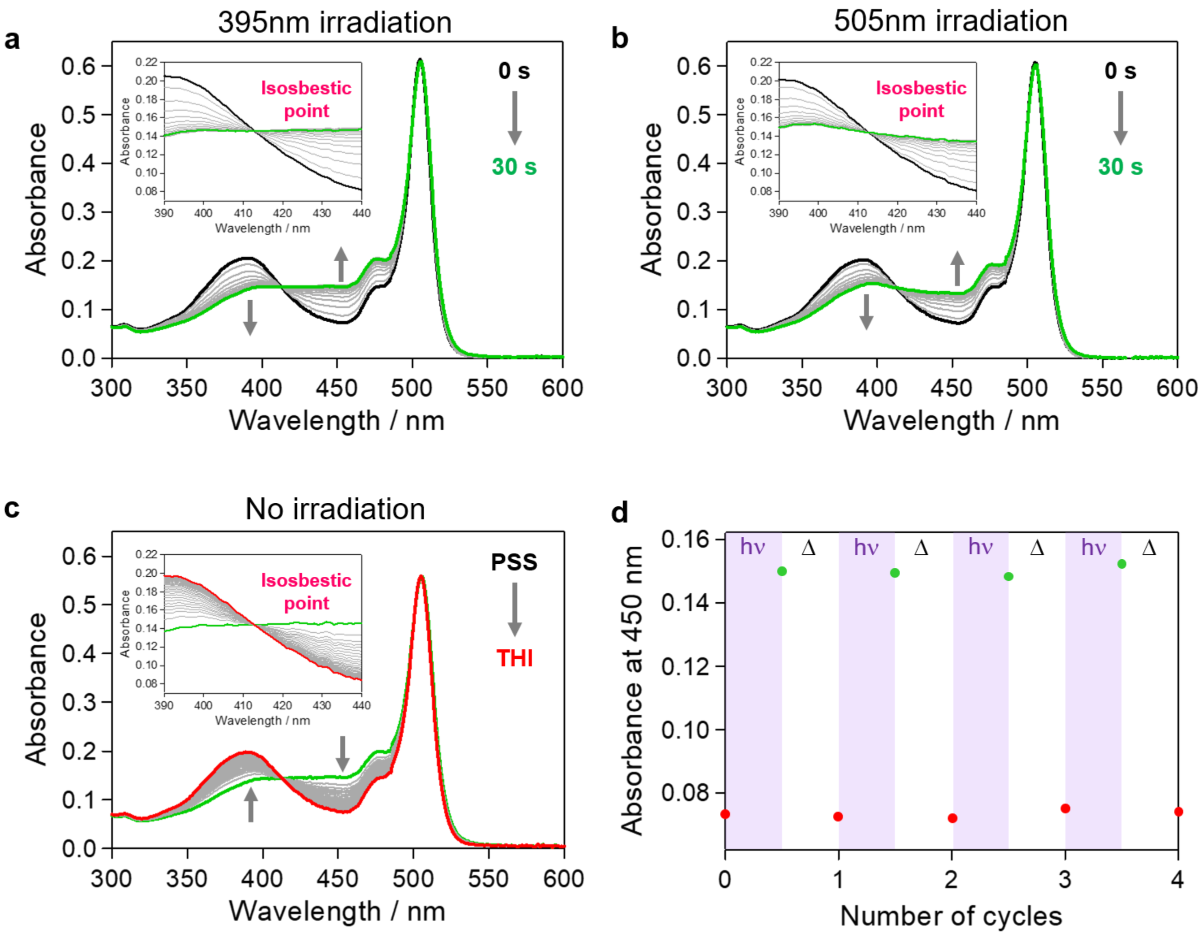

Supplementary Fig. 11. (**a**–**b**) UV/Vis spectral change of **BODIPY/Motor** in THF at 5 °C upon irradiation at 395 nm (**a**) and 505 nm (**b**). (**c**) Thermal recovery at 25 °C after irradiation at 395 nm. (**d**) Fatigue study of **BODIPY/Motor** in THF. Repetitive photoisomerization with 395 nm excitation (*hν*) and thermal recovery (Δ) at 25 °C.

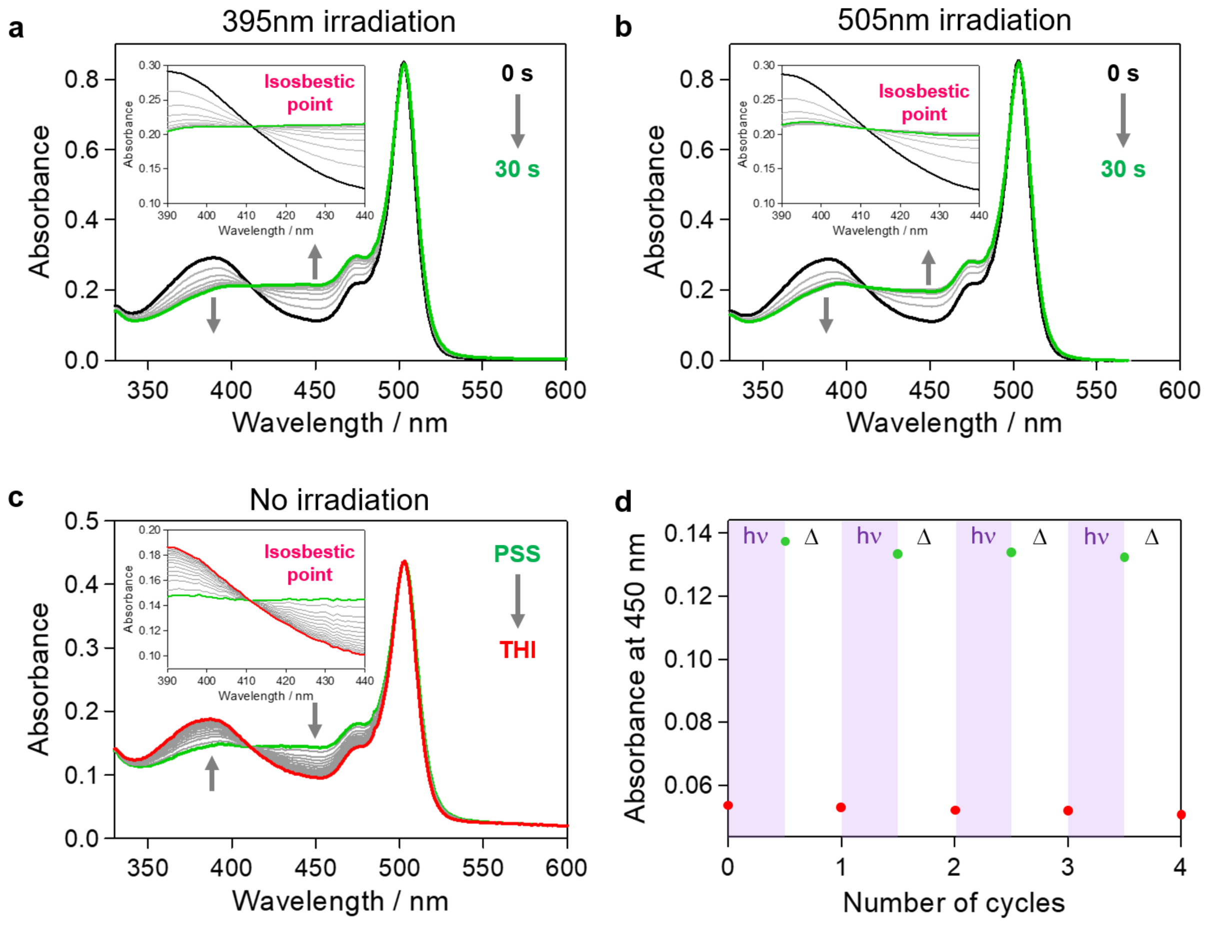

Supplementary Fig. 12. (**a**–**b**) UV/Vis spectral change of **BODIPY/Motor** in acetone at 5 °C upon irradiation at 395 nm (**a**) and 505 nm (**b**). (**c**) Thermal recovery at 25 °C after irradiation at 395 nm. (**d**) Fatigue study of **BODIPY/Motor** in acetone. Repetitive photoisomerization with 395 nm excitation (*hν*) and thermal recovery (Δ) at 25 °C.

The thermal isomerization process was studied at different temperatures (from 5 °C to 30 °C) in the dark after the irradiation. The excitation wavelength was selected at 395 nm, which is close to the largest UV/Vis spectral change (Supplementary Fig. 10a, 11a and 12a). The rate constant, k, was determined by fitting a 1st order rate law and the ln(k/T) values were plotted against 1/T (Supplementary Fig. 13). Thermodynamic parameters for the metastable isomer formation were calculated using the Eyring equation and summarized in Supplementary Table 5.

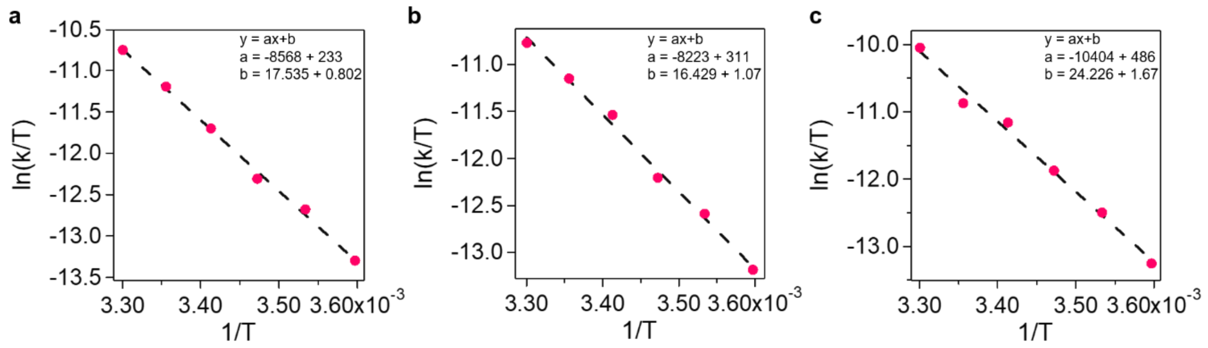

Supplementary Fig. 13. Eyring plots for THI of **BODIPY/Motor** in toluene (**a**), THF (**b**), acetone (**c**).

Supplementary Table 5. Summary of thermodynamic parameters of **BODIPY/Motor** in various solvents obtained from Eyring plot analysis.

Samples	Solvent	$\Delta G^{\ddagger}$ (20 °C) kJ/mol	$\Delta H^{\ddagger}$ (20 °C) kJ/mol	$\Delta S^{\ddagger}$ (20 °C) J/mol K	$t_{1/2}$(20 °C) min
**BODIPY/Motor**	Toluene	86.7	71.2	-51.8	4.8
	THF	86.5	68.4	-60.9	4.0
	Acetone	85.3	86.5	3.88	2.8
Bare **Motor**	Hexane	85.0[a]	-	-	3.2[a]

[a] Value previously reported in Reference 14.

7. Photoisomerization quantum yields of BODIPY/Motor

For a typical experiment, a stirred solution (2 mL) of a compound was irradiated from the side in a fluorescence quartz cuvette (width = 1.0 cm) using a custom-built (Prizmatix/Mountain Photonics) multi-wavelength fiber coupled LED-system (FC6-LED-WL) using the following LEDs: 365A, 390B, 535R. The FWHM was ≤ 20 nm with the exception of the 535R LED (FWHM = 90 nm). A lower FWHM ~ 24 nm for the 535R LED was achieved by connecting the LED through a polymer optic fiber to two collimators (2 x PRI FCM1-0.6) with a filter holder (PRI Filter holder) containing a 1” band pass filter (Chroma Narrow Green MV532/20). All LEDs were connected through a 7 to 1 fiber bundle attached to a 3 mm liquid light guide (LLG-3) and a liquid light guide adapter (LLG-AC). The adapter was placed in a Thorlabs SMR1 lens mount, which was adjusted to height using Thorlabs TR20/30 optical posts, AS6M4M adapters and a PJ302/M Offset Mounting Post Joist. The LEDs were controlled automatically via the built-in USB-controller using FC-LED-Ctrl 3.0. For all kinetic experiments the temperature was maintained using a Quantum Northwest TC1 temperature controller.

A modification of a standard protocol was applied for the determination of the photon flux[15,16]. Different fresh samples of an aqueous H_2SO_4 solution (0.05 M) containing freshly recrystallized $K_3[Fe(C_2O_4)_3]$ (41 mM, 2 mL, 1 cm quartz cuvette) were irradiated at 20 °C for increasing periods of time with a 365 nm LED under constant stirring and with exclusion of other light sources. The solutions were then diluted with 1.0 mL of an aqueous H_2SO_4 solution (0.5 M) containing phenanthroline (1 g/L) and NaOAc (122.5 g/L) and left to react for 10 min under stirring. The absorption at λ= 510 nm was measured and compared to an identically prepared non-irradiated sample, which was used as a blank reference. The concentration of $[Fe(phenanthroline)_3]^{2+}$ complex was calculated using its molar absorptivity (ε = 11100 M^{-1} cm^{-1}) and considering the dilution. The quantity of Fe^{2+} ions expressed in mol was plotted versus time (expressed in seconds, s) and the slope, obtained by linear fitting the data points to the equation $y = ax + b$, equals the rate of formation of the Fe^{2+} ion at the given wavelength. This rate can be converted into the photon flux (I) by dividing it by the quantum yield of formation of the $[Fe(phenanthroline)_3]^{2+}$ complex at the wavelength of interest (Φ^{535nm} = 0.51, Φ^{390nm} = 1.28, Φ^{365nm} = 1.21)[17] and by the probability of photon absorption of the Fe^{3+} complex (1 for 365 nm and 390 nm, and 0.02 for 535nm). Multiplying the results for the Avogadro number gives the following photon fluxes: $I^{365nm} = 2.14 \cdot 10^{16}$ s^{-1}, $I^{390nm} = 2.24 \cdot 10^{16}$ s^{-1}, $I^{535nm} = 3.72 \cdot 10^{15}$ s^{-1}.

Solutions of the **BODIPY/Motor** and **Motor** stable isomers (S) in acetone or toluene (40–50 μM, to obtain an absorbance of ca. 1 at the wavelength of irradiation) were irradiated with an LED of a selected wavelength (i.e. 390 and 535 nm for **BODIPY/Motor**, 365 nm for **Motor**)

to lead to the formation of the respective metastable isomers (M). The spectra were collected following the evolution of the absorption at the wavelength of irradiation, after which the data subsequently fitted using COPASI 4.29[18] following the same approach developed by Stranius & Börjesson[15] Eq. 14 in the original article as follows:

$$\frac{d[\text{Stable}]}{dt} = -\frac{QY_{SM} \cdot I \cdot \beta_S(t)}{N_A \cdot V} + \frac{QY_{MS} \cdot I \cdot \beta_M(t)}{N_A \cdot V} + k_{MS'}[\text{Metastable}] \qquad (2)$$

Equation (2) was used to determine both approximated QYs (QY_{SM} for the forward reaction, i.e. stable-to-metastable transformation, and QY_{MS} for the photochemical backreaction, i.e. metastable-to-stable transformation). The kinetic constant $k_{MS'}$ related to the thermal formation of the next stable state via THI was considered in the equation for the analysis. *I* is the photon flux, previously determined with ferrioxalate actinometry, N_A the Avogadro number, *V* the total volume of the irradiated solution (2 mL) and β the fractions of photons absorbed by either the stable or the metastable state. The decay was fitted by the ODE solver present in COPASI, using a Levenberg-Marquardt algorithm with randomized initial conditions. To obtain physically sound results, the boundaries for the QYs values were fixed between $1 \cdot 10^{-6}$ and 1. The measurements were triplicated and averaged to afford the values presented in Supplementary Table 6.

Supplementary Table 6. Summary of photoisomerization quantum yields (QYs) for forward reaction (stable-to-metastable) and back reaction (metastable-to-stable) of **BODIPY/Motor** and **Motor** in toluene and acetone with 390 nm, 535 nm (for **BODIPY/Motor)** and 365 nm (for **Motor)** excitation wavelengths measured at 5 °C. All the quantum yields have errors of ±5%.

Excitation wavelength (nm)	Photoisomerization reaction	Solvent	
		Toluene	Acetone
365	**Motor**$_S$ to **Motor**$_M$	18%	12%
	**Motor**$_M$ to **Motor**$_S$	42%	21%
390	**BODIPY/Motor**$_S$ to **BODIPY/Motor**$_M$	12%	22%
	**BODIPY/Motor**$_M$ to **BODIPY/Motor**$_S$	51%	18%
535	**BODIPY/Motor**$_S$ to **BODIPY/Motor**$_M$	3%	4%
	**BODIPY/Motor**$_M$ to **BODIPY/Motor**$_S$	4%	4%

8. Potential energy surface

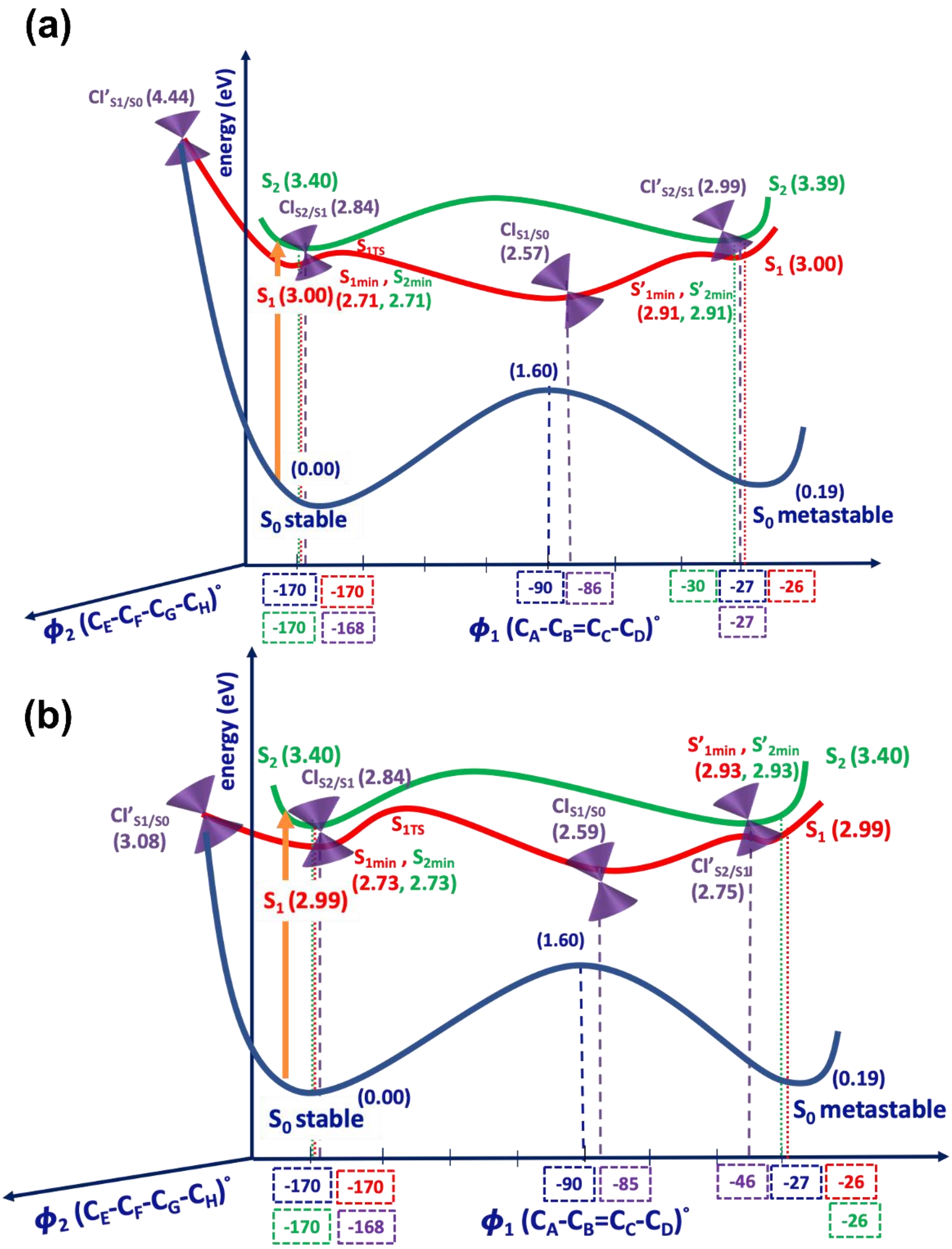

Supplementary Fig. 14. Schematic representation of the PESs of **BODIPY/Motor** along ϕ_1 and ϕ_2 dihedral angles in the gas phase (**a**) and toluene (**b**). The ground state (S_0), first (S_1) and second (S_2) excited states are depicted in dark blue, red and green, respectively. The energies relative to **BODIPY/Motor** stable ground state energy. The purple cones represent the CIs.

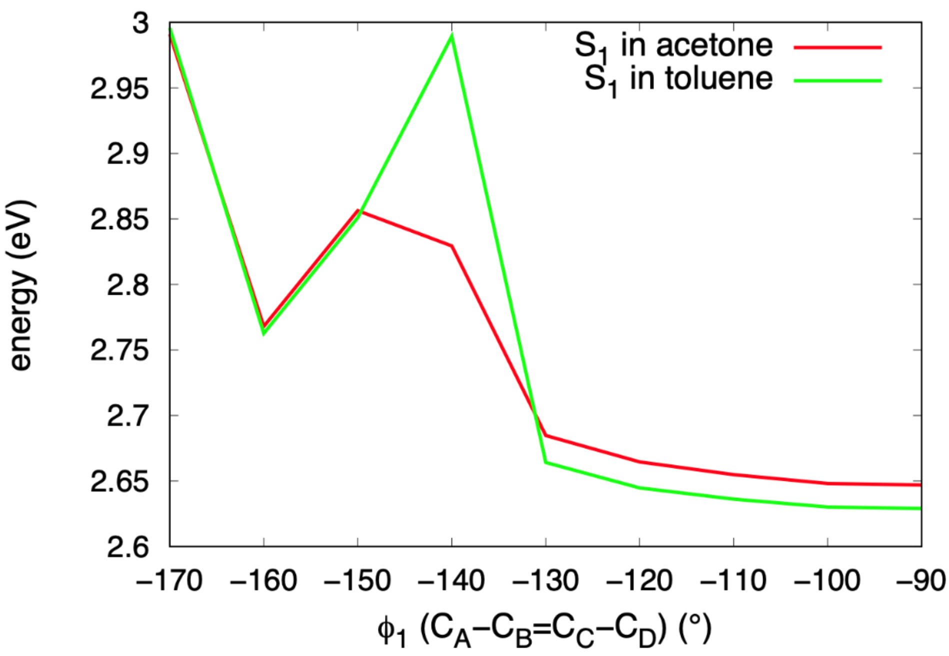

Supplementary Fig. 15. Relaxed S_1-PES scan of the **BODIPY/Motor** stable isomer along ϕ_1 dihedral angle in acetone and toluene.

Supplementary Table 7. The ground state (S_0) optimized geometry for the **BODIPY/Motor** stable isomer in acetone and toluene.

acetone				toluene			
C	2.7080564242	-0.5742315133	-0.9028528243	C	2.7125230466	-0.5671872371	-0.9010283195
C	1.2456839411	-0.7431316652	-0.6864848423	C	1.2488940978	-0.7304623377	-0.6880870948
C	0.5084534598	-1.4448643644	-1.6136911567	C	0.5114454821	-1.4276907842	-1.6188473334
C	-0.8850644364	-1.5470088615	-1.4513966080	C	-0.8821565195	-1.5292419323	-1.4598313188
C	-1.5333701038	-0.9056928764	-0.4101426716	C	-1.5327048218	-0.8929463803	-0.4168755906
C	-0.7831706176	-0.3004638518	0.6433187557	C	-0.7832860713	-0.2902883454	0.6384992756
C	0.6302268756	-0.1957979206	0.4824226904	C	0.6305078373	-0.1854644349	0.4798491313
C	-1.3664595397	0.1451845390	1.8599347195	C	-1.3684979127	0.1536410563	1.8546451328
C	-0.5957010088	0.7149068280	2.8440021876	C	-0.5989140472	0.7217591040	2.8398351601
C	0.7995094303	0.8712075271	2.6605800985	C	0.7963365124	0.8778125148	2.6590070517
C	1.3986996777	0.4194531237	1.5101539938	C	1.3976793282	0.4280857050	1.5094586304
C	-2.9848091002	-1.1326974510	-0.5554190728	C	-2.9839556956	-1.1238449819	-0.5611051845
C	-3.1018537487	-2.4274770417	-1.3514294490	C	-3.0965305982	-2.4158053376	-1.3630428243
C	-1.8646210657	-2.3294874367	-2.2833922544	C	-1.8613752782	-2.3083645451	-2.2963595713
C	-4.0108440758	-0.2927023577	-0.2546329719	C	-4.0140831184	-0.2914210812	-0.2562636949
C	-3.0256513850	-3.6748829839	-0.4632472305	C	-3.0138810856	-3.6668357803	-0.4807866177
C	-3.9481956018	1.1610522033	0.0548137319	C	-3.9593823772	1.1608988166	0.0611750458
C	-5.2565794689	1.6352997522	0.2917466062	C	-5.2695939916	1.6258573412	0.3025768314
C	-6.1923443582	0.5165499429	0.1377565789	C	-6.1990874238	0.5029037525	0.1434851173
C	-5.4588231967	-0.6340271293	-0.2299114288	C	-5.4602721998	-0.6412519570	-0.2312748800
C	-2.8943619555	2.0786357868	0.0003233648	C	-2.9109623852	2.0837185460	0.0105892999
C	-3.1463414035	3.4275948534	0.2562043486	C	-3.1694482057	3.4292415953	0.2752387253
C	-4.4383508382	3.8769176183	0.5478614406	C	-4.4628181197	3.8693752260	0.5708975135
C	-5.5077764803	2.9809770783	0.5519075707	C	-5.5269663833	2.9679377863	0.5707606688
C	-7.5756026462	0.4798525660	0.2964236756	C	-7.5809838432	0.4568070222	0.3044250517
C	-8.2450241792	-0.7265192726	0.0870946406	C	-8.2442013755	-0.7516146144	0.0907559498
C	-7.5290309484	-1.8750540727	-0.2629543766	C	-7.5230565121	-1.8938356429	-0.2657280091
C	-6.1407494291	-1.8410803262	-0.4144464561	C	-6.1357347384	-1.8506031765	-0.4194651369
C	3.1711160603	0.5917409203	-1.5225679720	C	3.1808852993	0.5932086530	-1.5259184161
C	3.5921964761	-1.5764012239	-0.4937745208	C	3.5906914608	-1.5726498414	-0.4870481111
C	2.4803827196	1.7452232700	-2.0025466412	C	2.4959384859	1.7496993794	-2.0087022643
C	3.4608060760	2.5875231100	-2.5188993972	C	3.4796650072	2.5817910273	-2.5316625976
C	4.7116779428	1.9629202131	-2.3586568131	C	4.7274252339	1.9479222240	-2.3724219644

N 4.5352965061 0.7713783472 -1.7606383172	N 4.5442853058 0.7626368853 -1.7690540515
N 4.9643707179 -1.4429769560 -0.7180440996	N 4.9618551806 -1.4493108433 -0.7146705767
C 5.5865062019 -2.5228626598 -0.2141958223	C 5.5785414102 -2.5284586103 -0.2082278443
C 4.6285905881 -3.3832730389 0.3552488748	C 4.6167112310 -3.3807457967 0.3693085587
C 3.3719080747 -2.8089221505 0.1924154269	C 3.3639846438 -2.8001196492 0.2067654684
C 7.0628153347 -2.7135437015 -0.2819652688	C 7.0537564659 -2.7235733134 -0.2826822664
C 6.0505782408 2.4733652200 -2.7679881834	C 6.0703095860 2.4425782408 -2.7872289774
H 1.0025853250 -1.9105411230 -2.4686069539	H 1.0086311154 -1.8900333982 -2.4739526239
H -2.4391340563 0.0210573023 2.0074439591	H -2.4416328809 0.0314242794 1.9989847752
H -1.0593084064 1.0456634225 3.7749859260	H -1.0645339404 1.0524698513 3.7696671092
H 1.4013735110 1.3353481077 3.4435882490	H 1.3971153732 1.3409767581 3.4433153402
H 2.4783771564 0.5158616129 1.3837909352	H 2.4772748833 0.5242970254 1.3834303723
H -4.0255457130 -2.4438991482 -1.9428379568	H -4.0209924963 -2.4320344640 -1.9534352529
H -2.1202164332 -1.7812538492 -3.2047562310	H -2.1205520311 -1.7544366908 -3.2134873758
H -1.4722133700 -3.3137418657 -2.5773965810	H -1.4679102312 -3.2900010807 -2.5988873498
H -3.0940969191 -4.5849167639 -1.0779452802	H -3.0810941512 -4.5763974015 -1.0967759416
H -3.8398294895 -3.6939971887 0.2761455000	H -3.8246234685 -3.6907557448 0.2620805941
H -2.0709058081 -3.7018119263 0.0860363978	H -2.0579562773 -3.6922082849 0.0663886533
H -1.8822795378 1.7659743176 -0.2487142514	H -1.8980050327 1.7769337406 -0.2413860327
H -2.3198172825 4.1400071908 0.2215370367	H -2.3468571387 4.1460917276 0.2444735530
H -4.6135963643 4.9352389873 0.7501482578	H -4.6435308122 4.9253842147 0.7797281095
H -6.5253036492 3.3310634242 0.7371220303	H -6.5457990121 3.3120036905 0.7591046977
H -8.1263964928 1.3782871793 0.5825057629	H -8.1365466571 1.3502566163 0.5962395675
H -9.3291384393 -0.7757204739 0.2044284091	H -9.3276429603 -0.8073048262 0.2100602822
H -8.0585436755 -2.8173358148 -0.4158898331	H -8.0475847639 -2.8382788718 -0.4215986494
H -5.6191643043 -2.7626067973 -0.6649617873	H -5.6089950833 -2.7678852701 -0.6740160013
C 1.0149935547 2.0494762756 -1.9705410453	C 1.0328879090 2.0647000762 -1.9678519112
H 3.3022322141 3.5634521902 -2.9728067069	H 3.3266772558 3.5570125333 -2.9886985677
H 4.8489927617 -4.3308366894 0.8421177228	H 4.8327022764 -4.3265688595 0.8612765217
C 2.0845289794 -3.4017155026 0.6736676666	C 2.0737011131 -3.3795608794 0.6972837466
H 7.4022227096 -2.7165558719 -1.3280063136	H 7.3862119101 -2.7248172181 -1.3308513736
H 7.5807728618 -1.8862680834 0.2243455621	H 7.5744407412 -1.8949467470 0.2183640026
H 7.3451444093 -3.6616573042 0.1911148478	H 7.3377969178 -3.6717829752 0.1895091474
H 5.9557818471 3.4674591954 -3.2212888756	H 5.9861812508 3.4342703552 -3.2480896703
H 6.7222782804 2.5321445356 -1.8995898454	H 6.7429460329 2.4982625625 -1.9195045603
H 6.5162965762 1.7887747405 -3.4919777048	H 6.5278988072 1.7454522599 -3.5042514193
B 5.6410906383 -0.2440667249 -1.4120466894	B 5.6503383699 -0.2577590974 -1.4182678652
F 6.5895184259 0.3469423877 -0.5448212704	F 6.5951779371 0.3316989703 -0.5532555830
F 6.3048617425 -0.6684058265 -2.5867891999	F 6.3009835593 -0.6936315998 -2.5901261221
H 0.8172573322 2.9878306221 -2.5055337174	H 0.8369714737 3.0020943182 -2.5053997799
H 0.6520178314 2.1606544459 -0.9371114050	H 0.6789443106 2.1834669927 -0.9320939214
H 0.4199892944 1.2495258222 -2.4338591629	H 0.4273872490 1.2680219627 -2.4231587277
H 2.2886256663 -4.3108289221 1.2550006866	H 2.2721911563 -4.2879187737 1.2819636379
H 1.5264765910 -2.6992878780 1.3099085690	H 1.5251486535 -2.6701694071 1.3340802098
H 1.4252230726 -3.6689480503 -0.1653970341	H 1.4064057304 -3.6454345982 -0.1358815584

Supplementary Table 8. The ground state (S_0) optimized geometry for the **BODIPY/Motor** metastable isomer in acetone and toluene.

acetone	toluene
C 3.0163534878 -0.6326172289 0.9158079028	C 3.0192325402 -0.6366458337 0.9290611047
C 1.5893932455 -0.5151592859 0.5107034478	C 1.5893231062 -0.5237588471 0.5328561923
C 1.0521208075 -1.4580955267 -0.3338861134	C 1.0498654665 -1.4712875150 -0.3057007061
C -0.3215746232 -1.4107620922 -0.6363580901	C -0.3229892222 -1.4241957694 -0.6095861822
C -1.1564294417 -0.4657807195 -0.0661133513	C -1.1577651731 -0.4738264339 -0.0481243516
C -0.5924126375 0.6296246370 0.6698995573	C -0.5927253231 0.6224592668 0.6853853601
C 0.7971331640 0.5759605958 0.9896713453	C 0.7963592027 0.5675613797 1.0079695799
C -1.3231233130 1.7906786039 1.0377543764	C -1.3222978410 1.7854095259 1.0490871075
C -0.7273018297 2.8113402650 1.7401119406	C -0.7262771798 2.8051957008 1.7514024454
C 0.6322736634 2.7229555309 2.1188220488	C 0.6319807082 2.7153326439 2.1336117495
C 1.3790746105 1.6328312011 1.7429278378	C 1.3783850967 1.6244590823 1.7611773057

C -2.5411343646 -0.7053973212 -0.5017797859	C -2.5415152362 -0.7085486689 -0.4911377365
C -2.4687542828 -1.6353261965 -1.7184416042	C -2.4657311947 -1.6458462660 -1.7027176744
C -1.0778746456 -2.3103599787 -1.5625401495	C -1.0801401667 -2.3281502971 -1.5320548276
C -3.6817682713 -0.2959700803 0.1294118765	C -3.6850939832 -0.2919780487 0.1279128858
C -3.5566519234 -2.7049750491 -1.8179784300	C -3.5589054454 -2.7101341706 -1.8035811349
C -5.0120162282 -0.0744623431 -0.4821185574	C -5.0092575467 -0.0693270780 -0.4968003367
C -5.9277378094 0.3015548833 0.5254814774	C -5.9325950624 0.3131457679 0.5003714266
C -5.2270954705 0.2830580105 1.8134651555	C -5.2435684798 0.2993796673 1.7946150104
C -3.8711521739 -0.0373639092 1.5791468289	C -3.8868100547 -0.0246680427 1.5746255187
C -5.4050475082 -0.0080198449 -1.8227528055	C -5.3887266597 -0.0053746405 -1.8406126888
C -6.7182533735 0.3482045318 -2.1328154292	C -6.6972564699 0.3531925994 -2.1653074961
C -7.6343437310 0.6669782672 -1.1232537509	C -7.6214875643 0.6771259984 -1.1661078891
C -7.2383006062 0.6605171448 0.2146558711	C -7.2381213462 0.6742982910 0.1750188531
C -5.7121593767 0.4841354077 3.1048366615	C -5.7386808277 0.5072872035 3.0801374650
C -4.8360445902 0.3463273819 4.1814581890	C -4.8724137196 0.3736552155 4.1646331697
C -3.5014422485 -0.0144473240 3.9619664332	C -3.5372285355 0.0101359827 3.9589968192
C -3.0148198446 -0.2181324504 2.6704340539	C -3.0403225695 -0.2012894525 2.6731512554
C 4.0219159033 -0.1196554999 0.0917995112	C 4.0154872307 -0.1129530440 0.1004501152
C 3.3236181553 -1.2695069974 2.1233906071	C 3.3382635473 -1.2821016518 2.1283858608
C 3.9598215299 0.6019262883 -1.1385439293	C 3.9399035138 0.6184547077 -1.1237752806
C 5.2787845350 0.8772204454 -1.4850090164	C 5.2541863274 0.8972501469 -1.4810116386
C 6.1195800985 0.3399064558 -0.4917828409	C 6.1053659846 0.3522505278 -0.4993959512
N 5.3654366772 -0.2525086385 0.4501939864	N 5.3615061250 -0.2463890477 0.4438118912
N 4.6525382076 -1.4155594318 2.5268354917	N 4.6701738829 -1.4323681820 2.5152670799
C 4.6724527193 -2.0527306746 3.7107638836	C 4.7045107499 -2.0784501671 3.6914295283
C 3.3509601218 -2.3325295889 4.1054924417	C 3.3866480466 -2.3608336009 4.1006872652
C 2.4884991018 -1.8507143242 3.1252968125	C 2.5138257100 -1.8702887831 3.1357386287
C 5.9328485277 -2.3803911949 4.4349738102	C 5.9772244344 -2.4101836039 4.3913620548
C 7.6074380750 0.3870615290 -0.4268870516	C 7.5935246955 0.3967247036 -0.4440298182
H 1.6812174760 -2.2487563815 -0.7471423713	H 1.6804614237 -2.2634739010 -0.7140840258
H -2.3678213903 1.8747732302 0.7435246816	H -2.3662518574 1.8701627890 0.7532798550
H -1.3062956957 3.6987061562 2.0022831768	H -1.3051837739 3.6929842974 2.0116790622
H 1.0933559866 3.5313486810 2.6885931478	H 1.0926518350 3.5232768147 2.7041958910
H 2.4372051046 1.5814063665 2.0047543729	H 2.4357014832 1.5699482149 2.0251595176
H -2.4551027519 -1.0263421894 -2.6403513549	H -2.4403529159 -1.0429372050 -2.6286210072
H -0.5577328785 -2.4471438545 -2.5212567196	H -0.5562269705 -2.4785119343 -2.4871017377
H -1.1974680635 -3.3110914244 -1.1142007975	H -1.2098537845 -3.3242068114 -1.0758591392
H -3.3056408404 -3.4009660994 -2.6329715965	H -3.3074803148 -3.4143348006 -2.6116034807
H -4.5541900197 -2.3008725004 -2.0199893850	H -4.5533225302 -2.3025495660 -2.0130798602
H -3.6060415771 -3.2823256551 -0.8811712627	H -3.6192970063 -3.2792563499 -0.8626183725
H -4.6996100177 -0.2149752111 -2.6283089020	H -4.6752298703 -0.2155806105 -2.6380966380
H 5.6169403062 1.4180540664 -2.3662420232	H 5.5830812770 1.4453530521 -2.3611394634
H 6.5880757018 -2.9994420044 3.8051264246	H 6.6185131856 -3.0261984989 3.7443803437
H 6.4879485275 -1.4624694920 4.6763914560	H 6.5383317581 -1.4924216330 4.6189570276
H 5.7079210442 -2.9204244308 5.3625378839	H 5.7713904538 -2.9516354846 5.3226347868
H 8.0120596985 0.8691249489 -1.3248704650	H 7.9948331208 0.8888946134 -1.3381505552
H 7.9346615652 0.9455295808 0.4621249409	H 7.9255529015 0.9415061665 0.4515542549
H 8.0206279461 -0.6282545997 -0.3417824228	H 8.0034250690 -0.6207781322 -0.3712055913
B 5.8830472518 -0.9482696319 1.7249079003	B 5.8978718176 -0.9513290763 1.7102359288
F 6.6529791703 -0.0457061393 2.4941435282	F 6.6581279129 -0.0481036324 2.4805322345
F 6.6933074409 -2.0564835061 1.3818028702	F 6.7051008352 -2.0500682070 1.3509373370
C 0.9951065607 -1.9407209215 3.1757076168	C 1.0204968755 -1.9523177934 3.2035853707
H 0.5806785218 -2.4097118009 2.2718347911	H 0.5921598078 -2.4222360365 2.3066508954
H 0.6874355823 -2.5317634197 4.0486824949	H 0.7186224484 -2.5391888469 4.0815175686
H 0.5367893486 -0.9431158801 3.2580295244	H 0.5688355084 -0.9518270973 3.2884779358
C 2.7572872869 1.0109603048 -1.9296720012	C 2.7274775813 1.0355220863 -1.8958251526
H 3.0628523399 1.6569812353 -2.7634694933	H 3.0232326781 1.6770511584 -2.7368448785
H 2.2329895674 0.1373938143 -2.3444356061	H 2.1863550512 0.1669452206 -2.2990291812
H 2.0309216186 1.5588555774 -1.3119682276	H 2.0170788170 1.5925064074 -1.2675353472
H -1.9767677583 -0.5177533923 2.5301075077	H -2.0017469153 -0.5028813134 2.5417083937
H -2.8290021220 -0.1436240560 4.8123084279	H -2.8724173465 -0.1147925335 4.8157039139
H -5.1964068989 0.5018698605 5.2000579177	H -5.2413590409 0.5349632920 5.1791178284
H -6.7628276999 0.7306071369 3.2717138908	H -6.7901830039 0.7557396487 3.2373333001

H	-7.9377149546	0.9487649198	1.0021316697	H	-7.9444871347	0.9680569415	0.9539564389
H	-7.0302854279	0.3877382201	-3.1782174259	H	-6.9988747079	0.3909904556	-3.2136792818
H	-8.6568452469	0.9441980022	-1.3865591518	H	-8.6405063196	0.9563835897	-1.4399212490
H	3.0667809604	-2.8416149895	5.0239343400	H	3.1128548843	-2.8760745505	5.0187251167

Supplementary Table 9. Optimized geometry parameters of the S_0, S_{1min}, S_{2min}, and $CI'_{S1/S0}$ and $CI_{S2/S1}$. The energies are relative to S_{0min} energy of **BODIPY/Motor** stable isomer (gas phase/acetone/toluene). Note that the $CI'_{S1/S0}$ is located at the BODIPY folding reaction coordinate starting from stable structure, which is not the case for the metastable. All attempts to optimize $CI_{S2/S1}$ of metastable isomer crossing in toluene did not converge using the penalty function method. We found a $CI_{S1/S0}$ structure which shows close similarities with the corresponding geometry of the metastable isomer in acetone.

	Stable			Metastable		
Geometry	Energy (eV)	ϕ_1 (°)	ϕ_2 (°)	Energy (eV)	ϕ_1 (°)	ϕ_2 (°)
S_0	0.0/0.0/0.0	-170/-170/-170	-87/-86/-86	0.10/0.19/0.19	-27/-27/-27	-95/-95/-94
S_{1min}	2.71/2.74/2.73	-170/-170/-170	-66/-65/-66	2.91/2.93/2.93	-26/-26/-26	-115/-115/-115
S_{2min}	2.71/2.74/2.73	-170/-170/-170	-66/-65/-65	3.31/2.93/2.93	-30/-26/-26	-108/-115/-115
$CI'_{S1/S0}$	4.44/3.04/3.08	-170/-168/-167	-61/-3/3	-	-	-
$CI_{S2/S1}$	2.84/2.83/2.84	-168/-168/-168	-60/-62/-61	2.99/2.69/2.75	-27/-41/-46	-120/-120/-119

Supplementary Table 10: Mulliken charges on the lower half of motor, upper half of Motor and BODIPY at the S_{0min}, S_{1min}, S_{2min}, CI_{S2S1}, $CI_{S1/S0}$, $CI'_{S1/S0}$ and transition state (TS) of the S_1 state.

	Lower half of Motor		Upper half of Motor		BODIPY	
	acetone	toluene	acetone	toluene	acetone	toluene
S_{0min}	-0.05	-0.03	0.05	0.03	0.00	0.00
S_{1min}	-0.09	-0.07	0.03	0.00	0.06	0.07
S_{2min}	-0.09	-0.07	0.03	0.03	0.06	0.07
CI_{S2S1}	-0.11	-0.10	0.03	0.01	0.08	0.09
S_{1TS}	-0.13	-0.14	0.07	0.08	0.06	0.06
$CI_{S1/S0}$	0.01	0.01	0.01	0.00	-0.02	-0.01
$CI'_{S1/S0}$	-0.09	-0.07	0.18	0.18	-0.09	-0.11

9. Time-resolved PL

To obtain further insights into the solvent dependence of the excited state dynamics of **BODIPY/Motor**, time-resolved PL spectroscopy was applied. Samples of **BODIPY/Motor** dissolved various solvents with different polarity indexes (Supplementary Table 12) as well as reference samples of **BODIPY** and **BODIPY+Motor** were prepared. The excitation wavelengths of time-resolved PL measurements were selected at 400 nm and 510 nm, to reach the S_2 and S_1 excited states of **BODIPY/Motor** (Supplementary Section 5). The molar concentration of the studied compounds was set in the range between 1×10^{-5} M and 4×10^{-5} M, depending on the particular compounds and solvents. The optical density (OD) at 510 nm of the samples was in a range of $0.03 - 0.14$, corresponding to an OD at 400 nm of $0.007 - 0.06$, depending on the compounds and solvents.

9.1. PL maps and mean energies

Supplementary Fig. 16 shows PL maps of **BODIPY**, **BODIPY/Motor** and **BODIPY+Motor** in toluene and acetone under 400 nm and 510 nm excitations. All maps show a PL band between ca. 490 nm and ca. 570 nm with the time spreading up to ~5 ns, except for the maps of **BODIPY/Motor** in acetone, in which the PL band only appears within ~1 ns time.

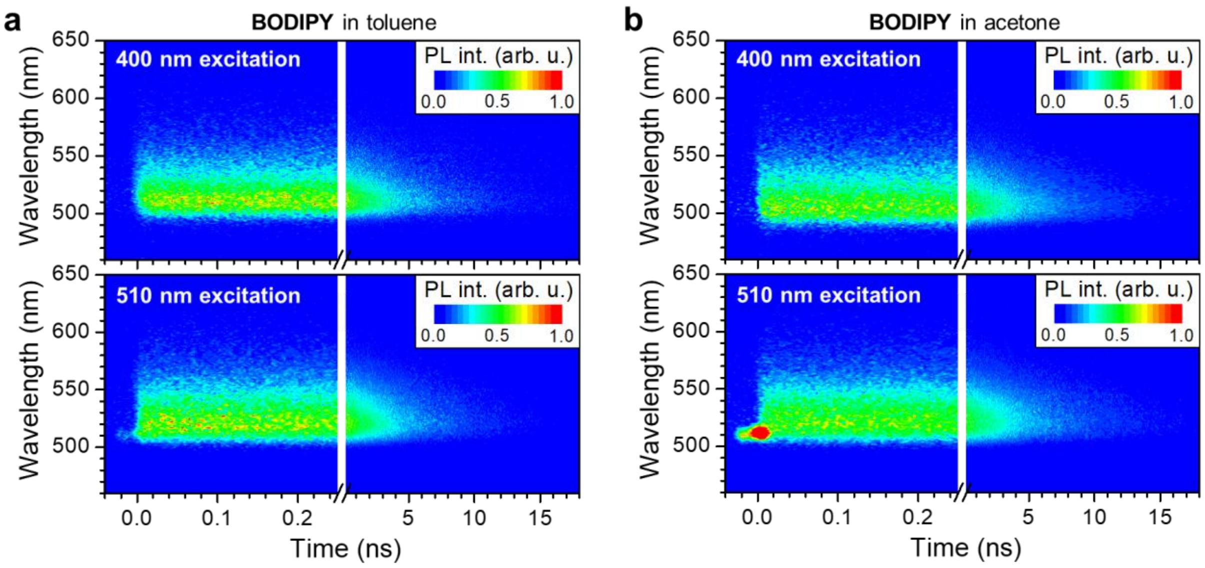

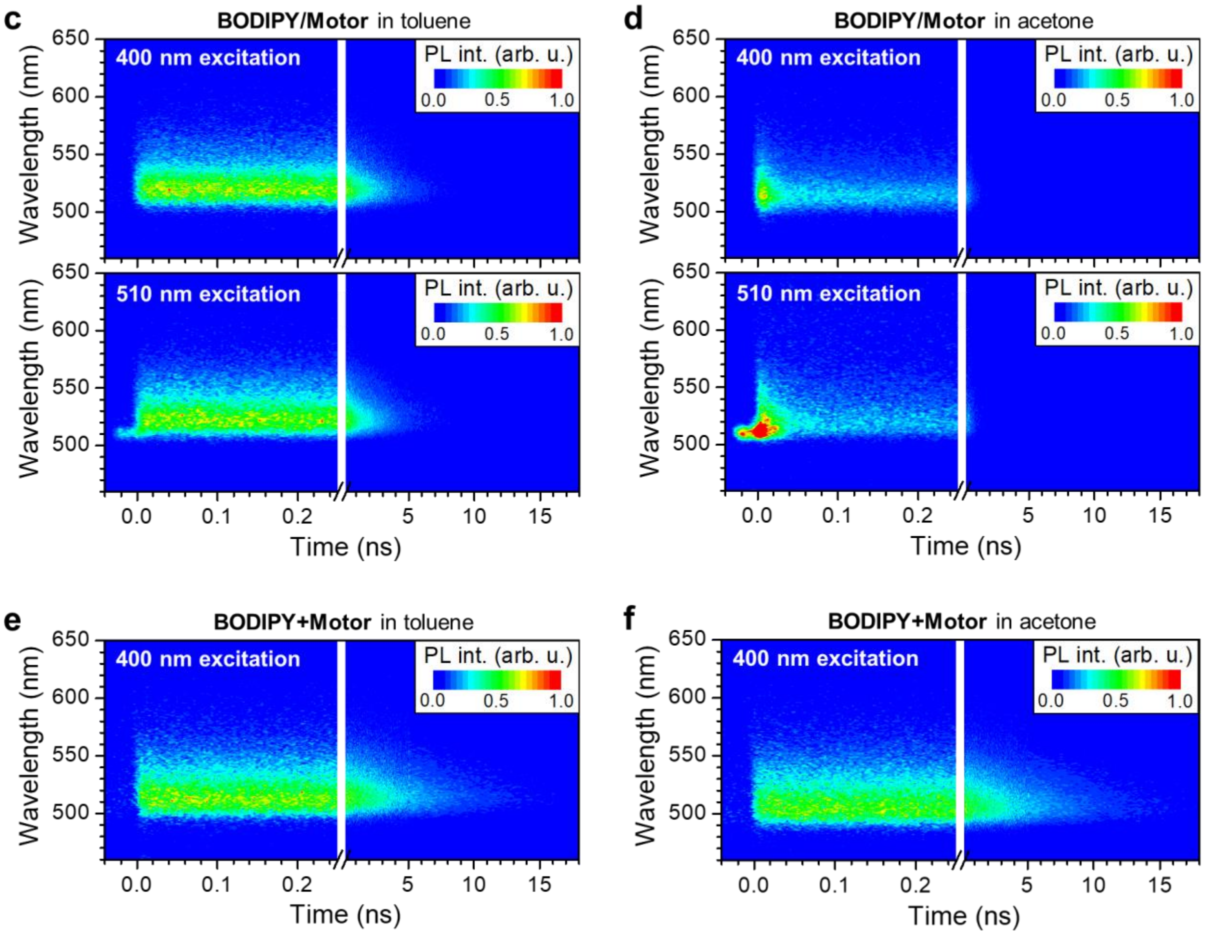

Supplementary Fig. 16. Time-resolved PL maps of **BODIPY** (**a**, **b**), **BODIPY/Motor** (**c**, **d**) and **BODIPY+Motor** (**e**, **f**) in toluene (**a**, **c**, **e**) and acetone (**b**, **d**, **f**) under 400 nm (*upper graphs*) and 510 nm (*lower graphs*) excitations. Strong signals near zero delay time in the PL maps under 510 nm excitation are due to the scattered excitation beam. The molar concentration of all compounds was set in the range between 1×10^{-5} M and 4×10^{-5} M.

Supplementary Fig. 17 shows mean PL energies of **BODIPY**, **BODIPY/Motor** and **BODIPY+Motor** in toluene and acetone under 400 nm and 510 nm excitations. All other samples have no noticeable change in the mean PL energy over time, except of **BODIPY/Motor** in acetone which has a down-hill shift (or redshift) in the mean PL energy of ~50 meV with the decay time of ~15 ps.

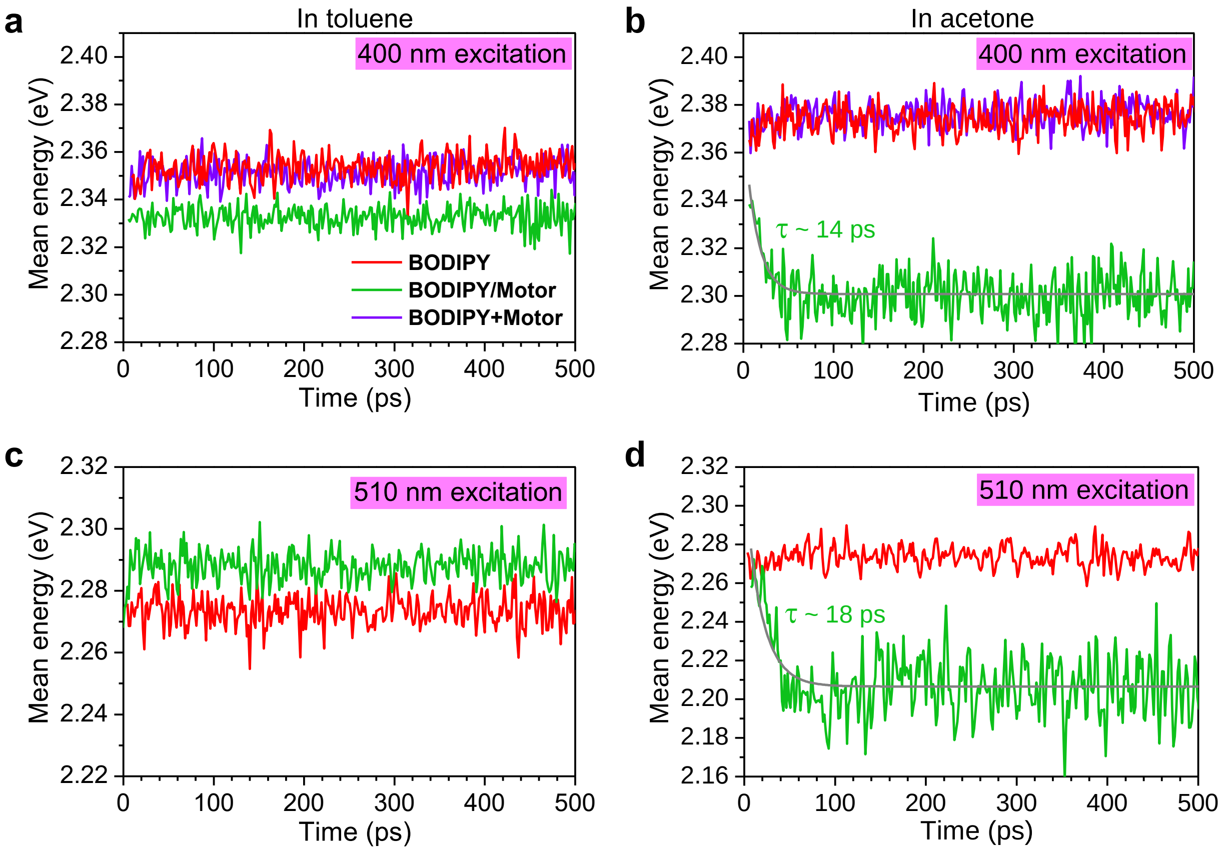

Supplementary Fig. 17. Mean PL energies of **BODIPY** (red), **BODIPY+Motor** (violet) and **BODIPY/Motor** (green) in toluene (**a**, **c**) and acetone (**b**, **d**) under 400 nm (**a**–**c**) and 510 nm (**c**–**d**) excitations. The mean energies were calculated as $\int \omega S(\omega, t) d\omega / \int S(\omega, t) d\omega$ of spectral slices $S(\omega, t)$ at a particular time t from the time-resolved PL maps. To exclude the contribution of the scattered laser beam, all PL maps under 510 nm excitation were cropped to a spectral range of 520–700 nm before the calculation of the mean energy. The grey lines in panels **b** and **d** are the best fits to a single-exponential function with a lifetime τ of the mean PL energies of **BODIPY/Motor** in acetone under 400 nm and 510 nm excitations.

9.2. PL transients

Supplementary Fig. 18 shows PL transients of bare **BODIPY**, **BODIPY/Motor** and **BODIPY+Motor** in toluene and acetone under 400 nm and 510 nm excitations. In all samples, the PL transients under 400 nm excitation are similar to those under 510 nm excitation, indicating no excitation-wavelength dependence of the PL transients. PL of bare **BODIPY** in toluene and acetone under 400 nm and 510 nm excitations decays with a similar lifetime of $4.8 - 5.4$ ns, which is in line with the previous studies[9].

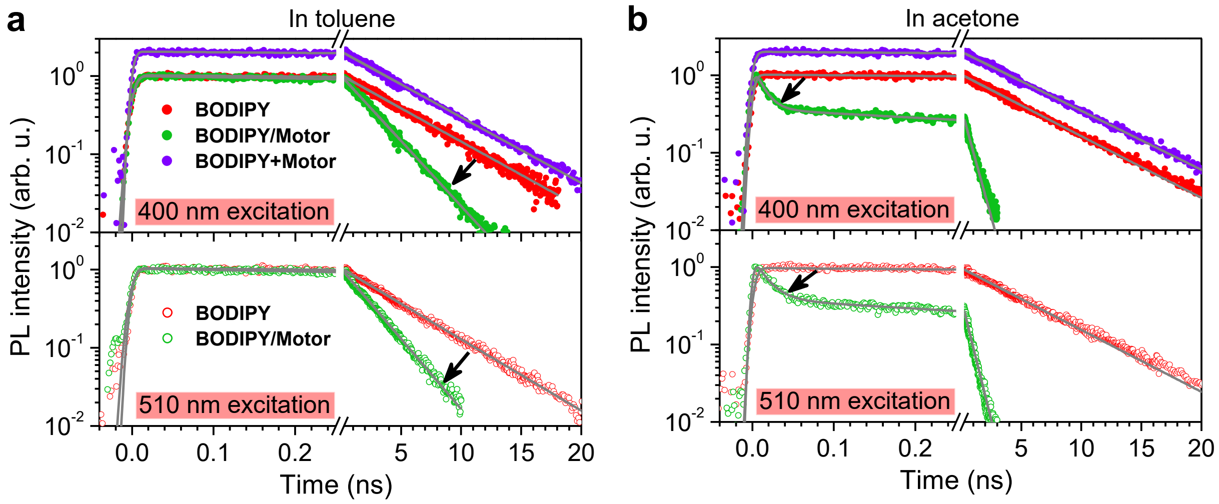

Supplementary Fig. 18. Time-resolved PL transients of **BODIPY** (red), **BODIPY/Motor** (green) and **BODIPY+Motor** (violet) in toluene (**a**) and acetone (**b**) under 400 nm (filled dots) and 510 nm (open dots) excitations. The PL transients were obtained by integrating the PL maps (Supplementary Fig. 16) in the spectral region between 490 nm and 600 nm (between 520 nm and 600 nm for data of PL maps under 510 nm excitation). The grey solid lines show the best fits to exponential functions convoluted to the Gaussian apparatus function of the respective experimental data. The fitting parameters are listed in Supplementary Table 11. The black arrows depict the PL quenching upon attaching the motor core to BODIPY. For the sake of clarity, the transients of **BODIPY+Motor** in toluene and acetone are multiplied by 2.

When the motor core is chemically attached to BODIPY (i.e., **BODIPY/Motor**), PL is shortened to $2.4 - 2.6$ ns with toluene used as the solvent (Supplementary Fig. 18a; green dots). Remarkably, when acetone is used as the solvent, the PL of **BODIPY/Motor** is shortened even more and exhibits distinct bi-exponentiality with the decaying components having the lifetimes of $\tau_1 \sim 12 - 17$ ps and $\tau_2 \sim 0.7 - 0.75$ ns (Supplementary Fig. 18b; green dots). The shortening of the decay time indicates faster depopulation of the excited state of **BODIPY/Motor** in acetone, indicating the PL quenching effect in **BODIPY/Motor**.

PL transients of **BODIPY+Motor**, i.e., a mixture of the two compounds (Supplementary Fig. 18; violet dots) are similar to those of bare **BODIPY**, indicating that there is no PL quenching when mixing the bare **Motor** core and bare **BODIPY** in a solution.

PL transients of bare **BODIPY**, **BODIPY/Motor** and **BODIPY+Motor** in various solvent, such as toluene and acetone, under 400 nm and 510 nm excitations were fitted to exponential functions convoluted with a Gaussian distribution (Equation (3)).

$$y = \frac{1}{\sqrt{2\pi\sigma^2}} e^{-\frac{t^2}{2\sigma^2}} \otimes \sum_i a_i e^{-\frac{t}{\tau_i}} + a_0 \quad (3)$$

where σ is the standard deviation of the Gaussian distribution, and a_i and τ_i are the amplitude and decay time of the ith exponent, respectively. a_0 is the offset of the fitting data (which will be used for TA transients (Supplementary Section 10.2)). The fitting parameters for the PL transients are listed in Supplementary Table 11.

Supplementary Table 11. Fitting parameters for PL transients of compounds in toluene and acetone. Summary of the decay times of the fits (using Equation (3)) for PL transients of bare **BODIPY**, **BODIPY/Motor** and **BODIPY+Motor** in toluene and acetone under 400 nm and 510 nm excitations. The values in the parentheses are the decay amplitudes of the fitting components normalized to their sum.

Parameters	**BODIPY** in toluene		**BODIPY/Motor** in toluene		**BODIPY+Motor** in toluene
	400 nm excitation	510 nm excitation	400 nm excitation	510 nm excitation	400 nm excitation
σ	7 ± 1 ps	6 ± 1 ps	6 ± 1 ps	7 ± 1 ps	5 ± 1 ps
τ	5.1 ± 0.2 ns	4.8 ± 0.2 ns	2.6 ± 0.2 ns	2.4 ± 0.2 ns	5.2 ± 0.1 ns
Parameters	**BODIPY** in acetone		**BODIPY/Motor** in acetone		**BODIPY+Motor** in acetone
	400 nm excitation	510 nm excitation	400 nm excitation	510 nm excitation	400 nm excitation
σ	5 ± 1 ps	5 ± 1 ps	5 ± 1 ps	4 ± 1 ps	5 ± 1 ps
τ_1	–	–	12 ± 1 ps (0.75)	17 ± 2 ps (0.71)	–
τ_2	5.4 ± 0.1 ns	5.3 ± 0.1 ns	0.75 ± 0.1 ns (0.25)	0.7 ± 0.1 ns (0.29)	5.6 ± 0.1 ns

Supplementary Fig. 19 shows PL transients in short-wavelength (490–550 nm) and long-wavelength (580–700 nm) regions of **BODIPY/Motor** in acetone under 400 nm excitation. The short-wavelength PL shows a strong signal in the first ~40 ps followed by a long decay. The long-wavelength PL matches well with the short-wavelength PL at the time above 40 ps. These results are in line with the previously discussed results on the mean PL energy (Supplementary Fig. 17b), which is redshifted in the early time (up to 40 ps) and remains constant for the longer time. The redshift in mean PL energy is assigned to the excited state relaxation, and therefore PL in the long-wavelength (low-energy) region of 580–700 nm is assigned to the excited state manifolds relaxing after excitation.

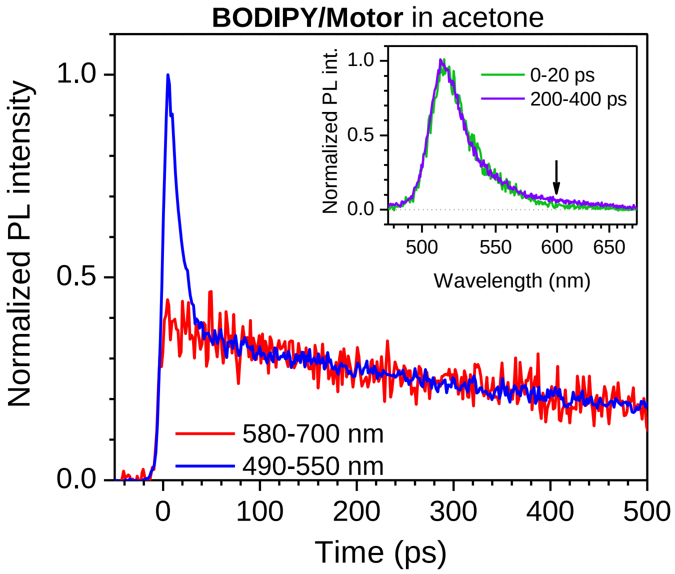

Supplementary Fig. 19. Time-resolved PL transients in short-wavelength (blue) and long-wavelength (red) regions of **BODIPY/Motor** in acetone under 400 nm excitation. The short-wavelength and long-wavelength PL transients were obtained by integrating the PL map in the spectral regions of 490–580 nm and 600–750 nm, respectively. Both transients were normalized to match their tails. The inset shows the transient spectra at the early-time (green) and long-time (violet) of **BODIPY/Motor** in acetone under 400 nm excitation. The black arrow depicts the difference in the long-wavelength region of the transient spectra at 0–20 ps and 200–400 ps times.

9.3. Solvent-polarity dependence on PL transients

Supplementary Fig. 20a shows time-resolved PL transients of **BODIPY/Motor** in various solvents with different polarity indexes (Supplementary Table 12) under 400 nm excitation. When the solvent polarity index increases, PL of **BODIPY/Motor** decays faster and exhibits more distinct bi-exponentiality at which the fastest decaying component becomes dominant. This result indicates a clear dependence of the solvent polarity on the PL transients of **BODIPY/Motor**. Supplementary Fig. 20b (blue dots) shows the correlation between the average PL lifetime of **BODIPY/Motor** and solvent polarity. With an increasing solvent polarity index (from toluene to acetonitrile), the average PL lifetime decreases exponentially from 2.6 ns to ~0.1 ns. No correlation was observed between the solvent viscosity and the PL lifetime of **BODIPY/Motor** (Supplementary Fig. 20b; red dots). Therefore, we conclude that the PL quenching of **BODIPY/Motor** can be controlled by using solvents with different polarity indexes.

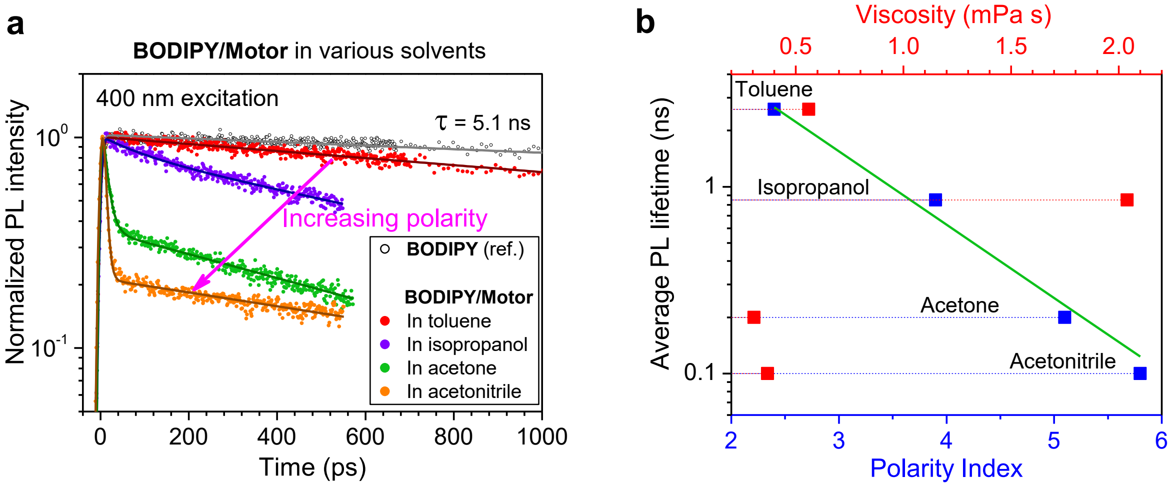

Supplementary Fig. 20. (**a**) Time-resolved PL transients (filled dots) of **BODIPY/Motor** in various solvents under 400 nm excitation. The open dots show the PL transient of bare **BODIPY** in toluene as a reference sample. The PL transients were obtained by integrating the PL maps in the spectral region between 490 nm and 600 nm. The solid lines show the best fits to exponential functions convoluted to the Gaussian apparatus function of the experimental data. The decaying times are summarized in Supplementary Table 12. The magenta arrow depicts the increase in solvent polarity. (**b**) Average PL lifetime of **BODIPY/Motor** as a function of solvent polarity index (blue dots) and viscosity (red dots). The polarity indexes and viscosity values of the solvents are summarized in Supplementary Table 12. The green line shows the best fit to a single-exponential function, $\tau_{ave} = \exp(-pt)$, with $p = 0.9$, to the average PL lifetime τ_{ave} as a function of the solvent polarity index p.

Supplementary Table 12. Fitting parameters for PL transients of BODIPY/Motor in different solvents and solvent polarity indexes and viscosity. Summary of the decay times and amplitude of the fits (using Equation (3)) for PL transients of **BODIPY/Motor** in different solvents under 400 nm excitation, average decay times (τ_{ave}), solvent polarity indexes and viscosity. The decay amplitudes of the fitting components were normalized to their sum.

Solvent	τ_1 (a_1)	τ_2 (a_2)	τ_{ave}[a]	Polarity Index[b]	Viscosity at 25 °C (mPa s)[c]
Toluene	–	2.6 ± 0.2 ns	2.6 ns	2.4	0.56
Isopropanol	100 ± 20 ps (0.16)	1 ± 0.2 ns (0.84)	0.85 ns	3.9	2.04
Acetone	12 ± 1 ps (0.75)	0.75 ± 0.1 ns (0.25)	0.2 ns	5.1	0.306
Acetonitrile	6 ± 1 ps (0.93)	1.3 ± 0.1 ns (0.07)	0.1 ns	5.8	0.369

[a] $\tau_{ave} = \tau_1 a_1 + \tau_2 a_2$

[b]Taken from Reference 19 for toluene and from Reference 20 for isopropanol, acetone and acetonitrile.

[c]Taken from Reference 21.

10. Transient absorption (TA)

Next, femtosecond transient absorption (TA) spectroscopy was used[4,22,23] to obtain further insights into excited state dynamics of **BODIPY/Motor.** Samples of **BODIPY/Motor** and two references, bare **Motor** and bare **BODIPY**, dissolved in acetone were prepared. With the acetone as the solvent, **BODIPY/Motor** shows a photoisomerization QY of 22% (Supplementary Table 6) and PLQY of 2.6% (Supplementary Table 4), implying that **BODIPY/Motor** favours the rotation function. The excitation wavelength of the TA measurements was fixed at 400 nm, which reaches the S_2 state of **BODIPY/Motor** (i.e., the absorption band of the motor core). The optical densities at 400 nm of bare **Motor**, bare **BODIPY** and **BODIPY/Motor** in acetone were 0.2, 0.01 and 0.04, which correspond to the molar concentration of $\sim 6 \times 10^{-5}$ M, $\sim 4 \times 10^{-4}$ M and $\sim 6 \times 10^{-5}$ M, respectively.

10.1. TA maps and transient spectra

Supplementary Fig. 21 shows TA maps and transient spectra of bare **Motor**, bare **BODIPY** and **BODIPY/Motor** under 400 nm excitation. The TA map of the bare **Motor** shows an intense, positive *ΔOD* region between 460 nm and 600 nm, which was observed in previous studies[23,24] and was assigned to excited state absorption (ESA) of bare **Motor**. A weak, positive *ΔOD* signal appears at a 460–500 nm region and at a ~45-ps pump-probe delay time (see clearer in Supplementary Fig. 21d). This 460–500 nm region lies in the absorption band of bare **Motor** in the metastable state[14] and therefore is assigned to the ground state absorption of **Motor** in the metastable state.

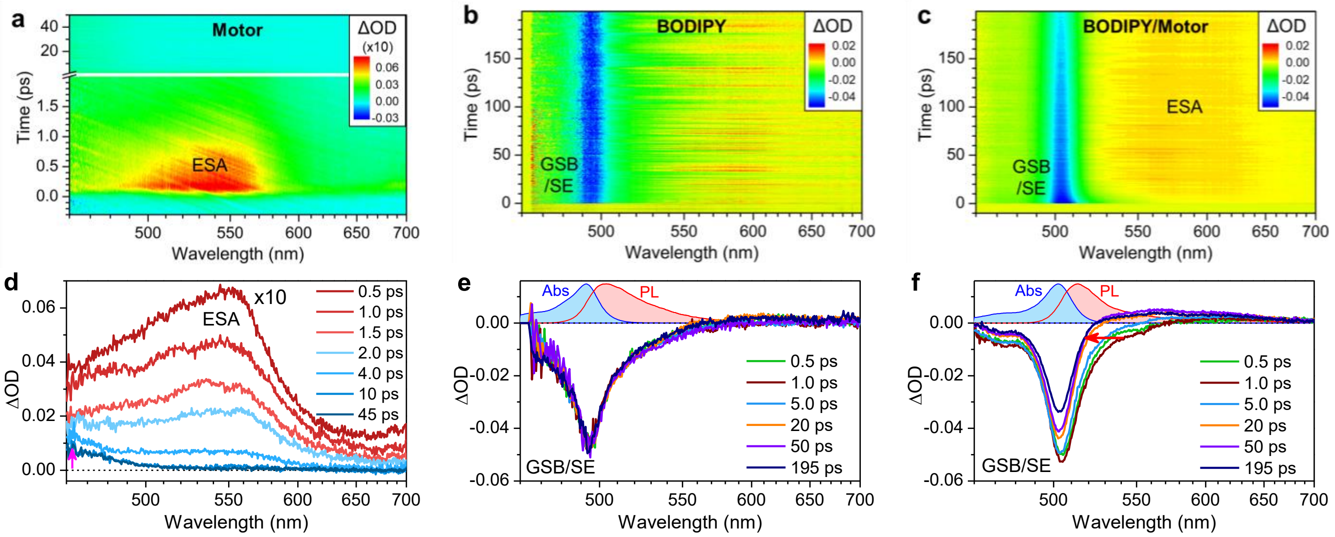

Supplementary Fig. 21. TA maps and spectra of Motor, BODIPY and BODIPY/Motor. TA maps (**a**–**c**) and transient spectra (**d**–**f**) of bare **Motor** (**a, d**), bare **BODIPY** (**b, e**) and **BODIPY/Motor** (**c, f**) under 400 nm excitation. GSB, SE and ESA stand for ground state bleaching, stimulated emission and excited state absorption, respectively. The blue and red shaded areas in **e**–**f** show steady-state absorption and PL spectra under 400 nm excitation. The TA spectra were obtained from the TA maps by averaging the spectral slices at different times listed in Supplementary Table 13. The magenta arrow in **d** depicts the formation of the photochemically generated metastable isomer; the red arrow in **f** depicts the transient spectral evolution. The molar concentrations of bare **Motor**, bare **BODIPY** and

BODIPY/Motor were set at $\sim 6 \times 10^{-5}$ M, $\sim 4 \times 10^{-4}$ M and $\sim 6 \times 10^{-5}$ M, respectively, with acetone as the solvent.

The TA map of bare **BODIPY** (Supplementary Fig. 21b) shows an intense, negative *ΔOD* region between ca. 465 nm and ca. 570 nm, which remains virtually unchanged throughout experimental 200 ps delay. This region lies in the absorption and PL band of BODIPY (Supplementary Fig. 21e; shaded areas) and therefore is assigned to ground state bleaching (GBS) and stimulated emission (SE) signals.

The TA map of **BODIPY/Motor** (Supplementary Fig. 21c) shows two regions of an intense, negative *ΔOD* between 460 nm and ca. 570 nm and a weak, positive *ΔOD* between ca. 520 nm and 700 nm. The former region lies in the absorption and PL band of the **BODIPY/Motor** (Supplementary Fig. 21f; shaded areas) and therefore is assigned to GSB and/or SE signals. The latter region has a positive *ΔOD* signal and thus is assigned to ESA of **BODIPY/Motor**. There is a blueshift from ~570 nm to ~520 nm of the zero-crossing point between GSB/SE and ESA signals, which occurs within the first 20 ps. This blueshift is consistent with the dynamical Stokes shift of in the mean PL energy of **BODIPY/Motor** in acetone (Supplementary Section 9.1) and indicates the excited state dynamic of **BODIPY/Motor** after excitation to form the metastable isomer with a timescale of ~10 ps.

Supplementary Table 13. Summary of the averaging ranges (in parentheses) for transient spectra in Supplementary Fig. 21d–f.

Bare **Motor**	Bare **BODIPY**	**BODIPY/Motor**
0.5 ps (0.3 − 0.7 ps)	0.5 ps (0.3 − 0.7 ps)	0.5 ps (0.3 − 0.7 ps)
1.0 ps (0.9 − 1.1 ps)	1.0 ps (0.8 − 1.2 ps)	1.0 ps (0.8 − 1.2 ps)
1.5 ps (1.4 − 1.6 ps)	10 ps (9 − 11ps)	10 ps (9 − 11ps)
2.0 ps (1.9 − 2.1 ps)	50 ps (48 − 52 ps)	50 ps (48 − 52 ps)
4.0 ps (3.8 − 4.2 ps)	100 ps (90 − 110 ps)	100 ps (98 − 102 ps)
10 ps (19 − 21 ps)	195 ps (190 − 200 ps)	195 ps (193 − 197 ps)
45 ps (43 − 47 ps)		

10.2. TA kinetic traces

Supplementary Fig. 22a shows TA kinetic traces of bare **Motor**, bare **BODIPY** and **BODIPY/Motor** at a probe wavelength of 510 nm under 400 nm excitation. This probe wavelength is close to the GSA/SE peaks of bare **BODIPY** and **BODIPY/Motor** (Supplementary Fig. 21e–f) and therefore the timescales of the GSA/SE signals of these compounds can be obtained.

The 510-nm TA trace of bare **Motor** shows a strong, positive ESA signal decaying to a long-lived offset (share of ~1%), which is attributed to the ground state absorption of the bare **Motor** in the metastable state (Supplementary Fig. 21a,d). The early time signal is fitted to a single exponential function with a lifetime τ~1.4 ps. This lifetime matches well with that from the previous studies[22,23] and was attributed to the timescale of S_1 excited state relaxation while moving the molecular system from the Frank–Condon region towards a CI with the S_0 state PES.

The 510-nm TA trace of bare **BODIPY** shows a strong, negative GSA/SE signal fitted to a single exponential function with a lifetime of 4.6 ns. In contrast, the 510-nm TA trace of **BODIPY/Motor** shows a strong, negative GSA/SE signal fitted to a bi-exponential function with lifetimes of τ_1~9 ps and τ_2~0.62 ns. These timescales of the GSA/SE signals of bare **BODIPY** and **BODIPY/Motor** match well with the PL lifetimes of the corresponding compounds (Supplementary Section 9.2), indicating the excited state quenching effect when a motor core is chemically attached to BODIPY.

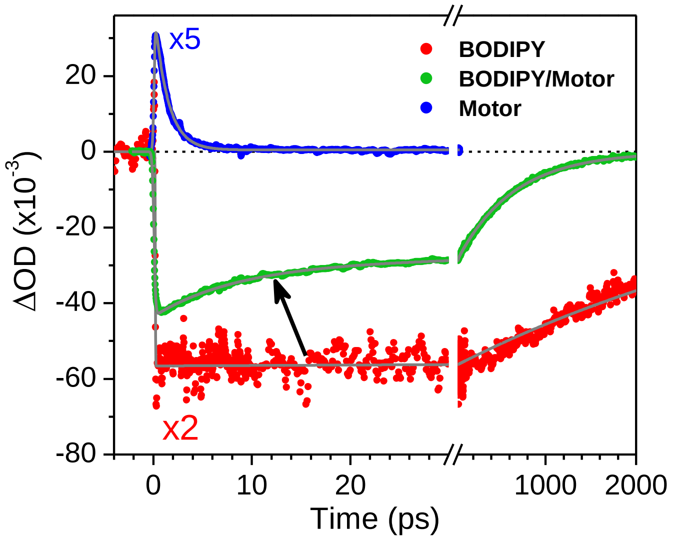

Supplementary Fig. 22. TA kinetic traces (filled dots) of bare **Motor** (blue), bare **BODIPY** (red) and **BODIPY/Motor** (green) in acetone at the probe wavelength of 510 nm under 400 nm excitation. The TA traces were obtained from a lock-in referenced photodiode selected the probe wavelength by a band-pass filter with the central wavelength of 508.5 nm and FWHM of 10 nm. The solid lines show the best fits to exponential functions convoluted to the Gaussian apparatus function of the experimental data. The fitting parameters are summarized in Supplementary Table 14. The black arrow depicts the excited

state quenching of BODIPY with the attachment of the motor core. The lower amplitude in the TA trace of **BODIPY** as compared to that of **BODIPY/Motor** is due to a blueshift in the GSB/SE peak (Supplementary Fig. 21e–f).

Supplementary Table 14. Fitting parameters for TA kinetic traces at 510 nm. Summary of the decay times and amplitudes of the fits (using Equation (3)) for 510-nm TA traces of bare **Motor**, bare **BODIPY** and **BODIPY/Motor** under 400 nm excitation. The sum of all amplitudes is normalized to unity.

Parameters	Bare **Motor**	Bare **BODIPY**	**BODIPY/Motor**
σ	110 ± 30 fs	100 ± 30 fs	150 ± 30 fs
a_1	0.99	–	0.32
τ_1	1.4 ± 0.1 ps	–	9 ± 1 ps
a_2	–	1	0.68
τ_2	–	4.6 ± 1.0 ns	0.62 ± 0.1 ns
a_0	0.01	–	–

Supplementary Fig. 23 shows TA kinetic traces of bare **Motor**, bare **BODIPY** and **BODIPY/Motor** at a probe wavelength of 550 nm under 400 nm excitation. This probe wavelength is close to the peaks of the ESA band of bare **Motor** and **BODIPY/Motor** (Supplementary Fig. 21d,f); therefore, the timescales of the ESA signals of these compounds could be directly obtained. Similar to above, the 550-nm TA trace of bare **Motor** shows a positive ESA signal decaying with a lifetime of $\tau \sim 1.4$ ps while bare **BODIPY** exhibits a negative GSB/SE signal with the lifetime of 5.5 ns. The 550-nm TA trace of **BODIPY/Motor** shows early-time (0–8 ps) the negative GSB/SE signal followed by a positive ESA signal at longer times.

Based on these findings, ultrafast TA spectroscopy revealed that **BODIPY/Motor** shows a longer timescale of the formation of the metastable isomer as compared to bare **Motor** (~10 ps *vs*. ~1.4 ps). According to SF-TDDFT calculations (Supplementary Section 8), this difference is explained by existence of an energy barrier on the S_1 state along the motor-rotation coordinate toward the CI point between the S_1 and S_0 state PESs ($CI_{S1/S0}$). The height of the energy barrier is lower for more polar solvents (Supplementary Fig. 15). Therefore, the $\tau_1 \sim 10$ ps time observed at both 510 nm and 550 nm probe wavelengths is assigned to the S_1 excited state relaxation while moving the system to the $CI_{S1/S0}$ point. In bare **Motor**, the barrier does not exist which results in much faster relaxation time.

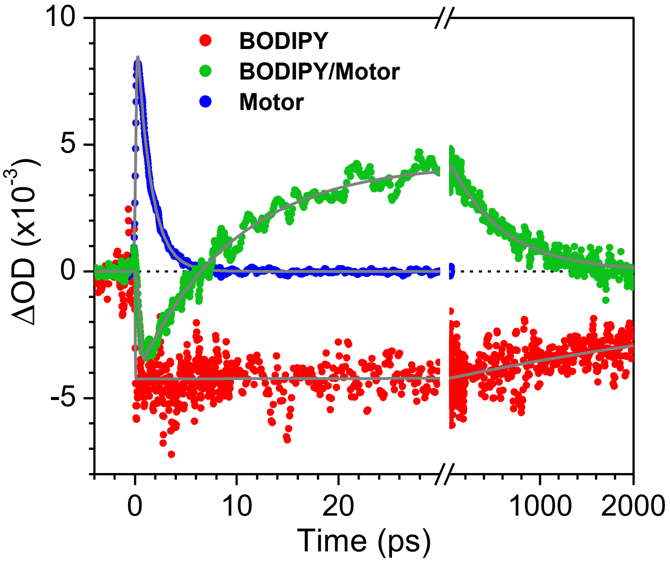

Supplementary Fig. 23. TA kinetic traces (filled dots) of bare **Motor** (blue), bare **BODIPY** (red) and **BODIPY/Motor** (green) in acetone at the probe wavelength of 550 nm under 400 nm excitation. The TA traces were obtained from a lock-in referenced photodiode selected the probe wavelength by a band-pass filter with the central wavelength of 550 nm and full width at half maximum of 10 nm. The solid lines show the fits to exponential functions convoluted to the Gaussian apparatus function of the experimental data. The fitting parameters are summarized in Supplementary Table 15.

Supplementary Table 15. Fitting parameters for TA kinetic traces at 550 nm. Summary of the decay times and amplitudes of the fits (using Equation (3)) for 550-nm TA traces of bare **Motor**, bare **BODIPY** and **BODIPY/Motor** under 400 nm excitation. The sum of all amplitudes is normalized to unity.

Parameters	Bare **Motor**	Bare **BODIPY**	**BODIPY/Motor**
σ	120 ± 30 fs	100 ± 30 fs	200 ± 30 fs
a_1	1	–	0.65*
τ_1	1.4 ± 0.1 ps	–	10 ± 1 ps
a_2	–	1	0.35
τ_2	–	5.5 ± 1.0 ns	0.6 ± 0.2 ns

(*) Negative amplitude

11. Difference absorption (*ΔOD*) spectra

11.1. *ΔOD* spectra of BODIPY/Motor

Supplementary Section 6 has demonstrated the rotation functionality of **BODIPY/Motor** in various solvents under the excitation wavelength with 505 nm green light. To obtain further insight into the mechanism that drives the motor rotation with green light, difference absorption (*ΔOD)* spectroscopy experiments were performed.

Supplementary Fig. 24 shows *ΔOD* spectra of a sample of **BODIPY/Motor** dissolved in toluene measured at different times after 505 nm excitation. There is a strong *ΔOD* change in the absorption domain of the motor core moiety with the isosbestic point at ~415 nm, which is in line with the UV/Vis spectral change (Supplementary Fig. 10b-12b). Apart from the change in the absorption domain of the motor core moiety, there is also a weak change in the absorption domain of the BODIPY moiety (ca. 460–520 nm). If the motor core and BODIPY moieties did not interact to each other so that they could function as individual molecules, then the *ΔOD* change in the absorption domain of the BODIPY moiety should not have occurred. Note that the *ΔOD* change caused by temperature fluctuation during the experiment is ruled out because this change was ~50 times lower than the *ΔOD* change observed in **BODIPY/Motor** under 505 nm irradiation (see Supplementary Section 11.3).

The change in *ΔOD* spectra was fully vanished after ~25 min, which is in line with the thermal recovery of the absorption spectrum of **BODIPY/Motor** after irradiation with 395-nm light (Supplementary Fig. 10c-12c). The kinetic traces of *ΔOD* at 455 nm (which lies in the motor core absorption) and 515 nm (which lies in the BODIPY absorption) show a similar lifetime of $\tau \sim 5.1 - 5.5\ \mathrm{min}$, which is in line with the results of Eyring plot analysis of **BODIPY/Motor** after 395-nm irradiation (Supplementary Section 6). The similar lifetime indicates that the *ΔOD* change in the BODIPY moiety is associated with the photoisomerization, which is exhibited by the *ΔOD* change of the motor moiety around the isosbestic point at ~415 nm.

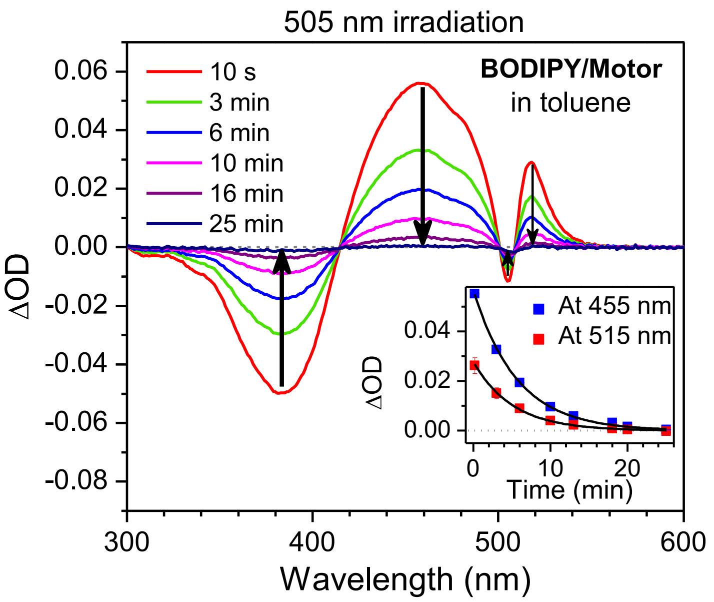

Supplementary Fig. 24. Difference absorption (*ΔOD*) spectra of **BODIPY/Motor** in toluene measured at different times between 10 s and 25 min after irradiation with 505-nm light. The light source of irradiation was provided by a 505-nm LED with the output power of ~10 mW; the irradiation time was 5 min. The black arrows depict the thermal recovery of stable isomers via THI from metastable isomers. The inset shows the decreases of the positive *ΔOD* peaks at 455 nm (blue) and 515 nm (red) over time together with the exponential fit (black lines), $y = A \cdot \exp\,(-t/\tau)$ with a lifetime of $\tau = 5.5 \pm 0.1$ min and $\tau = 5.1 \pm 0.3$ min, respectively. Data for the positive *ΔOD* peak at 455 nm and 515 nm were obtained by averaging *ΔOD* values in $450 - 460$ nm and $513 - 518$ nm ranges, respectively; the error bars refer to standard deviation. The molar concentration of **BODIPY/Motor** was set at $\sim 1 \times 10^{-5}$ M.

11.2. Is the BODIPY/Motor rotation driven via a triplet pathway under 505-nm excitation?

One of the possibilities to drive motor rotation by the 505-nm green light is to proceed to the formation of the metastable isomer via a triplet instead of a singlet excited state of the motor. This approach has been reported by Cnossen *et al.*[25] who demonstrated the motor functionality with green light ($\lambda = 530$ nm) using a triplet sensitizer based on palladium porphyrin. The triplet excited state of the motor was activated either by intra or inter-molecular energy transfer from the sensitizer, which was obtained by a chemical attachment or mixture of two functional molecules in solution, respectively. This study suggests a plausible hypothesis that the motor rotation of **BODIPY/Motor** under 505-nm irradiation could occur via a triplet channel in which the triplet excited state of the motor is activated by intra-molecular energy transfer from the BODIPY moiety.

To examine the hypothesis of driving motor rotation via the triplet channel, we raise two questions related to the underlying mechanism: (i) Can the **BODIPY/Motor** function via the *triplet* pathway? (ii) Is the triplet state really *populated* in **BODIPY/Motor** under 505 nm excitation? The former question can be addressed by performing *ΔOD* spectroscopy experiments of a solution sample of **BODIPY/Motor** mixed with a triplet sensitizer. In a similar

mechanism reported by Cnossen *et al.*[25], the triplet excited state of **BODIPY/Motor** is activated by inter-molecular energy transfer from the triplet excited state of the sensitizer to **BODIPY/Motor**.

If the triplet excited state of **BODIPY/Motor** is populated (by e.g. intra-molecular energy transfer or intersystem crossing), it can be probed by adding a triplet quencher to form a competing pathway to depopulate the triplet excited state. Because of this quenching pathway, motor rotation via accessing the triplet channel is expected to be weaker so that the change in ΔOD spectra would be lower as compared to that of the sample without the triplet quencher.

Two triplet molecules of Pt(II)-tetraphenyl-tetrabenzoporphyrin (**PtTPBP**) and palladium(II) octabutoxyphthalocyanine (**PdPc(OBu)$_8$**) were identified as the excellent choices because the energies of the T_1 excited state of **PtTPBP** and **PdPc(OBu)$_8$** are above and below that of **BODIPY/Motor**, respectively. Specifically, **PtTPBP** and **PdPc(OBu)$_8$** have calculated triplet energies E_{T1}~1.74 eV and E_{T1}~1.18 eV, respectively, which are higher and lower than the calculated triplet energy of **BODIPY/Motor** E_{T1}~1.63 eV (Supplementary Table 16). The difference in the triplet energies between the triplet molecules and **BODIPY/Motor** facilitates triplet–triplet energy transfer (TTET) in two different directions: (i) from **PtTPBP** to **BODIPY/Motor** and (ii) from **BODIPY/Motor** to **PdPc(OBu)$_8$**. Therefore, **PtTPBP** and **PdPc(OBu)$_8$** play as triplet sensitizer and quencher, respectively, when mixing them with **BODIPY/Motor** in solution.

Supplementary Table 16. Vertical excitation energies of T_1 and S_1 excited states using TDDFT(CAM-B3LYP)/def2-SVP in toluene.

Compounds	T_1 (eV)	S_1 (eV)
**BODIPY/Motor**	1.63	3.12
**PtTPBP**	1.74	2.38
**PdPc(OBu)$_8$**	1.18	2.06

11.2.1. Absorption spectra of mixtures of BODIPY/Motor and triplet molecules

Supplementary Fig. 25b shows absorption spectra of pure **BODIPY/Motor**, **BODIPY/Motor** mixed with **PtTPBP** and with **PdPc(OBu)$_8$** in degassed toluene, together with reference spectra of **PtTPBP** and **PdPc(OBu)$_8$** in degassed toluene. **PtTPBP** and **PdPc(OBu)$_8$** show strong absorption in the red and near-infrared (NIR) wavelength regions, respectively, which are beyond the absorption of **BODIPY/Motor**. Both triplet molecules show a negligibly weak absorption in the green wavelength region, which lies in the absorption band of **BODIPY/Motor**. Therefore, **BODIPY/Motor**, **PtTPBP** and **PdPc(OBu)$_8$** can be selectively excited with different excitation wavelengths of the light source, such as green, red or NIR light.

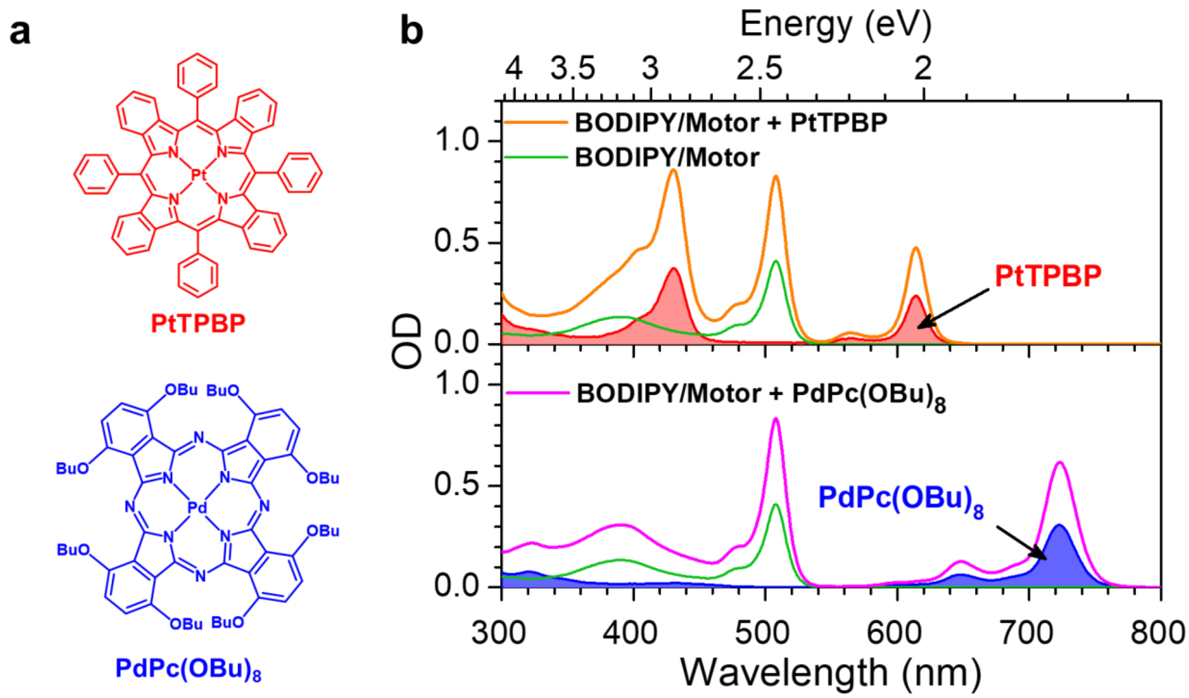

Supplementary Fig. 25. (**a**) Chemical structures of **PtTPBP** and **PdPc(OBu)$_8$**. (**b**) Absorption spectra of pure **BODIPY/Motor** (green), **BODIPY/Motor** mixed with **PtTPBP** (orange) and **BODIPY/Motor** mixed with **PdPc(OBu)$_8$** (magenta) in degassed toluene. The red and blue shaded areas depict reference absorption spectra of pure **PtTPBP** and pure **PdPc(OBu)$_8$** in degassed toluene. For the sake of clarity, the data of pure **BODIPY/Motor**, pure **PtTPBP** and pure **PdPc(OBu)$_8$** were divided by 2. The molar concentration of **BODIPY/Motor** and **PtTPBP** (or **PdPc(OBu)$_8$**) in the mixture are $\sim 1 \times 10^{-5}$ M and $\sim 0.33 \times 10^{-5}$ M, respectively.

11.2.2. ΔOD spectra of mixture samples under 505-nm and 635-nm irradiations

Supplementary Fig. 26a shows *ΔOD* spectra of pure **BODIPY/Motor** and **BODIPY/Motor** mixed with **PtTPBP** in degassed toluene after irradiation with 505-nm and 635-nm light, which selectively excites **BODIPY/Motor** and **PtTPBP**, respectively. After 505 nm irradiation, the *ΔOD* spectrum of **BODIPY/Motor** mixed with **PtTPBP** shows a clear change identical with the reference spectrum of pure **BODIPY/Motor**. This result indicates that **BODIPY/Motor** in a mixture with the triplet sensitizer **PtTPBP** maintains its rotation function under irradiation with 505-nm green light (Supplementary Fig. 26b; upper graph).

After 635 nm irradiation, as expected, the reference sample of pure **BODIPY/Motor** shows no change in the *ΔOD* spectrum because the irradiation wavelength is outside the absorption region of **BODIPY/Motor**. In contrast, **BODIPY/Motor** mixed with **PtTPBP** after 635 nm irradiation shows a clear change in *ΔOD* with the shape similar to that after 505 nm irradiation. This result indicates that **BODIPY/Motor** in a mixture with **PtTPBP** exhibits its rotation function under irradiation with 635-nm red light. As was demonstrated by Cnossen *et al.*[25], the underlying mechanism of this motor functionality was attributed to intermolecular energy transfer from **PtTPBP** (after excitation and S_1–T_1 intersystem crossing) to the low-lying triplet excited state of **BODIPY/Motor** to drive motor rotation[25]. Therefore, we conclude that once the triplet excited state of **BODIPY/Motor** is populated, motor rotation might occur via accessing the triplet pathway (Supplementary Fig. 26b; lower graph). Furthermore, the molecular diffusion length of **PtTPBP** in the triplet excited state is estimated as ~250 nm (Supplementary Section 11.2.3), which is much longer than the average distance between **BODIPY/Motor** and **PtTPBP** molecules (d~50 nm, using Equation (6)). This result indicates that the intermolecular energy transfer from **PtTPBP** to **BODIPY/Motor** is likely mediated by a molecular-diffusion process and further supports above conclusion.

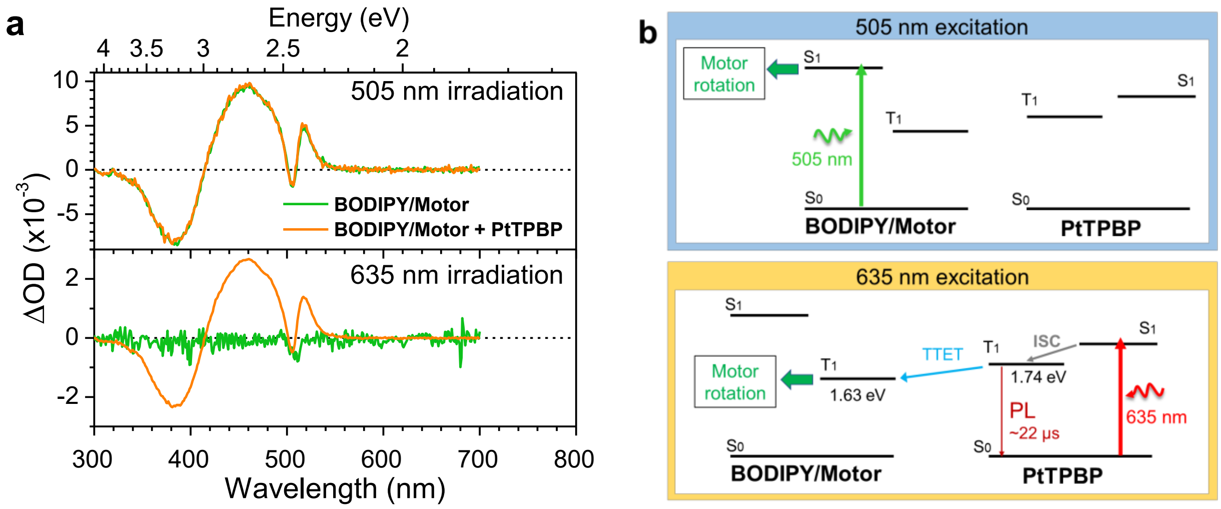

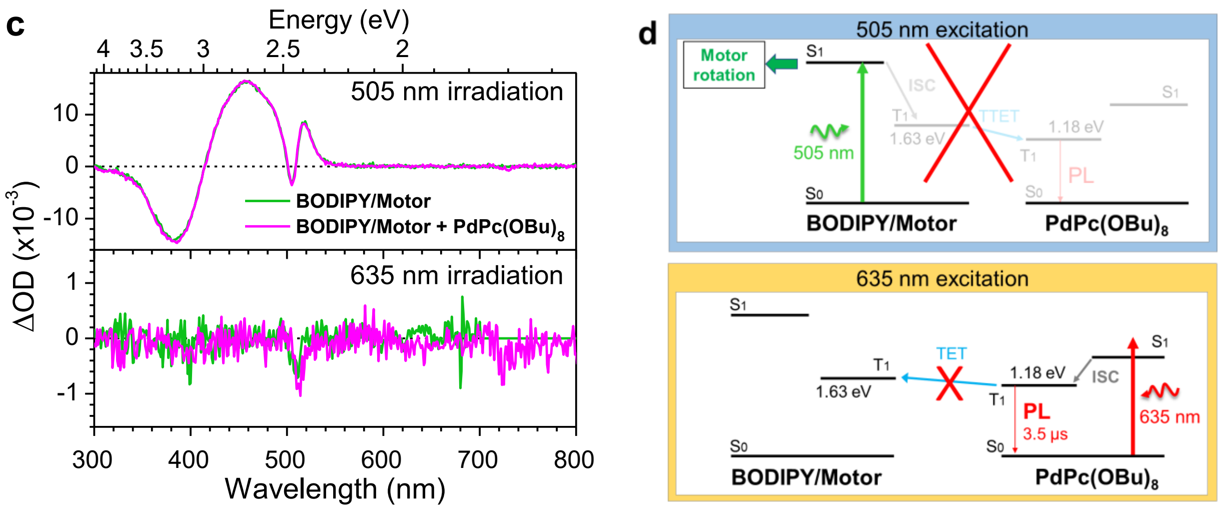

Supplementary Fig. 26. (**a, c**) Difference absorption (*ΔOD*) spectra of pure **BODIPY/Motor** (green), **BODIPY/Motor** mixed with **PtTPBP** (orange) and **BODIPY/Motor** mixed with **PdPc(OBu)$_8$** (magenta) in degassed toluene measured at 10 s after irradiation with 505 nm (upper graphs) and 635 nm (lower graphs) LED light. The irradiation time for 505 nm and 635 nm irradiations were fixed at 1 min and 5 min, respectively. The molar concentrations of **BODIPY/Motor** and **PtTPBP** (or **PdPc(OBu)$_8$**) in mixtures were $\sim 1 \times 10^{-5}$ M and $\sim 0.33 \times 10^{-5}$ M, respectively. (**b**, **d)** Illustrations of excited-state energy levels of **BODIPY/Motor**, **PtTPBP** and **PdPc(OBu)$_8$** under 505 nm and 635 nm excitations. ISC and TTET stand for intersystem crossing and triplet–triplet energy transfer, respectively. The T_1 state energies of the studied compound were obtained from SF-TDDFT calculations (Supplementary Table 16). The T_1 triplet lifetime was adapted from References 26 for **PtTPBP** and References 27 for **PdPc(OBu)$_8$**.

Next, we address the question that if the triplet excited state of **BODIPY/Motor** is really populated under 505 nm excitation. To do so, *ΔOD* spectroscopy experiments of a sample of **BODIPY/Motor** mixed with the **PdPc(OBu)$_8$** triplet quencher and a reference sample of pure **BODIPY/Motor** in degassed toluene were performed.

Supplementary Fig. 26c shows *ΔOD* spectra of **BODIPY/Motor** mixed with **PdPc(OBu)$_8$** in degassed toluene after irradiation with 505-nm and 635-nm light, which selectively excites **BODIPY/Motor** and **PdPc(OBu)$_8$**, respectively. As expected for the lower T_1 energy of **PdPc(OBu)$_8$** than that of **BODIPY/Motor** (Supplementary Fig. 26d; low graph), the *ΔOD* spectrum of **BODIPY/Motor** mixed with **PdPc(OBu)$_8$** shows no change under 635 nm irradiation. Note that the molecular diffusion length of **PdPc(OBu)$_8$** in the triplet excited state is estimated as ~80 nm (Supplementary Section 11.2.3), which remains longer than the average distance between **BODIPY/Motor** and **PdPc(OBu)$_8$** molecules ($d \sim 50$ nm, using Equation (6)). Therefore, **PdPc(OBu)$_8$** is likely to approach **BODIPY/Motor** within the triplet lifetime of **PdPc(OBu)$_8$**.

In contrast to *ΔOD* spectra under 635 nm irradiation, the sample of **BODIPY/Motor** mixed with **PdPc(OBu)$_8$** shows a change in the *ΔOD* spectrum after 505 nm irradiation, which is perfectly identical with that of pure **BODIPY/Motor**. If the triplet excited state of **BODIPY/Motor** had been populated and the subsequent TTET to **PdPc(OBu)$_8$** had been efficient, the amount of the metastable isomer must have been reduced because of losses to the **PdPc(OBu)$_8$** quencher (Supplementary Fig. 26d; upper graph). Obviously, this is not the case as there is no reduction in *ΔOD* of **BODIPY/Motor** mixed with **PdPc(OBu)$_8$** after 505 nm irradiation, compared to the reference sample of pure **BODIPY/Motor**.

The conclusion from these experiments is the following. The motor rotation can be activated via its triplet state; however, the triplet state does not appear to be involved when the functionalized motor is excited at 505 nm. Therefore, the scenario that the functionality of **BODIPY/Motor** is activated via the pathway of the triplet excited state, can be reasonably ruled out.

11.2.3. Calculation of molecular diffusion lengths in solution

To examine if energy transfer from a sensitizer (e.g. **PtTPBP**, **PdPc(OBu)$_8$** or **BODIPY**) to bare **Motor** in a mixture solution could be mediated by molecular diffusion, we calculated a diffusion length of the relevant molecules in their excited states. If this diffusion length is larger than an average distance between the sensitizer and **Motor** molecules (which could be estimated from the molar concentrations of constituents in solution, see below), molecular-diffusion mediated energy transfer would likely occur.

The diffusion length of a molecule in the excited state in solution can be calculated using the following equation[28]:

$$L_D = \sqrt{6D\tau} \quad (4)$$

where D is the diffusion coefficient of the molecule in solution and τ is the lifetime of the molecule in the excited state. The diffusion coefficient of the molecule in solution can be estimated from the Stokes–Einstein equation[29,30] as follows:

$$D = \frac{k_B T}{6\pi\eta r} \quad (5)$$

where $k_B \approx 1.38 \times 10^{-23}$ J K^{-1} is Boltzmann's constant, T is the absolute temperature, η is the dynamic viscosity of the solvent (for toluene at 25 °C, $\eta = 0.56$ mPa s) and r is the radius of the molecule.

The average distance ($\langle d \rangle$; in nm) between the sensitizer and **Motor** in a mixture solution is estimated from the molar concentrations of constituents in the solution using the following equation:

$$\langle d \rangle = \sqrt[3]{\frac{10^{24}}{N_A \cdot c_M}} \quad (6)$$

where $N_A \approx 6.02 \times 10^{-23}\ mol^{-1}$ is Avogadro constant and c_M is the sum of the concentrations of the sensitizer and **Motor** in the mixture. The factor of 10^{24} results from the unit conversion: $1\ \mathrm{L} = 10^{24}\ \mathrm{nm}^3$.

Supplementary Table 17. Summary of excited-state lifetimes (τ), radius of the molecule (r), diffusion coefficients (D; using Equation (5)) and lengths (L_D; using Equation (4)) of **PtTPBP** and **PdPc(OBu)$_8$** in solution and average distances ($\langle d \rangle$; using Equation (6)) between the sensitizer (**PtTPBP**, **PdPc(OBu)$_8$** or **BODIPY**) and bare **Motor** in mixture solutions.

Compounds	τ	r (nm)[a]	D (nm^2 ps^{-1})	L_D (nm)	$\langle d \rangle$ (nm)
**PtTPBP**	22 µs [b]	0.8	7.7×10^{-4}	250	50
**PdPc(OBu)$_8$**	3.5 µs [c]	1.3	3.9×10^{-4}	80	50
**BODIPY**	5.5 ns[d]	0.5	1.3×10^{-3}	5	44

[a] Estimated from a half of the largest size of the molecular structure

[b] Lifetime of the triplet excited state of **PtTPBP** in THF reported previously in Reference 26.

[c] Lifetime of the triplet excited state of **PdPc(OBu)₈** in benzene reported previously in Reference 27.

[d] Lifetime of the singlet excited state of **BODIPY** in toluene obtained from the time-resolved PL measurements (Supplementary Table 11)

PtTPBP and **$PdPc(OBu)_8$** molecules dissolved in toluene show long molecular-diffusion lengths of 250 nm and 80 nm, respectively, in their triplet states. These values are higher than the average distance between the molecules and bare **Motor** ($\langle d \rangle$~50 nm) in the solutions (with molar concentration of $\sim 1 \times 10^{-5}$ M for bare **Motor** and of $\sim 0.33 \times 10^{-5}$ M for the triplet molecule). Therefore, molecular-diffusion mediated energy transfer would likely occur in these mixtures. In contrast to the triplet molecules, **BODIPY** in the single excited state has a short $L_D = 5$ nm, which is much smaller than the average distance between the BODIPY molecule and **Motor** (d~44 nm) in a mixture solution. Therefore, molecular-diffusion mediated energy transfer is unlikely to occur in the mixture of bare **BODIPY** and bare **Motor**.

11.3. *ΔOD* spectra of a BODIPY+Motor mixture

Supplementary Fig. 27 shows *ΔOD* spectra of bare **Motor**, **BODIPY+Motor** and **BODIPY/Motor** in toluene after 505 nm irradiation. No change in *ΔOD* of the bare **Motor** was observed, indicating that bare **Motor** does not function under 505 nm irradiation. The **BODIPY+Motor** mixture shows a very weak change in *ΔOD* (~40 times lower than **BODIPY/Motor**). This result demonstrates that the motor can function under 505 nm irradiation when BODIPY is mixed with the motor in a solution.

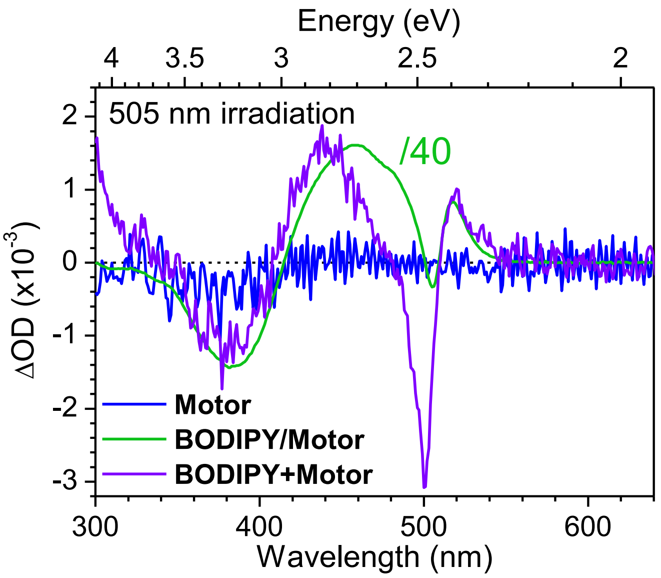

Supplementary Fig. 27. Difference absorption (*ΔOD*) spectra of bare **Motor** (blue), **BODIPY/Motor** (green) and **BODIPY+Motor** (violet) in toluene measured at 10 s after irradiation with 505-nm LED

light. The output power of LED was ~10 mW. The molar concentrations of **Motor**, **BODIPY/Motor** and the motor and BODIPY in the **BODIPY+Motor** mixture were set at $\sim 1 \times 10^{-5}$ M.

A plausible assumption is that the excitation energy is dissipated partly into thermal energy which raises the local temperature of the entire sample volume. This increase in temperature after irradiation could cause a change in the *ΔOD* spectrum of **BODIPY+Motor** with a spectral shape similar to that resulted by photoisomerization. The temperature change dT of a solution reservoir with the thermal energy dQ added into the reservoir can be derived from the following equation[31]:

$$dT = dQ/(m \cdot c_p) \qquad (7)$$

where $m = 1.72$ g and $c_p = 1.7\ \mathrm{J \cdot g^{-1} \cdot K^{-1}}$ are the mass and specific heat capacity of the solvent (toluene), respectively. $dQ = 0.13$ J is obtained by the product of the entire irradiation energy of the light source, the light absorption of **BODIPY+Motor** and the fraction of the light absorption to thermal energy (without losing to PL) as given in the following equation:

$$dQ = P \cdot t \cdot (1 - 10^{-OD}) \cdot (1 - \eta) \qquad (8)$$

where $P = 10$ mW and $t = 5$ min are the irradiation power and time of the light source, respectively, $OD = 0.87$ is the optical density at the irradiation wavelength of the **BODIPY+Motor** sample, $\eta \sim 95\%$ is the PL quantum yield of bare **BODIPY** in toluene[9]. By substituting all numerical values into Equations (7) and (8), we obtained $dT \sim 0.05$ K (or 0.05 ℃).

Then we performed *ΔOD* spectroscopy measurements at different temperatures of the **BODIPY+Motor** sample, but no irradiation was used. The temperature of the sample holder was varied from 20 °C to 40 °C. *ΔOD* spectra were obtained by subtracting the initial absorption spectrum at 20 °C from the spectra measured at higher temperatures of the sample.

Supplementary Fig. 28 shows *ΔOD* spectra of **BODIPY+Motor** in toluene with the increase in temperature (*ΔT*) of 1 °C. There is a noticeable change in *ΔOD* at ~500 nm, which is linearly dependent on *ΔT* (inset of Supplementary Fig. 28). However, the amplitude of the signal with temperature change is a factor of 20 lower than the change in the absorption spectrum after irradiation with green light (*violet curve*). Besides, the spectral changes are confined in the spectral region of BODIPY in the **BODIPY+Motor** mixture; no changes in the region of “motor rotation” (350–450 nm) were detected. This clearly demonstrates that changing the sample temperature (by excitation energy dissipated to thermal energy and/or fluctuation of the sample temperature) does not lead to isomerization of the motor.

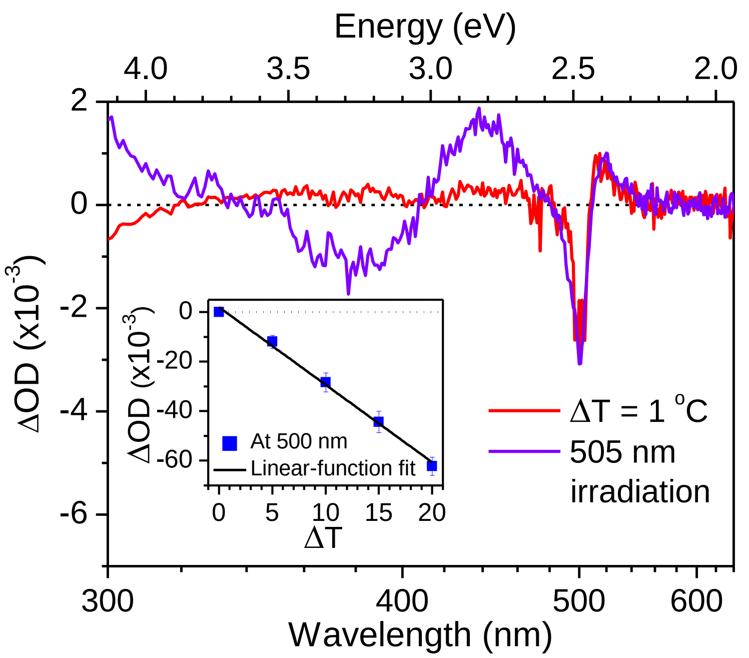

Supplementary Fig. 28. Difference absorption (ΔOD) spectra of **BODIPY+Motor** in toluene with an increase in the temperature $\Delta T = 1$ ºC (red) and after irradiation with 505-nm LED light for 5 min (violet). The initial temperature of the sample was 20 ºC. The spectrum with $\Delta T = 1$ ºC was obtained from the experimental ΔOD spectrum with $\Delta T = 5$ ºC and divided by 5. Note that the estimated temperature rise in the focal volume was ~0.05 ºC, i.e., a factor of 20 lower than for the red spectrum. The molar concentrations of the motor and BODIPY in **BODIPY+Motor** were set to be identical at $\sim 1 \times 10^{-5}$ M. The inset shows the dependence of ΔOD at 500 nm on ΔT and the black line shows the fit to a linear function. The error bars refer to standard deviation.

Having established that the *ΔOD* change of the **BODIPY+Motor** spectrum is not due to the temperature change after irradiation, we move to the next possible scenario, namely diffusion-mediated energy transfer. The molecular diffusion length of BODIPY in the singlet excited state is estimated as ~5 nm (Supplementary Section 11.2.3), which is approximately one order of magnitude shorter than the average distance between BODIPY and motor molecules ($d \sim 44\ \mathrm{nm}$, using Equation (6)). Therefore, BODIPY in the singlet excited state likely decays to its ground state before approaching the motor molecules. However, if BODIPY is populated to its triplet excited state (a maximum yield of 5% as the fluorescence QY is 95%[9]), a longer lifetime and thus a longer diffusion length of BODIPY in the excited state can be obtained. If this is the case, motor rotation in the **BODIPY+Motor** mixture occurs via the triplet pathway after TTET from BODIPY. Therefore, even though we could speculate that the motor in the **BODIPY+Motor** mixture functions under irradiation with 505-nm green light via accessing the triplet pathway, this channel is much less efficient than the one arising from the synergetic effects, i.e., direct excitation of the **BODIPY/Motor** system.

12. Circular dichroism spectroscopy

Circular dichroism (CD) spectroscopy measurements of **BODIPY/Motor** were performed after the enantiomers (*R* and *S*) were successfully separated by supercritical fluid chromatography (SFC). Without SFC of the motor, the change in the CD spectra cannot be observed because of the coexistence of both *R* and *S* enantiomers in solution, e.g. bare motor core (Supplementary Fig. 29e).

The samples of **BODIPY/Motor** with the *R* and *S* enantiomers (hereafter, denoted as ***R***- and ***S*-BODIPY/Motors**, respectively) were prepared by dissolving them in acetone (0.9×10^{-5} M). The CD spectra of ***R***- and ***S*-BODIPY/Motors** were measured in three different states: before irradiation, 10 secs and 10 min after reaching the PSS by irradiation with 395-nm light (Supplementary Fig. 29a–c). In the latest state, the sample was kept at 30 ^{o}C in dark to ensure all metastable isomers progressing to their second, identical stable isomers via THI. The CD spectra of ***R***- and ***S*-BODIPY/Motors** before irradiation (Supplementary Fig. 29a) shows a mirror image in circular dichroism, indicating the enantiomeric couple of **BODIPY/Motor** at the stable state. Interestingly, the spectra exhibit two clear CD peaks at ~390 nm and ~500 nm, which lies on the absorption region of the motor core at the stable state and BODIPY moieties, respectively. This result indicates that chirality from the chiral motor core is transferred to the achiral BODIPY moiety. Performing similar experiments on bare **BODIPY**, we observe no change in the CD spectra (Figure S12d), indicating that **BODIPY** is an achiral chromophore, thus supporting the above conclusion.

After irradiation to reach the PSS, the CD spectra of ***R***- and ***S*-BODIPY/Motors** (Supplementary Fig. 29b) shows the opposite CD signs as compared to those of the spectra before irradiation. This inversion of the CD sign is attributed to the inversion of the helical chirality at the motor core, which has been observed previously[14]. The consequence of the helical chirality inversion is observed, which the CD peak associated to the absorption of the motor core is redshifted to, ca., 430 nm. In contrast, the CD absorption maximum associated to the absorption of BODIPY remains at ~500 nm but opposite in sign, indicating that this CD peak can be exploited as a visible-wavelength reporter of the chirality inversion in **BODIPY/Motor**.

Finally, the CD spectra of ***R***- and ***S*-BODIPY/Motors** 10 min after reaching the PSS and keeping them at 30 ^{o}C in dark (Supplementary Fig. 29c) recover to be similar to those before irradiation. This result is in line with the UV/Vis spectral change (Supplementary Section 6) and NMR (Supplementary Section 4).

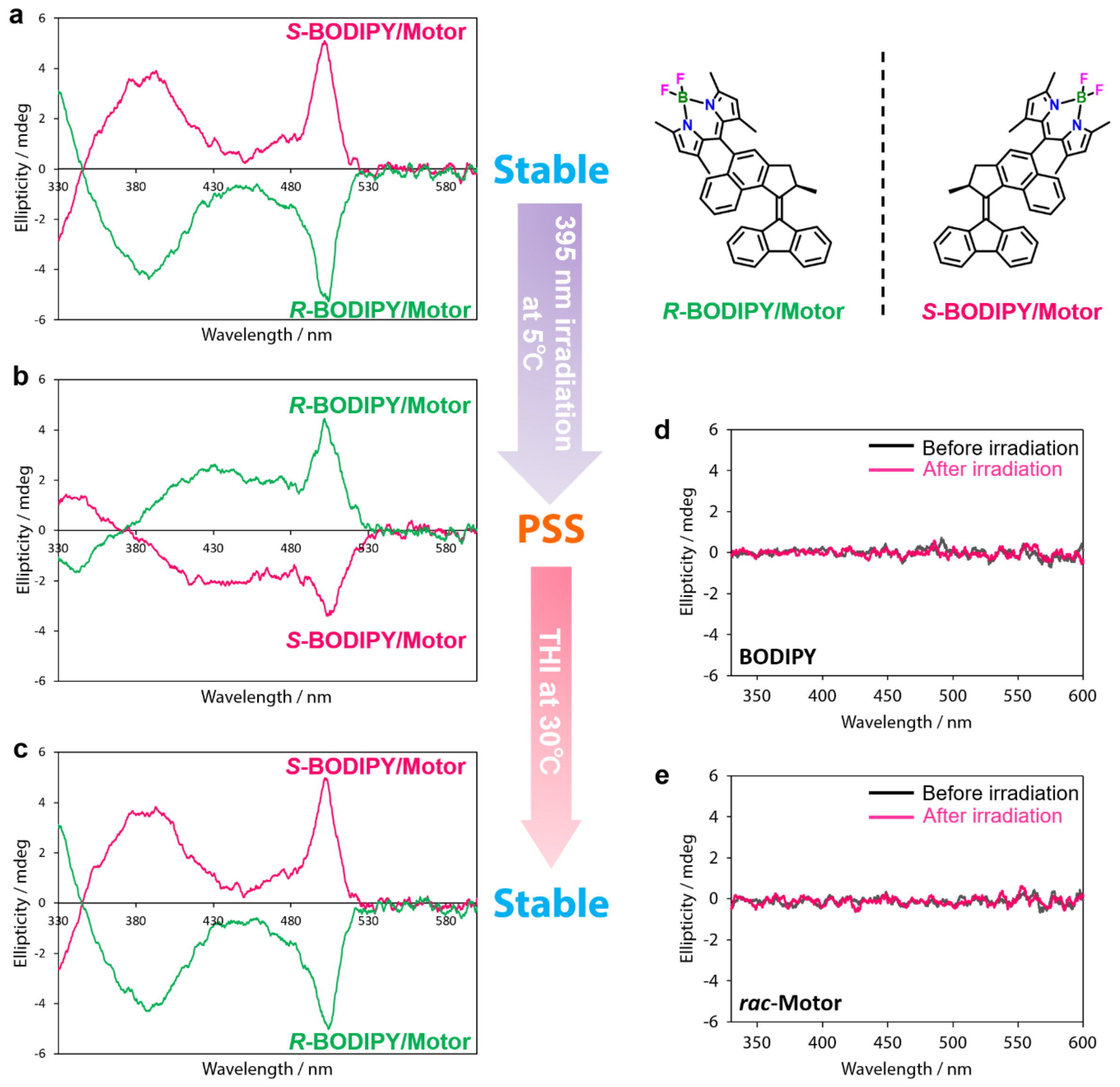

Supplementary Fig. 29. (**a**–**b**) Circular dichroism (CD) spectra of ***R***- and ***S*****-BODIPY/Motors** before irradiation (**a**), 10 secs (**b**) and 10 min (**c**) after reaching the PSS by irradiation with a 395-nm LED in acetone. The samples before irradiation and 10 sec after reaching the PSS was kept at 5°C and afterward kept to 30°C for 10 min. (**c**–**d**) CD spectra of **BODIPY** (**d**) and racemic ***rac*****-Motor** (**e**) in acetone before and after irradiation with a 395-nm LED.

13. NMR spectra for characterization

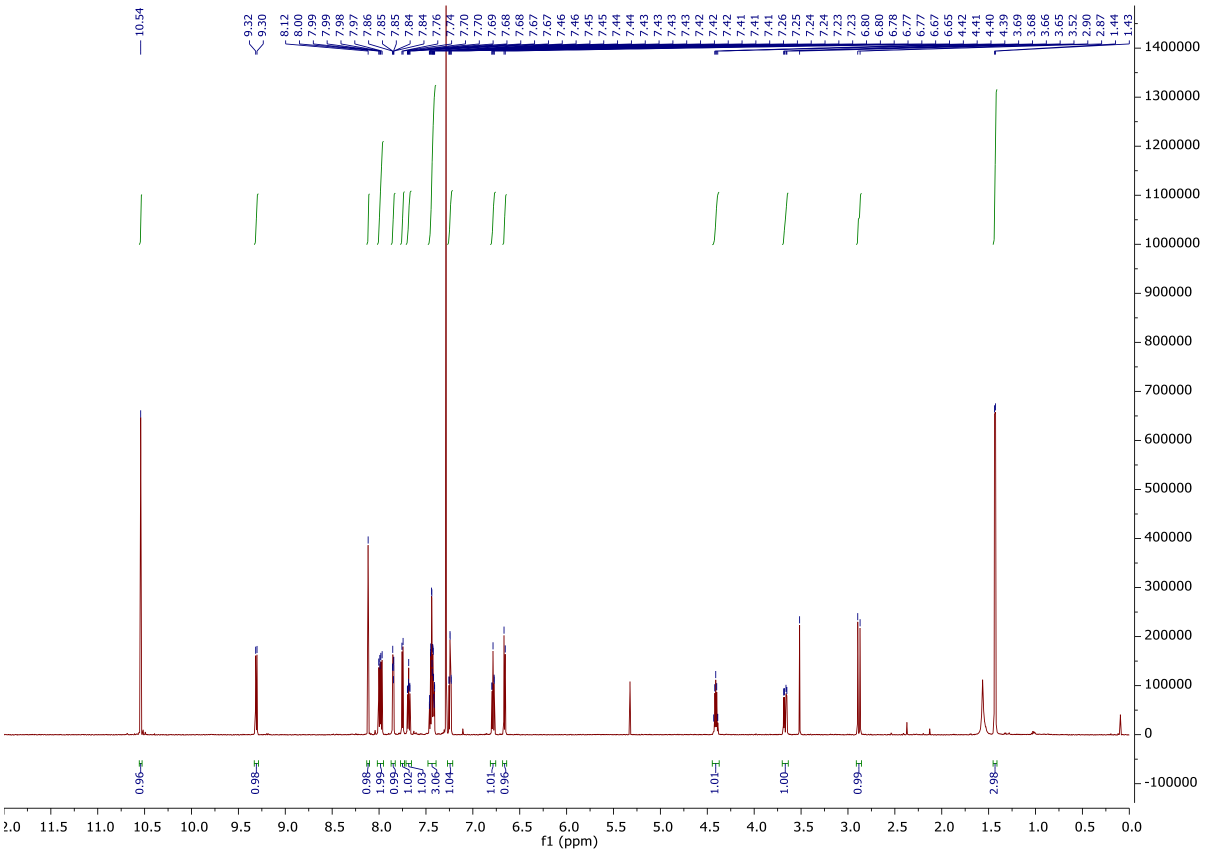

Supplementary Fig. 30. ^{1}H NMR (600 MHz) spectrum of **M2** measured in chloroform-d_1 at 25 °C.

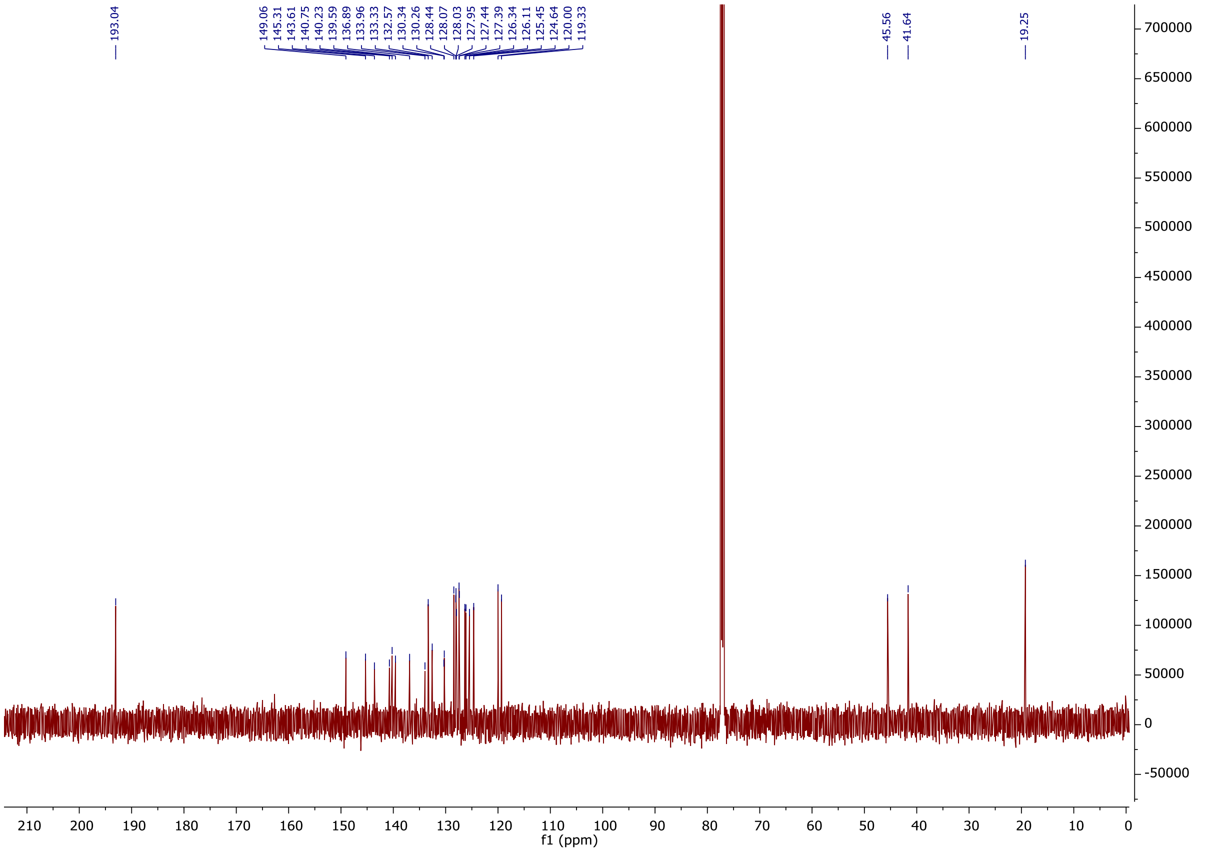

Supplementary Fig. 31. ^{13}C NMR (151 MHz) spectrum of **M2** measured in chloroform-d_1 at 25 °C.

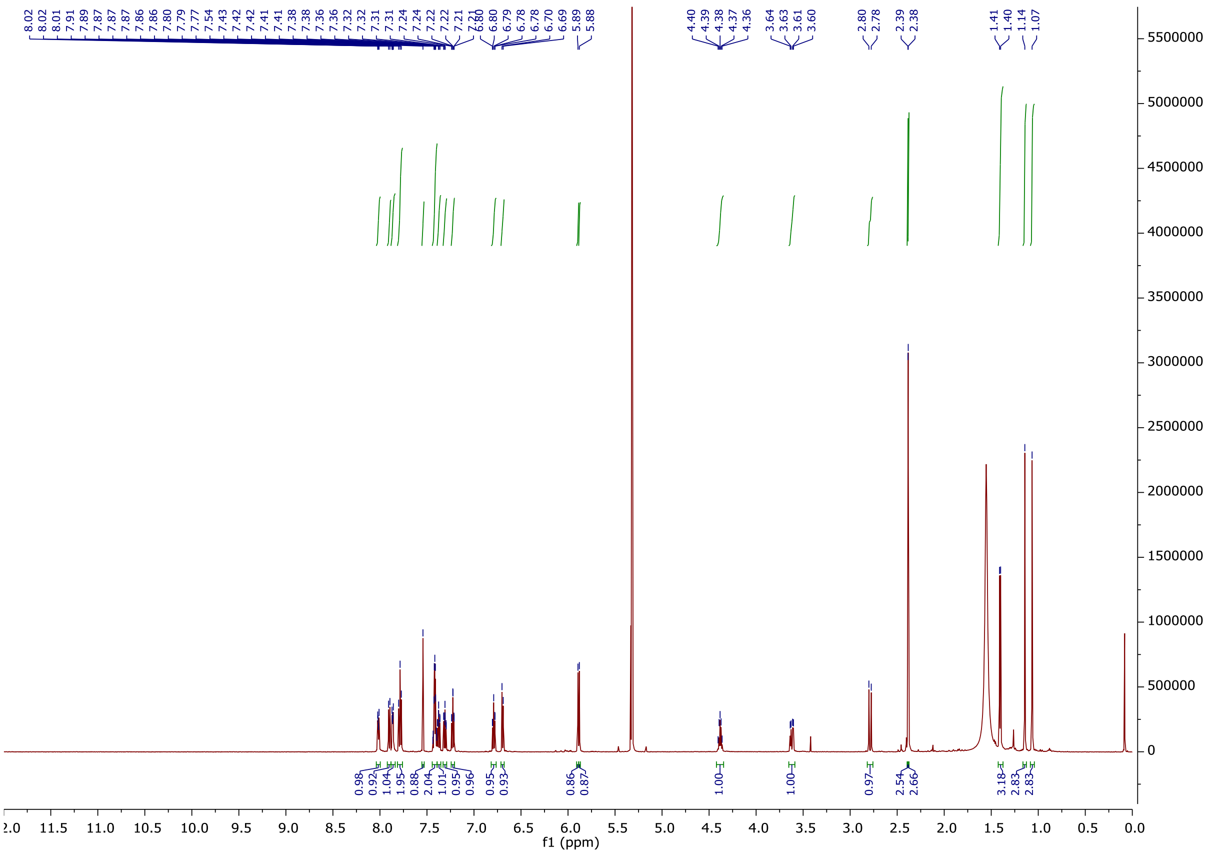

Supplementary Fig. 32. ^{1}H NMR (600 MHz) spectrum of **M3** measured in methylene chloride-d_2 at 25 °C.

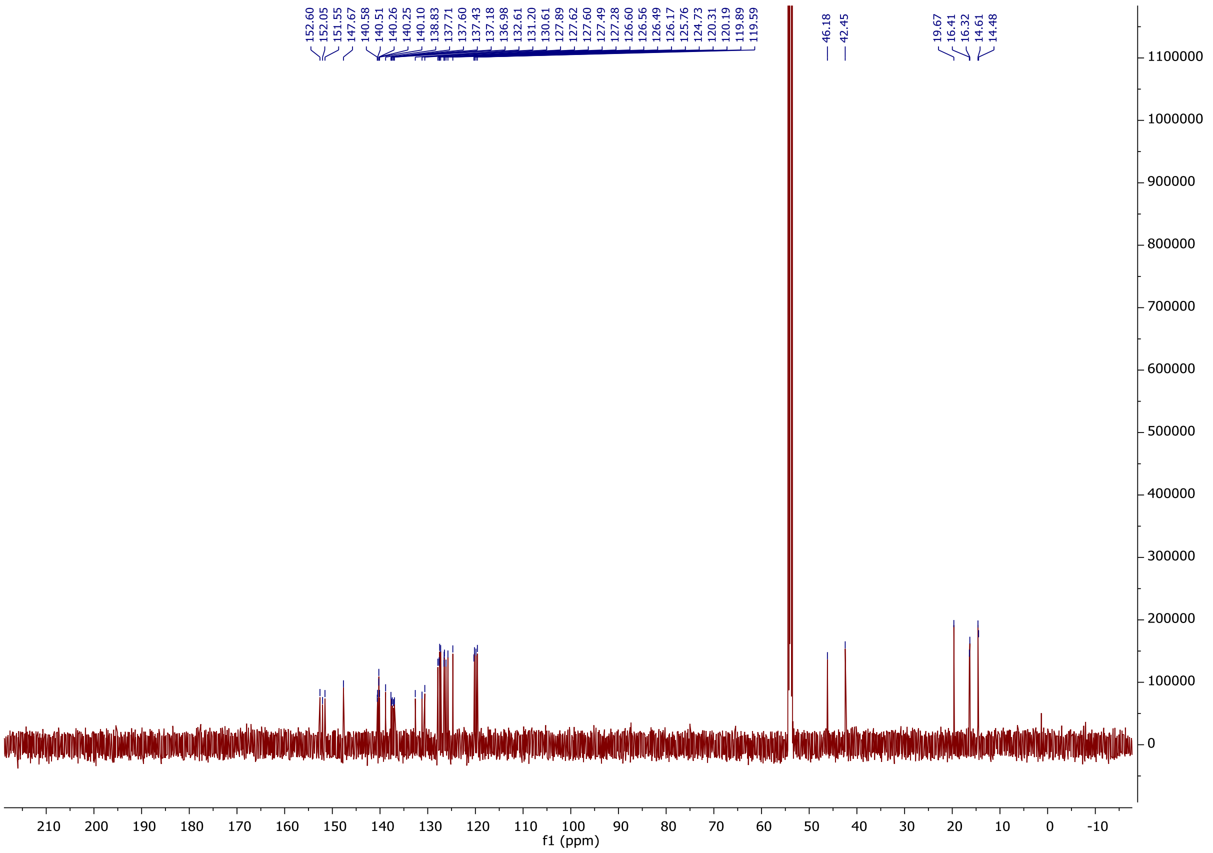

Supplementary Fig. 33. ^{13}C NMR (151 MHz) spectrum of **M3** measured in methylene chloride-d_2 at 25 °C.

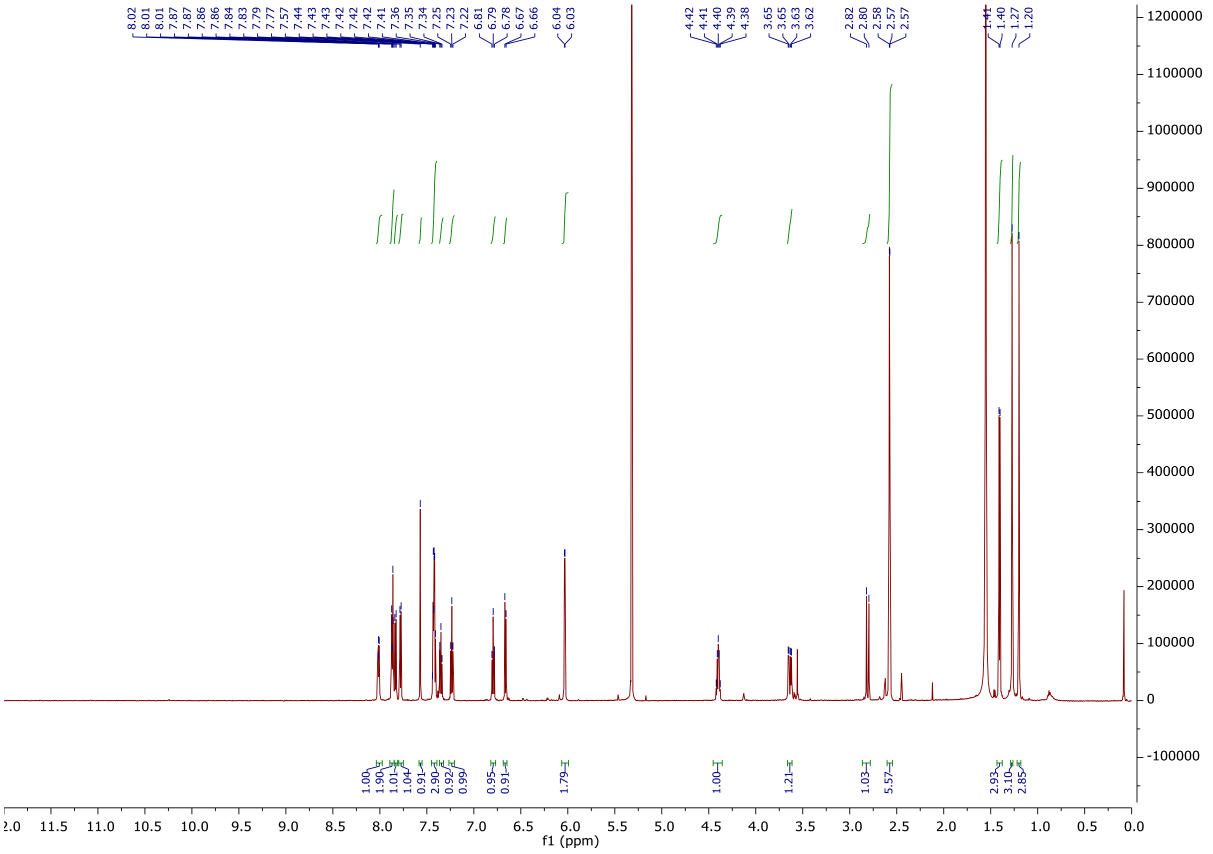

Supplementary Fig. 34. ^{1}H NMR (600 MHz) spectrum of **BODIPY/Motor** measured in methylene chloride-d_2 at 25 °C.

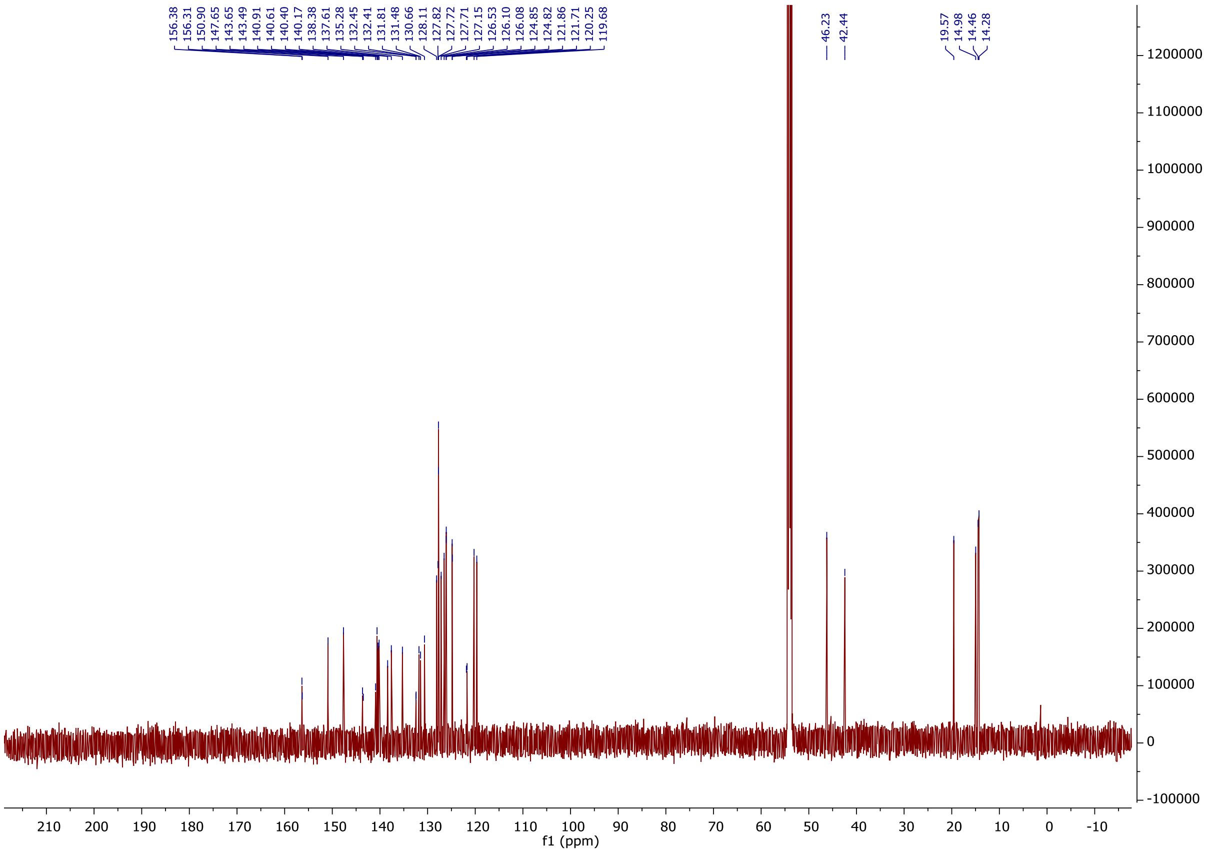

Supplementary Fig. 35. ^{13}C NMR (151 MHz) spectrum of **BODIPY/Motor** measured in methylene chloride-d_2 at 25 °C.

Supplementary References

1. Vicario, J., Walko, M., Meetsma, A. & Feringa, B. L. Fine tuning of the rotary motion by structural modification in light-driven unidirectional molecular motors. *J. Am. Chem. Soc.* **128**, 5127–5135 (2006).

2. Nepomnyashchii, A. B., Bröring, M., Ahrens, J. & Bard, A. J. Synthesis, photophysical, electrochemical, and electrogenerated chemiluminescence studies. Multiple sequential electron transfers in BODIPY monomers, dimers, trimers, and polymer. *J. Am. Chem. Soc.* **133**, 8633–8645 (2011).

3. Roke, D., Stuckhardt, C., Danowski, W., Wezenberg, S. J. & Feringa, B. L. Light ‐ gated rotation in a molecular motor functionalized with a dithienylethene switch. *Angew. Chemie Int. Ed.* **57**, 10515–10519 (2018).

4. Pfeifer, L., Hoang, N. V., Scherübl, M., Pshenichnikov, M. S. & Feringa, B. L. Powering rotary molecular motors with low-intensity near-infrared light. *Sci. Adv.* **6**, eabb6165 (2020).

5. Bruker, APEX3 (V2019.1-0), SAINT (Version 8.40A) SADABS (Version 2016/1). Bruker AXS Inc., Madison, Wisconsin, USA. n.d.

6. Krause, L., Herbst-Irmer, R., Sheldrick, G. M. & Stalke, D. Comparison of silver and molybdenum microfocus X-ray sources for single-crystal structure determination. *J. Appl. Crystallogr.* **48**, 3–10 (2015).

7. Sheldrick, G. M. SHELXT – Integrated space-group and crystal-structure determination. *Acta Crystallogr. Sect. A Found. Adv.* **71**, 3–8 (2015).

8. Sheldrick, G. M. A short history of SHELX. *Acta Crystallogr. Sect. A Found. Crystallogr.* **64**, 112–122 (2008).

9. Strahan, J. *et al.* Modulating absorption and charge transfer in bodipy-carbazole donor–acceptor dyads through molecular design. *Dalt. Trans.* **48**, 8488–8501 (2019).

10. Prieto-Montero, R. *et al.* Methylthio BODIPY as a standard triplet photosensitizer for singlet oxygen production: a photophysical study. *Phys. Chem. Chem. Phys.* **21**, 20403–20414 (2019).

11. Sunahara, H., Urano, Y., Kojima, H. & Nagano, T. Design and synthesis of a library of BODIPY-based environmental polarity sensors utilizing photoinduced electron-transfer-controlled fluorescence ON/OFF switching. *J. Am. Chem. Soc.* **129**, 5597–5604 (2007).

12. Wall, K. P., Dillon, R. & Knowles, M. K. Fluorescence quantum yield measurements of fluorescent proteins: a laboratory experiment for a biochemistry or molecular biophysics laboratory course. *Biochem. Mol. Biol. Educ.* **43**, 52–59 (2015).

13. Brouwer, A. M. Standards for photoluminescence quantum yield measurements in solution (IUPAC Technical Report). *Pure Appl. Chem.* **83**, 2213–2228 (2011).

14. Vicario, J., Meetsma, A. & Feringa, B. L. Controlling the speed of rotation in molecular motors. Dramatic acceleration of the rotary motion by structural modification. *Chem. Commun.* 5910–5912 (2005).

15. Stranius, K., & Börjesson, K., Determining the photoisomerization quantum yield of photoswitchable molecules in solution and in the solid state. *Sci. Rep.* **7**, 41145 (2017).

16. Kuhn, H. J., Braslavsky, S. E. & Schmidt, R., Chemical actinometry (IUPAC Technical Report) *Pure Appl. Chem.* **76**, 2105–2146 (2004).

17. Montalti, M., Credi, A., Prodi, L. & Gandolfi, M. T. Handbook of Photochemistry, CRC Press (2006).

18. Hoops, S., Sahle, S., Gauges, R., Lee, C., Pahle, J., Simus, N., Singhal, M., Xu, L., Mendes, P. & Kummer, U. COPASI—a COmplex PAthway Simulator. *Bioinformatics* **22**, 3067–3074 (2006).

19. Manifar, T., Rezaee, A., Sheikhzadeh, M. & Mittler, S. Formation of uniform self-assembly monolayers by choosing the right solvent: OTS on silicon wafer, a case study. *Appl. Surf. Sci.* **254**, 4611–4619 (2008).

20. Gupta, M. N., Batra, R., Tyagi, R. & Sharma, A. Polarity Index: The guiding solvent parameter for enzyme stability in aqueous-organic cosolvent mixtures. *Biotechnol. Prog.* **13**, 284–288 (1997).

21. Rumble, J. R. CRC Handbook of Chemistry and Physics (99th ed.). (CRC Press, United States, 2018).

22. Conyard, J. *et al.* Ultrafast dynamics in the power stroke of a molecular rotary motor. *Nat. Chem.* **4**, 547–551 (2012).

23. Hall, C. R. *et al.* Ultrafast dynamics in light-driven molecular rotary motors probed by femtosecond stimulated Raman spectroscopy. *J. Am. Chem. Soc.* **139**, 7408–7414 (2017).

24. Conyard, J., Cnossen, A., Browne, W. R., Feringa, B. L. & Meech, S. R. Chemically optimizing operational efficiency of molecular rotary motors. *J. Am. Chem. Soc.* **136**, 9692–9700 (2014).

25. Cnossen, A. *et al.* Driving unidirectional molecular rotary motors with visible light by intra- and intermolecular energy transfer from palladium porphyrin. *J. Am. Chem. Soc.* **134**, 17613–17619 (2012).

26. Sun, W. *et al.* Highly efficient photon upconversion based on triplet–triplet annihilation from bichromophoric annihilators. *J. Mater. Chem. C* **9**, 14201–14208 (2021).

27. Rihter, B. D., Kenney, M. E., Ford, W. E. & Rodgers, M. A. J. Synthesis and photoproperties of diamagnetic octabutoxyphthalocyanines with deep red optical absorbance. *J. Am. Chem. Soc.* **112**, 8064–8070 (1990).

28. Ostroverkhova, O. Handbook of Organic Materials for Electronic and Photonic Devices. (Elsevier Ltd. Netherlands, 2019).

29. Miller, C. C. The Stokes-Einstein law for diffusion in solution. *Proc. R. Soc. Lond. A* **106**, 724-749 (1924).

30. Einstein, A. Über die von der molekularkinetischen Theorie der Wärme geforderte Bewegung von in ruhenden Flüssigkeiten suspendierten Teilchen. *Annalen der Physik* **17**, 549-560 (1905).

31. Richardson, H. H., Carlson, M. T., Tandler, P. J., Hernandez, P. & Govorov, A. O. Experimental and theoretical studies of light-to-heat conversion and collective heating effects in metal nanoparticle solutions. *Nano Lett.* **9**, 1139–1146 (2009).
